# Comprehensive structural, infrared spectroscopic and kinetic investigations of the roles of the active-site arginine in bidirectional hydrogen activation by the [NiFe]-hydrogenase ‘Hyd-2’ from *Escherichia coli*†

**DOI:** 10.1039/d2sc05641k

**Published:** 2023-07-25

**Authors:** Rhiannon M. Evans, Stephen E. Beaton, Patricia Rodriguez Macia, Yunjie Pang, Kin Long Wong, Leonie Kertess, William K. Myers, Ragnar Bjornsson, Philip A. Ash, Kylie A. Vincent, Stephen B. Carr, Fraser A. Armstrong

**Affiliations:** a University of Oxford, Department of Chemistry South Parks Road Oxford UK stephen.carr@rc-harwell.ac.uk fraser.armstrong@chem.ox.ac.uk; b Research Complex at Harwell, Rutherford Appleton Laboratory, Harwell Campus Didcot UK; c College of Chemistry, Beijing Normal University 100875 Beijing China; d Department of Inorganic Spectroscopy, Max Planck Institute for Chemical Energy Conversion Stiftstraße 34-36 45470 Mülheim an der Ruhr Germany; e Univ Grenoble Alpes, CNRS, CEA, IRIG, Laboratoire Chimie et Biologie des Métaux 17 Rue Des Martyrs F-38054 Grenoble Cedex France; f School of Chemistry, The University of Leicester University Road Leicester LE1 7RH UK

## Abstract

The active site of [NiFe]-hydrogenases contains a strictly-conserved pendant arginine, the guanidine head group of which is suspended immediately above the Ni and Fe atoms. Replacement of this arginine (R479) in hydrogenase-2 from *E. coli* results in an enzyme that is isolated with a very tightly-bound diatomic ligand attached end-on to the Ni and stabilised by hydrogen bonding to the Nζ atom of the pendant lysine and one of the three additional water molecules located in the active site of the variant. The diatomic ligand is bound under oxidising conditions and is removed only after a prolonged period of reduction with H_2_ and reduced methyl viologen. Once freed of the diatomic ligand, the R479K variant catalyses both H_2_ oxidation and evolution but with greatly decreased rates compared to the native enzyme. Key kinetic characteristics are revealed by protein film electrochemistry: most importantly, a very low activation energy for H_2_ oxidation that is not linked to an increased H/D isotope effect. Native electrocatalytic reversibility is retained. The results show that the sluggish kinetics observed for the lysine variant arise most obviously because the advantage of a more favourable low-energy pathway is massively offset by an extremely unfavourable activation entropy. Extensive efforts to establish the identity of the diatomic ligand, the tight binding of which is an unexpected further consequence of replacing the pendant arginine, prove inconclusive.

## Abstract 2

Changing the conserved active-site arginine of [NiFe]-hydrogenases into a lysine greatly lowers the rates of catalytic H_2_ activation in each direction and results in the extremely tight binding of a diatomic ligand.

## Graphical abstract

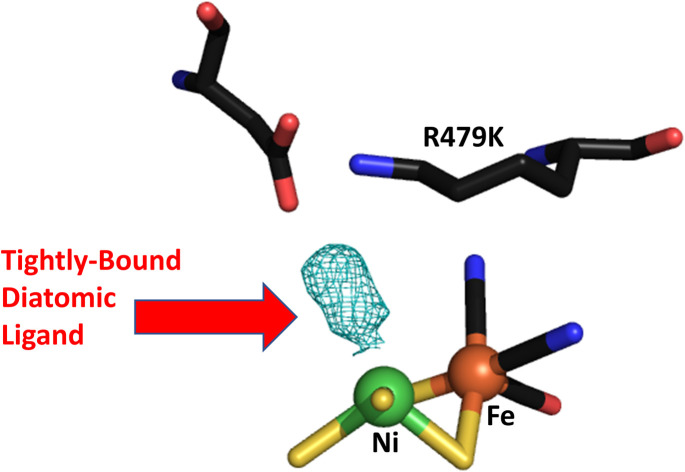

## 1. Introduction

Hydrogenases, which catalyse the production and oxidation of molecular hydrogen (H_2_), are not only essential for many microorganisms but also serve as paradigms for understanding, in detail, the importance of proton-coupled electron transfer (whether concerted or not) in enzyme mechanisms.^1^ Attached to an electrode, hydrogenases display highly efficient H_2_ activation,^2^ and as ‘reversible’ electrocatalysts,^1^ they can inspire the atomic-level design of novel catalysts for future renewable energy technologies, most obviously in the development of green H_2_ as a major resource and fuel.^3–6^ The two membrane-bound group 1 (ref. 7) [NiFe]-hydrogenases of *Escherichia coli* serve as exemplars for catalyst design since they are highly similar, structurally, yet display subtly diverging catalytic properties. Hydrogenase-1 (‘Hyd-1’) is active only in the H_2_ oxidation direction at neutral pH,^8^ but combines unidirectionality with the second desirable property of O_2_-tolerance, defined as the ability to sustain H_2_ oxidation activity in the presence of O_2_.^9–11^ In contrast, ‘Hyd-2’ displays typical O_2_ sensitivity, but is capable of efficient H_2_ production as well as H_2_ oxidation under standard physiological conditions.^12,13^

**Fig. 1:**
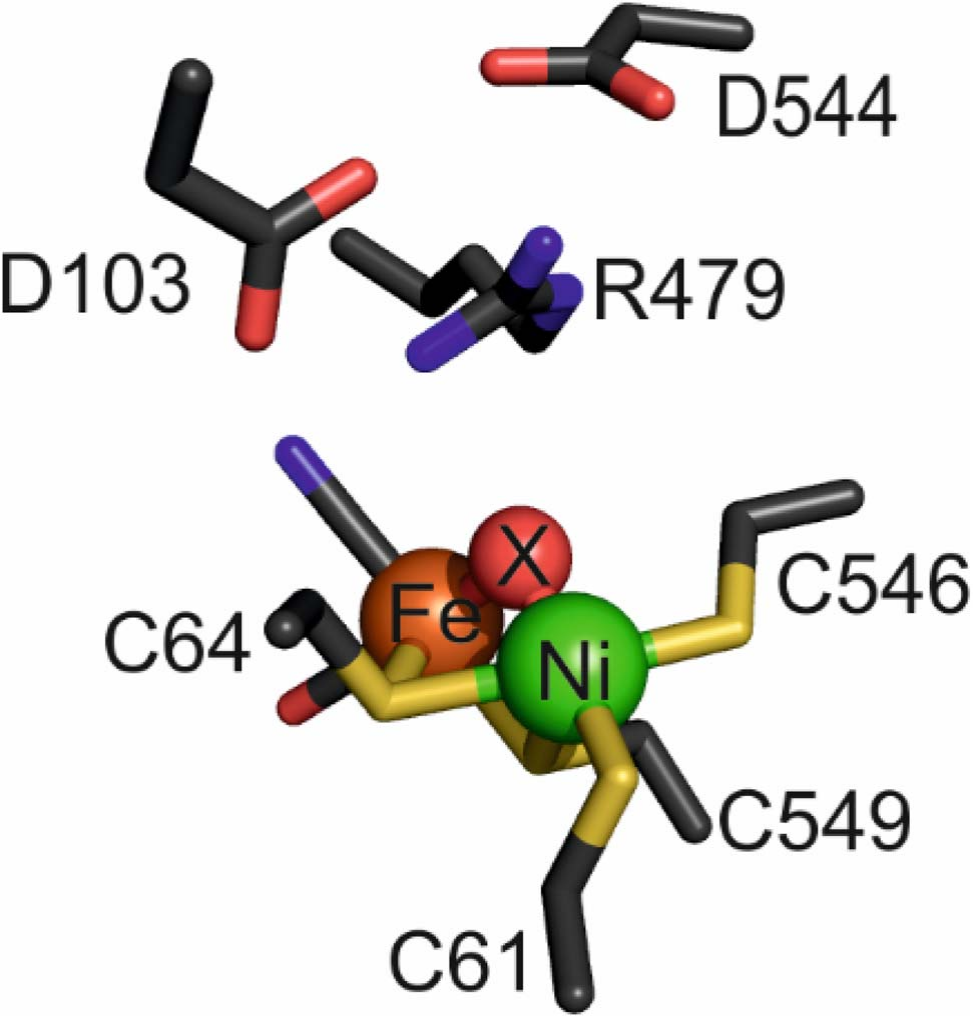
The active site of as-isolated Hyd-2 (pdb 6EHQ) including the ‘canopy’ of conserved amino acids above the bimetallic cofactor. The bridging ligand, ‘X’, is a hydroxide in this instance but is replaced by a hydride following reduction of the enzyme under H_2_.

The buried active site of [NiFe]-hydrogenases contains a highly conserved inner coordination shell: the Ni atom is coordinated by four strictly conserved cysteine thiolates, two of which form a bridge to a Fe(CO)(CN)_2_ fragment that appears to remain as a low-spin Fe^II^ site during catalysis,^14,15^ while the other two bind in a terminal manner (Fig. 1). Hydrogenases are usually inactive upon aerobic isolation and characterised by the presence of a hydroxide ligand located in a bridging position between the Ni(iii) and Fe(ii) atoms (Ni-B, ‘ready’ state) and with additional oxygenation of a cysteine ligand (Ni-A, ‘unready’).^2,16^ Research into synthetic, structural analogues of hydrogenases^3,6,16–18^ as well as a Ni-substituted rubredoxin^19,20^ have demonstrated that the inner coordination shell alone is ineffective at catalysing reversible and bidirectional H_2_ activation.^21,22^ ‘Bidirectionality’ refers to the ability of the catalyst to function in either the proton reduction or H_2_ oxidation direction depending on the applied potential, whereas ‘reversibility’ refers to the ability of the catalyst to be bidirectional with minimal overpotential requirement either side of the equilibrium potential. It follows therefore that a reversible catalyst is always bidirectional, but a bidirectional catalyst may not necessarily display reversibility.^23^ Looking to the outer coordination shell for further insight, a number of catalytically-important, non-coordinating amino acids have been identified in various [NiFe]-hydrogenases, and two of these appear crucial for activity. One such residue is a glutamate^24–26^ (E28/E14 in Hyd-1/Hyd-2 respectively), the carboxylate of which lies close (<4 Å) to the Ni-coordinating terminal cysteine C576/C546;^25,27^ the other is a strictly conserved ‘pendant’ arginine^27,28^ (R509/R479) that is located in a canopy immediately above the catalytic metals.

It has long been proposed that the H_2_ molecule is cleaved or formed heterolytically, *i.e.* involving a transition state of the form H^−^⋯H^+^, and studies of reduced catalytic intermediates reveal a hydridic H-atom coordinated in a bridging position between Ni and Fe.^2,16,29–31^ It has also been proposed that the primary base for abstraction of H^+^ during the initial heterolytic cleavage is the Ni–S thiolate of C576/546 (Fig. 1)^16^ and it has been postulated that this proton is then transferred to the nearby glutamate residue (E28/E14).^26,32,33^ However, recent work has shown that the glutamate in *E. coli* Hyd-1 is not necessary for the initial deprotonation step of H_2_ activation;^34^ furthermore, alteration of this residue does not render the enzyme completely inactive as in other homologues.^26,33–35^

The importance of the pendant arginine (R509/R479) is not surprising considering its conservation and its close proximity and position above the intermediate hydrido ligand. The exact role of the arginine is disputed in the literature and only recently have experiments been performed that were able to demonstrate a crucial mechanistic relevance across various enzyme counterparts and techniques.^27,28,36,37^ Studies based on computational analyses^37^ and kinetic and spectroscopic analyses^36^ suggest critical roles for the arginine in polarising H_2_ for proton abstraction by an active site cysteine, controlling substrate/ligand access, conferring conformational rigidity to keep the active site in a catalytically-accessible state, and mediating proton transfer between tautomeric reduced active-site states. In our recent work, based on structural and kinetic data for the arginine-to-lysine substituted variant of Hyd-1 (R509K), we postulated a direct role of the guanidine/guanidinium head group of R509 to act as a primary Lewis base during catalysis and, together with the active site metal (Lewis acid), activate H_2_ as a Frustrated Lewis Pair (FLP)^38,39^ – an atomic-level mechanism that is generally accepted for [FeFe]-hydrogenases^40,41^ and the best functional mimics.^3^

As with other examples where arginine plays a direct role in catalysis, such a proposal carries the assumption that the high p*K*_a_ of the guanidinium headgroup is somehow beneficial: either it is significantly lowered from that of free arginine (>12)^42^ by the local environment or there is direct, transient coupling of deprotonation to other steps involving a strong base, in this case H–H bond formation or cleavage.^27,43–52^ Proton transfer to an adjacent and strongly basic intermediate, such as a metal hydrido species, could be a favourable step in a sequence.^53^ For several enzymes, including various flavoenzymes and ascorbate peroxidase, there is a compelling case for direct participation of an arginine guanidinium/guanidine group as a proton relay.^43–48,54–57^ These examples include recent neutron diffraction studies revealing a deprotonated (guanidine) side chain in an environment otherwise lacking any special features that would stabilise such a state.^54^ Such direct evidence proves that calculations alone cannot rule out a direct acid–base role for arginine and it is thus safe to say that any general objection to a direct role for arginine in catalysis is untenable.

We recently designed a unique overexpression system for Hyd-2,^12,25^ greatly facilitating the ability to produce variants analogous to those available for Hyd-1, thus allowing the pendant arginine question to begin to be addressed with a standard, O_2_-sensitive [NiFe]-hydrogenase that is naturally proficient in H_2_ production as well as oxidation.^12,13^ We therefore substituted R479 for lysine in Hyd-2 and carried out a detailed study of the structure and kinetics of the R479K variant to make a ‘like-for-like’ comparison with Hyd-1 R509K. The crystallographic data and protein film electrochemistry results for R479K confirm the vital importance of the pendent arginine for catalytic H_2_ production as well as oxidation. Additionally, replacing the arginine by lysine allows formation of an inactive oxidised state of the enzyme, in which the vacant coordination site on the Ni is occupied by a tightly-bound diatomic ligand.

## 2. Methods

### 2.1 Molecular biology

Cloning steps for the overproduction of Hyd-2 have been described previously.^12^ The R479K mutation was made on pMAK-*hybC* plasmid constructs, and following confirmation by DNA sequencing, the codon changes were each transferred to strain HJ001-hyp (Table S1†).^27,58,59^

### 2.2 Enzyme production and purification

All protein was over-produced from *E. coli* MC4100-derived K-12 strain ‘HJ001-hyp’^12,59^ or the relevant variant strain transformed with plasmid pO^C^ for over-expression of the *hybO* gene containing a 3′-hexahistidine tag (Table S1†).^12^ This system produces enzyme that is not translocated to the membrane.^25^ Enzymes were purified as previously described.^12^ Briefly, the catalytic cores of native Hyd-2 and R479K (membrane extrinsic subunits, HybOC) were isolated from the cell cytoplasm by Ni-affinity chromatography. A second purification step was performed: the resulting product was used for steady-state solution kinetics and PFE experiments, and to prepare samples for crystallography, EPR and IR spectroscopy. For clarity, the native over-produced Hyd-2 (otherwise abbreviated Hyd-2-NOP) and variant over-produced R479K enzymes will be referred to as ‘Hyd-2’ and ‘R479K’ respectively throughout this paper.

### 2.3 Protein film electrochemistry

All electrochemical experiments were performed with a gastight, water-jacketed electrochemical cell contained inside an anaerobic (N_2_) glovebox (MBraun or Belle Technologies, O_2_ <3 ppm). Precise gas mixtures were created from component gases of the highest purity (BOC; H_2_ >99.99% purity, O_2_ 99.5% purity, CO 99.97% purity and Ar 99.998% purity) using mass flow controllers (Sierra Instruments). Electrochemical control was achieved using an Autolab potentiostat (PGSTAT128N) controlled by Nova software (EcoChemie). The pyrolytic graphite ‘edge’ (PGE) working electrode (area 0.03 cm^2^) was rotated at a constant speed (*ω* > 1000 rpm) to minimise any limitations from H_2_ mass transport either during its oxidation (as substrate) or production (as inhibitor). A Pt wire (counter) and saturated calomel electrode (SCE) reference completed the circuit, the SCE electrode being housed in a side arm containing 0.1 M NaCl that was electrochemically connected to the main compartment *via* a Luggin capillary. All potentials quoted have been converted to the Standard Hydrogen Electrode (SHE) scale using the correction *E*_SHE_ = *E*_SCE_ + 0.241 at 25 °C.^60^

To prepare each enzyme film, the PGE electrode was abraded with sandpaper (P400 Hookit) and thoroughly rinsed with ultrapure water. Enzyme (1–5 µg) was applied to the working electrode by pipetting directly onto the electrode surface, or immersing the electrode in a dilute enzyme ‘forming’ solution whilst monitoring activity as a function of potential in 100% H_2_. All enzyme films were prepared in this way unless otherwise stated, *i.e.*, for some experiments with R479K a ‘modified’ electrode system was used, as previously described^28,61,62^ wherein enzyme is covalently linked to pyrene–butyric acid on multi-walled carbon nanotubes. All enzyme films were extensively ‘activated’ (*i.e.*, the electrode was poised at a reducing potential under H_2_ until no further increase in activity is observed) to reduce oxidised species formed during aerobic purification. The electrode potential was stepped between −0.66 V (300 s) where activation is faster for readily activated state(s) and −0.40 V (15 s) to monitor the H_2_ oxidation current. The progress of activation under H_2_ was monitored across a wider potential range by cyclic voltammetry at a scan rate of 30 mV s^−1^. Where necessary, the activation period was extended for several hours or overnight.

### 2.4 Steady-state activity measurements

Prior to steady-state activity measurements, enzyme stocks were reductively activated by purging with 100% H_2_ (BOC, >99.99% purity) for 12–18 hours either in the absence or presence of electrochemically reduced methyl viologen (1 mM to give >1400-fold excess). The initial velocity of H_2_ oxidation by R479K was measured as previously described^12,28^ using 2 mM benzyl viologen as electron acceptor (*E* = −358 mV, *ε* = 8.4 mM^−1^ cm^−1^ at 604 nm (ref. 63)) in H_2_-saturated, 100 mM potassium phosphate buffer at pH 6.0. The H_2_-only-activated enzyme stock was then purged with Ar for at least 12 hours. The H_2_ production activity was measured using reduced methyl viologen as electron donor (*E* = −446 mV, *ε* = 13.9 mM^−1^ cm^−1^ at 604 nm (ref. 63)) as previously described^12,13^ in Ar-saturated 100 mM potassium phosphate buffer at pH 6.0. Only enzyme activated in the absence of methyl viologen was used for H^+^ reduction activity measurements.

### 2.5 Protein crystallization

Enzymes were crystallized using conditions described elsewhere.^12^ Purified R479K was concentrated to 5 mg mL^−1^ (assessed by Bradford assay,^64^ Sigma) using centrifugal concentrators with a 50 000 molecular weight cut-off (Vivaspin). The as-isolated enzyme was transferred to an anaerobic glove box for crystallisation without further treatment; reduced enzyme was obtained by exposing the enzyme solution to a flow of 100% H_2_ for 20 h at 4 °C in an anaerobic (N_2_) glovebox (Belle Technologies, <0.3 ppm O_2_); CO-inhibited enzyme was produced by further exposure of reduced enzyme to 100% CO (BOC 100% purity) for 20 minutes. Re-exposure to O_2_ following extensive reductive activation was performed using 100% O_2_ (BOC 99.9999% purity). Crystals of each form of R479K were obtained using the sitting drop vapour diffusion technique: 1 µL of protein solution was mixed with an equal volume of de-oxygenated crystallization solution (100 mM Bis-Tris pH 5.5–5.9, 200 mM MgCl_2_ and 18–22% w/v PEG 3350). Crystallization trays were set up manually under anaerobic conditions (Belle Technologies, <0.3 ppm O_2_) and incubated at 23 °C (ambient glovebox temperature). Regardless of gas treatments, long rod-shaped crystals appeared after approximately 24 h; these were manually transferred to an anaerobic cryo-protecting solution (100 mM Bis-Tris pH 5.5–5.9, 200 mM MgCl_2_, 23% w/v PEG 3350 and 15% v/v glycerol) for 30–60 s before flash-cooling in liquid nitrogen.

### 2.6 X-ray data collection and structure determination

X-ray diffraction data were collected at beamline i04-1, Diamond Light Source, at a wavelength of 0.92 Å using a Pilatus 6M hybrid pixel array detector. All data were collected at 100 K using a helical line scan to minimise photo-reduction. Data reduction was performed using DIALS^65^ and Aimless.^66^ Initial phase estimates were generated by molecular replacement within Phaser^67^ using the Hyd-2 pdb file 6EHQ^12^ as a search model. The electron-density maps were inspected using COOT,^68^ residue 479 was manually changed to lysine and crystallographically ordered water molecules assigned. The resulting model was refined using REFMAC5 (ref. 69) followed by additional rounds of manual rebuilding and refinement until no further improvements were possible. Structure analysis was performed using a combination of CastP,^70^ Caver2 (ref. 71) and Pymol.^72^

### 2.7 EPR spectroscopy

Continuous-wave EPR spectroscopy was carried out on as-isolated R479K samples at concentrations of 90–400 µM in 100 mM MES, 100 mM NaCl, 10% glycerol, pH 6.0. An X-band (9.1–9.9 GHz) Bruker EMX spectrometer equipped with a low-temperature helium flow cryostat (Oxford Instruments) was used to make measurements at 10 K and 80 K. Thin-walled quartz EPR tubes (Fluorochem) were used, high precision (1.6 mm) tubes for the 400 µM sample and standard 4 mm tubes for all other samples. Data were collected at a microwave power of 2.00 mW and a modulation amplitude of 1.0 mT. Spectra were averaged over >12 hours for the 400 µM sample. EPR simulations were performed using the EasySpin 5.2.25 routines for a Matlab scripting environment (The Mathworks, Natick, NJ).^73^

### 2.8 Infrared spectroscopy

Solution-based transmission infrared (IR) experiments were performed on 5–6 µL samples of 1 mM R479K in 20 mM Tris, pH 7.2, 150 mM NaCl sandwiched between two CaF_2_ windows (31.8 mm diameter, 1.5 mm thickness, Crystan) separated by a 25 µm Teflon spacer (Kromatek Ltd) coated with vacuum grease and enclosed in a commercial IR transmission cell (PIKE). Spectra were measured at room temperature inside an anaerobic, dry glovebox (Glove Box Technology Ltd, <2 ppm of O_2_, <85 °C dew point) on a Bruker Vertex 80 FTIR spectrometer equipped with a mercury cadmium telluride (MCT) detector cooled with liquid N_2_. Each spectrum was recorded as an average of 1024 interferograms using OPUS software (Bruker) in the double-sided, forward–backward mode with a resolution of 2 cm^−1^, an aperture setting of 1 mm and a scan velocity of 20 Hz. Data were processed using home-written routines in MATLAB™ and OPUS software (Bruker). The figures were prepared in Origin graphical software (OriginLabCorp).

All samples were stored under liquid nitrogen or at −80 °C in a sealed anaerobic vial until use. Prior to measurements, as-isolated samples were reductively activated: large volume dilute samples (>50 µL, ∼100 µM) were flushed with humidified 100% H_2_ for 24 hours in an anaerobic glovebox (Belle Technologies, O_2_ <3 ppm), whereas small-volume, concentrated samples (<10 µL at ∼1 mM) were activated under 4 bar H_2_ (Büchi Tinyclave pressure vessel) inside an anaerobic glovebox (Glove Box Technology Ltd, <3 ppm O_2_) for 19 hours. The various gas treatments of samples for identification of the diatomic ligand included an in-line ultra-high capacity O_2_ scrubber (Restek™ Super-Clean™) and were performed as explained in Results. All gases were obtained from BOC (H_2_ >99.99% purity, O_2_ 99.5% purity, CO 99.97% purity and Ar 99.998% purity).

In-crystallo microspectroscopic electrochemical IR experiments were performed using single crystals of R479K enzyme under electrochemical control as previously described.^74^ Prior to any experiments, regardless of the oxidised/reduced state of the samples, the crystals were reductively activated, electrochemically, in a microspectroscopic electrochemical cell at *ca.* −600 mV until no further changes were observed in the *v*_CO_ and *v*_CN_ bands in the IR spectra over a period of approximately 10 min. Typically, this activation period was at least 1 h. Unless otherwise stated, all in-crystallo microspectroscopic electrochemical data were collected using a Vertex 80v FTIR spectrometer with a Hyperion 3000 IR microscope (Bruker) at Diamond Light Source, MIRIAM beam line B22. The high-sensitivity photovoltaic MCT 50 µm pitch detector was cooled using liquid N_2_. The stage of the IR microscope was contained in an N_2_-purged Perspex housing to minimise contamination by atmospheric O_2_ and water vapour. Each spectrum was recorded using OPUS software (Bruker) in the double-sided, forward backward mode as an average of 512 interferograms at 80 kHz scanner velocity and 4 cm^−1^ resolution using a 6 mm aperture. Electrochemical control was achieved using an Autolab 128N potentiostat and Nova 1.10 software (Metrohm). All subsequent in-crystallo IR and electrochemical data analysis was performed using Origin graphical software (OriginLabCorp).

### 2.9 QM/MM calculations

QM/MM geometry optimizations were performed using the ASH multiscale modelling program (https://ash.readthedocs.io), version 0.7, developed by R. Bjornsson. ORCA version 5.0 (ref. 75) was used as QM program and the OpenMM library version 7.5 (ref. 76) as MM program, both programs being interfaced to ASH. The CHARMM36 force field^77^ was used to describe the protein and the TIP3P forcefield^78^ to describe water, with a simple nonbonding model for the metal clusters (based on Hirshfeld-calculated atom charges and Lennard-Jones parameters for sulfide from CHARMM). An additive QM/MM expression was used, with electrostatic embedding to describe the QM-MM electrostatic interaction, linkatoms used to cap the QM-MM boundary and a charge-shifting scheme as implemented in ASH. The QM-region was described using density functional theory with the r^2^SCAN functional^79–81^ and the D4 dispersion^82^ correction with a ZORA^83,84^ scalar relativistic Hamiltonian (polarized by the MM point charges). The relativistically recontracted ZORA-def2-TZVP basis set^85,86^ was used. The resolution of identify approximation^87^ was used to speed up Coulomb integrals using a decontracted Coulomb-fitting basis set^88^ (SARC/J keyword in ORCA). Calculated vibrational frequencies were scaled by the factor 0.9781. This scaling factor was derived from a calculation of free CO at the same level of theory compared to the experimental frequency. Precise details of all computational methods are provided as ESI.†

## 3. Results

### 3.1 Production of R479K

All enzymes were overproduced and the R479K mutation was incorporated into the overexpression system as reported elsewhere.^12^ Previous investigations revealed that the enzymatic characteristics of native, over-produced Hyd-2 are identical with those of natively (chromosomally) expressed Hyd-2; therefore differences in enzymatic parameters from over-produced variants can be attributed to the point mutation.^12^ The variant protein was over-produced to similar yields and purity as over-produced native Hyd-2 and both enzymes purified as the HybOC dimer (Fig. S1†).

### 3.2 Steady-state solution activities

The steady-state H_2_ oxidation and H^+^ reduction activities were measured for R479K at pH 6.0 (Table 1) and are clearly much lower than observed for Hyd-2.^12^ However, in view of the difficulty in removing the diatomic ligand bound tightly to as-isolated R479K (see later), it was important to establish the fraction of enzyme that is actually active. For H_2_ oxidation, R479K that had been activated under H_2_ in the presence of reduced methyl viologen showed an approximately five-fold increase in rate compared to enzyme that had been activated without methyl viologen. Judging from the data in Table 1 (which reports the rates found in the absence of reduced methyl viologen to allow for direct comparison to other literature) we could therefore be confident that regardless of the method of activation, the H_2_ oxidation activity of R479K lies between 2-10% of the native enzyme. For proton reduction, the rate of the R479K variant was less than 0.4% that of native Hyd-2, having activated the enzyme in the presence of H_2_ alone due to the difficulty in removing the excess methyl viologen from the activated sample used for H_2_ oxidation measurements. Given the activity range achieved for H_2_ oxidation we applied this five-fold difference to the H^+^ reduction activities measured to give a range of H^+^ reduction activity for R479K that is 0.4–2% of Hyd-2.

**Table 1:** Enzymatic parameters measured by steady-state solution assays and PFE experiments. Standard conditions are pH 6, 30 °C for PFE measurements, unless otherwise stated. Steady-state assays were performed at room temperature (25 °C). For direct comparison, Hyd-1 data are included^j^

Parameter	Native Hyd-2 (ref. 12)	Hyd-2 R479K	Native Hyd-1 (ref. 28)	Hyd-1 R509K^28^
H_2_ oxidation steady-state turnover rate (s^−1^)	218.0 ± 17.0^a^	5.0 ± 1.0^a^	257.4 ± 30.4^b^	3.3 ± 1.1^b^
H^+^ reduction steady state turnover rate (s^−1^)	7.0 ± 1.0^c^	0.03 ± 0.01^c^	n.d.	n.d.
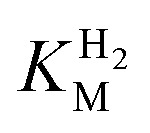 (µM)	23.0 ± 7.0^d^	214.0 ± 45.0^d^	19.8 ± 4.1^e^	6.5 ± 1.1^e^
Δ*H*^‡^ (kJ mol^−1^) H_2_ oxidation	43.7 ± 2.0^f^	23.0 ± 2.6^f^	44.7 ± 0.9^g^	39.4 ± 1.4^g^
Δ*H*^‡^ (kJ mol^−1^) H^+^ reduction	39.1 ± 0.6^h^	38.1 ± 0.5^h^	n.d.	n.d.
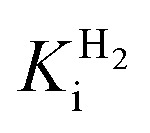 at −0.660 V (µM)	240.0 ± 10.0	1077.0 ± 96.0	n.d.	n.d.
*K*_i_^CO^ at −0.175 V (µM)	3.1 ± 0.6^i^	3.8 ± 0.5^i^	n.d.	n.d.

a Measured with benzyl viologen (*E* = −358 mV (ref. 63)).

b Measured with methylene blue (*E* = 11 mV (ref. 63)).

c Measured with methyl viologen (*E* = −446 mV (ref. 63)).

d Measured at −0.175 V.

e Measured at 0 V.

f Measured at −0.200 V.

g Measured at 0 V.

h Measured at −0.45 V.

i Measured at 20 °C; n.d. not determined.

j N.B. all absolute steady-state turnover rates reported here were measured using enzyme activated in the presence of H_2_ alone, *i.e.* in the absence of reduced methyl viologen.

### 3.3 Protein film electrochemical analysis of kinetic parameters

#### Electrocatalytic window and catalytic bias

**Fig. 2:**
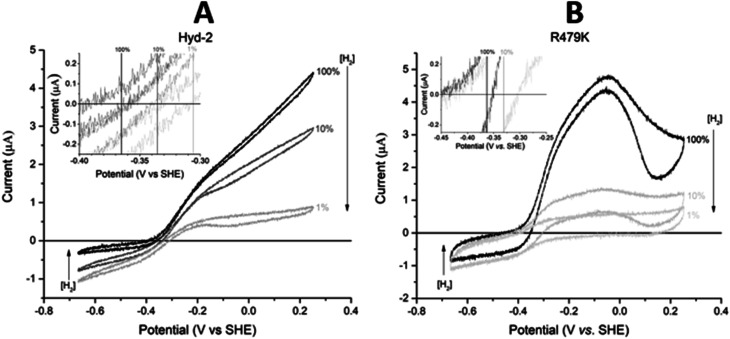
Cyclic voltammograms of (A) Hyd-2 and (B) R479K. The CVs cross the zero-current axis close to the equilibrium potential predicted by the Nernst equation (inset). Other conditions: 5 mV s^−1^, 30 °C, *ω* = 3000 rpm, pH 6.0, carrier gas Ar, total gas flow rate of 1000 sccm (standard cubic centimetres per minute). Note that the absolute currents of each enzyme cannot be used to assess any differences in catalytic turnover since the enzyme coverage of the electrodes are not known in either case, and the enzyme film has been adjusted (see text).

From simple cyclic voltammograms (CVs) recorded at pH 6.0, 30 °C (Fig. 2B), it was immediately evident that the R479K variant was active in both the H_2_ oxidation and H^+^ reduction directions (‘bidirectionality’). As with native Hyd-2 (Fig. 2A) the small current response in the H^+^ reduction direction increased as the H_2_ level was lowered. It should be stressed that while electrochemistry only reports on active enzyme, the voltammogram provides a valuable, characteristic electrochemical profile of both bidirectionality and reversibility as a continuous function of potential, *i.e.* the thermodynamic driving force (Fig. S2†).^1,2,89,90^ Comparing the two methods, solution studies yield values for empirical activity (rate/total amount of enzyme): in contrast, even empirical activity is difficult to address in PFE because the actual electroactive coverage is rarely known.^91,92^ The difference in steady-state catalytic rate between Hyd-2 and R479K is not evident in PFE experiments reported herein since all films have been deliberately adjusted to provide a similar maximum current, *e.g.* by using a more dilute sample of Hyd-2, or by physically removing most of the adsorbed Hyd-2 using paper towel or cotton wool.^91^ This procedure allows for direct comparison of the electrocatalytic profiles of the two enzymes without the need for normalization or scaling of current outputs, which can mask subtle external influences, such as the rate being limited by mass transport of reactants. When H_2_ is present, the current cuts sharply through zero as the reaction direction switches from H^+^ reduction to H_2_ oxidation (the test for reversibility^1,93^) and the zero-current potential is close to the equilibrium value calculated from the Nernst equation (−0.36 V at 30 °C pH 6.0 and 100% H_2_). As expected, the steady-state H_2_ oxidation activity increases as the potential is raised above the equilibrium value but then quickly decreases as the enzyme oxidatively inactivates, the effect being reversed during the sweep to lower potential.^2^ Such a marked level of anaerobic oxidative inactivation is not seen for Hyd-2 (Fig. 2).^13^

#### Michaelis–Menten kinetics and product inhibition

**Fig. 3:**
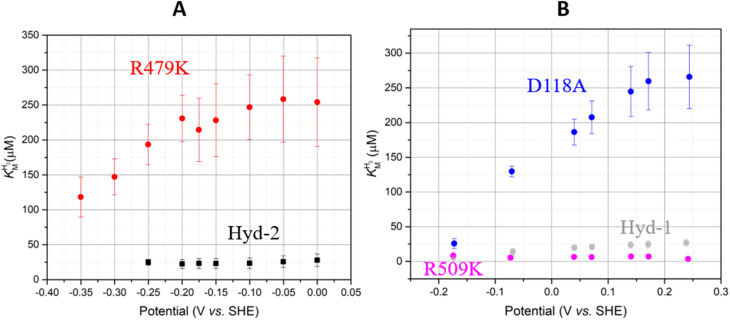
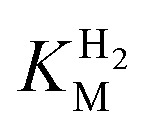
 for (A) Hyd-2 and R479K is plotted next to the reported 
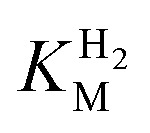
 of (B) Hyd-1, R509K, and D118A.^28^
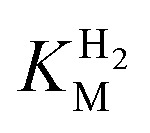
 is calculated from Hanes–Wolf or Michaelis Menten plots populated with data from 5 mV s^−1^ scans at 10 different H_2_ concentrations (0.3–100%), see ESI.† Films were returned to 100% H_2_ every fourth scan to account for film loss. Error bars represent the standard deviation of three repeats. Other conditions: 30 °C, *ω* = 3000 rpm, pH 6.0, total gas flow rate of 1000 sccm.

The 
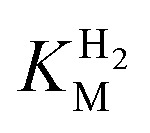
 values (Table 1) at pH 6.0 and 30 °C for Hyd-2 and R479K were determined from currents measured at different potentials in cyclic voltammograms^2,28,94^ recorded at a scan rate of 5 mV s^−1^ over a range of H_2_ concentrations (Fig. S3†). The conditions were returned to 100% H_2_ every fourth cycle to correct for slow film loss^94^ during the course of the experiment. Results are shown in Fig. 3 alongside relevant data obtained for *E. coli* Hyd-1, to allow comparisons to be made. For Hyd-2, 
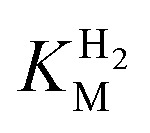
 (23 ± 7 µM at −0.175 V) does not change significantly with potential: in contrast, R479K shows a much higher 
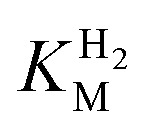
 (214 ± 45 µM at −0.175 V) with a significant potential dependence (Fig. 3A), the 
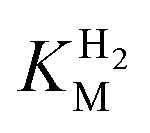
 value increasing two-fold between −0.35 and −0.20 V. As seen in Fig. 3B, the large increase in value for R479K relative to Hyd-2 is not observed for the corresponding mutation in Hyd-1 (R509K), but is observed for the Hyd-1 D118A variant (D118 corresponds to D103 in Hyd-2). Substantial anaerobic inactivation of R479K (Fig. 2B) impaired precise measurement of 
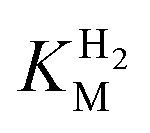
 at potentials more positive than −50 mV, therefore the measured 
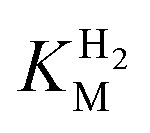
 at higher potentials is likely an underestimate, since even a 100% H_2_ atmosphere (corresponding to a concentration of approximately 0.76 mM at 20 °C) is below 4 × 
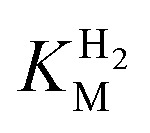
. Measurements of 
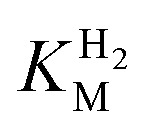
 were also carried out at 20 °C for R479K (Fig. S4†) due to a convex Eyring plot for H_2_ oxidation (see later).

Comparing the proton reduction currents at different H_2_ concentrations showed that R479K is less prone to product inhibition than Hyd-2 (Fig. 2 and S3†).^13,95^ Indeed, competitive inhibition by H_2_ calculated by poising the enzyme film at −660 mV while altering the H_2_ concentration (Fig. S5†) showed that R479K experiences a 
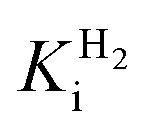
 of approximately 1.1 mM compared to 0.24 mM for native Hyd-2 (Table 1).^13^

#### Stability of the oxidised inactive resting state

**Fig. 4:**
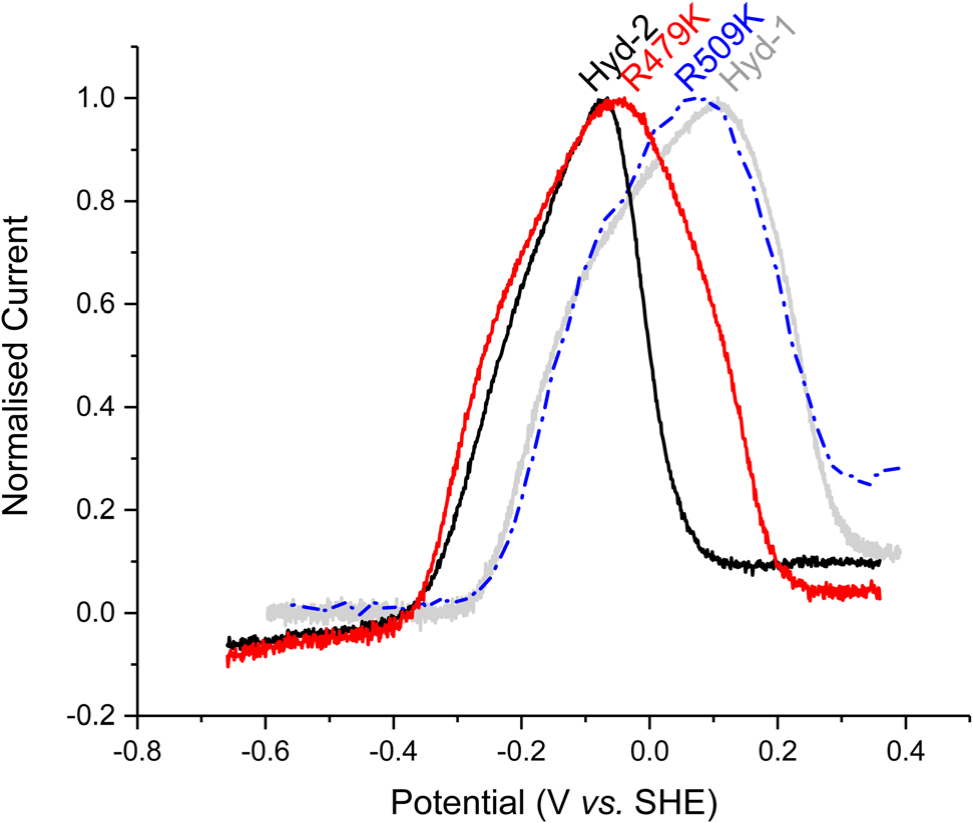
Determining the stability of oxidised inactive states for Hyd-1 and Hyd-2 enzymes. After full inactivation under anaerobic, oxidizing conditions, at high pH, the gas is switched to H_2_ (see Fig. S6†) buffer exchanged and the potential is slowly (0.1 mV s^−1^) lowered from the highly oxidizing potential (approx. +0.36 V). Other conditions: 30 °C, *ω* = 1000 rpm, pH 6.0, 100% H_2_ at 1000 sccm. The Hyd-2 and R509K data have been smoothed with the Savitzky–Golay method, 41 points per window using Origin software, due to lower signal-to-noise.

As shown in Fig. S6,† R479K is completely inactivated by poising at high potential under an argon atmosphere at high pH. The same procedure has previously been used for the inactivation of native Hyd-1, Hyd-2 and their variants.^12,27^ Generally, in the absence of O_2_, this inactivation represents the formation of Ni-B,^2,34^ though this is not always the case^34^ and should not be assumed. The relative ease of reductive reactivation can be gauged by slowly lowering the potential to reactivate the inactive state(s) in the presence of H_2_.^2^ The potential at which the protein reactivates under specific parameters is known as *E*_switch_ and is obtained from the minimum of the first derivative of the reverse scan (Fig. S7†), importantly complete anaerobic inactivation is essential, otherwise *E*_switch_ is not a meaningful parameter.^2,91^ This procedure has typically been used as a proxy measurement of the stability of Ni-B (Fig. 4).^28^ Although R479K begins to reactivate at a significantly higher potential than Hyd-2, measuring *E*_switch_ is complicated by the shape of the reactivation profile which shows a strong inflection point at approximately +0.160 V with a second weaker pseudo-inflection point at a potential more typical for *E*_switch_ of Hyd-2 found using the same procedure^12^—around 0 V (Fig. S7†). Any structural interpretation of *E*_switch_ is thus ambiguous in the case of R479K.

#### Inhibition of R479K

**Fig. 5:**
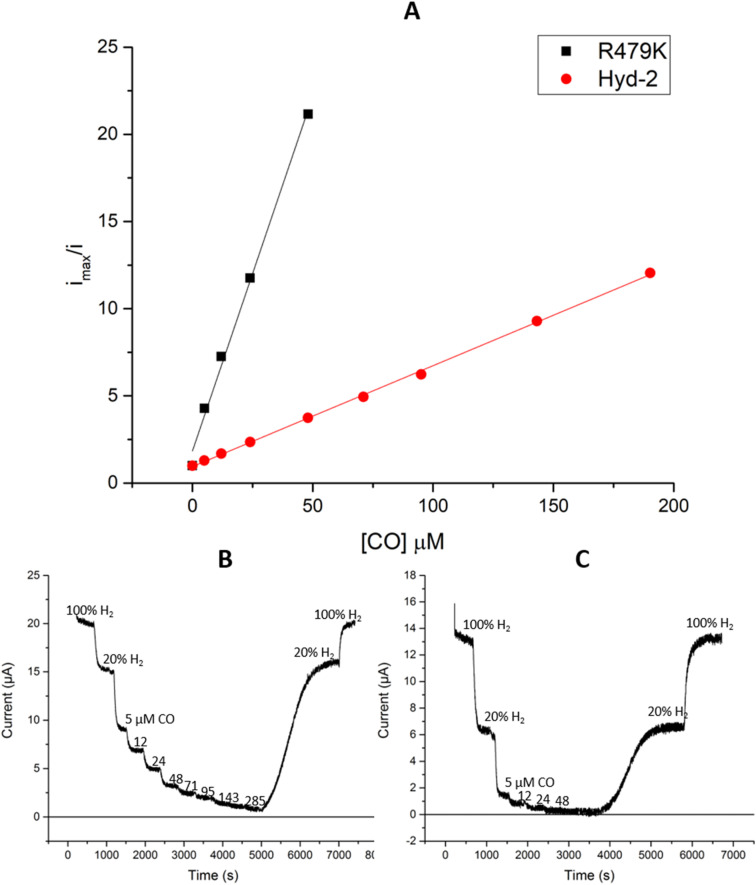
Calculation of the *K*^CO^_i_ (A) for Hyd-2 and R479K using the data from the profiles of inactivation in 20% H_2_ in varying concentrations of CO for (B) Hyd-2 and (C) R479K. Other conditions: potential −0.175 V, *ω* = 3000 rpm, 20 °C, pH 6.0, Ar carrier gas, total gas flow rate of 1000 sccm.

Carbon monoxide is a reversible inhibitor of H_2_ oxidation by Hyd-2.^13,91^ Although the R479K variant is more sensitive to CO than Hyd-2 for a given concentration of H_2_ (Fig. 5), the increased sensitivity to CO is almost entirely due to the difference in 
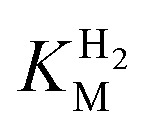
 between the native enzyme and R479K (Fig. 3A).^13,96^ Importantly, replacement of CO by H_2_ is rapid and reversible in both cases.

**Fig. 6:**
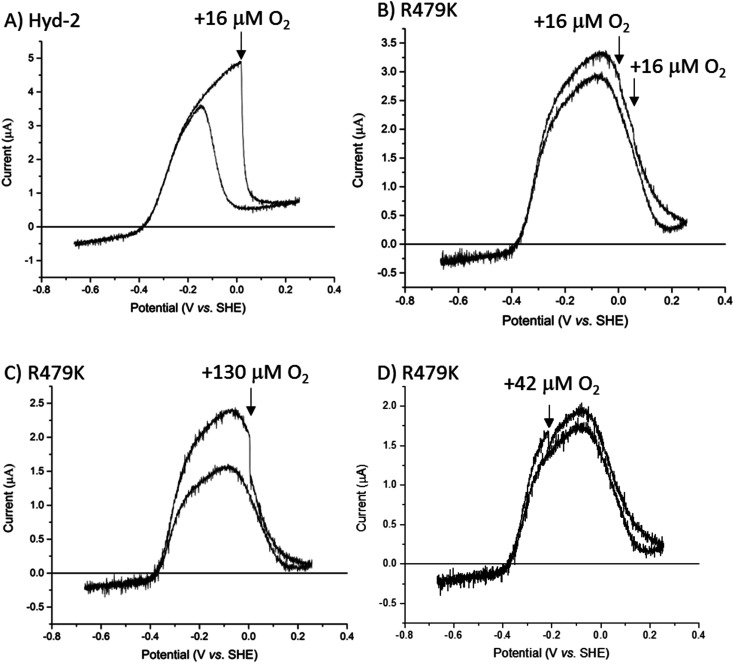
The response of Hyd-2 and R479K to a transient burst of O_2_ during H_2_ oxidation: under a constant flow of 100% H_2_ through the electrochemical cell headspace, the potential is taken from −0.67 V to +0.24 V at a rate of 0.5 mV s^−1^. At a specific potential on the sweep to high potential, O_2_-saturated buffer is injected (arrow) and immediately begins to be flushed from the system. By the time the potential sweep reaches +0.24 V and the scan direction is reversed, all O_2_ has been flushed from the system. (A) Response of Hyd-2 to an injection of O_2_ (16 µM initial concentration) at +0.05 V. (B) Response of R479K to a dual injection of O_2_ (to give 16 µM initial concentration) at 0 V and then immediately followed by another injection at +0.05 V. (C) Response of R479K to injection of O_2_ (130 µM) at 0 V and; (D) response of R479K to an injection of O_2_ (42 µM) at −0.215 V, *i.e.* before anaerobic inactivation dominates. Other conditions: 30 °C, *ω* = 3000 rpm, 100% H_2_, 0.5 mV s^−1^, total gas flow rate of 1000 sccm.

As an inhibitor of [NiFe]-hydrogenases, O_2_ is much more complicated than CO. The O_2_ sensitivity of Hyd-2 was measured first by injecting O_2_-saturated buffer into the electrochemical cell under a constant flow of H_2_. In this experiment, the transiently invading O_2_ begins to be removed almost immediately from the cell under the flow of H_2_.^96^ The immediate response to the burst of O_2_ and the duration of the effect of O_2_ on the current (H_2_ oxidation rate) depends on (a) the rate that O_2_ binds to the active site and (b) the rate at which the enzyme can deliver electrons and protons to the active site to ensure its complete reduction to harmless H_2_O.^9–11,97^ Normally, the final stage of a successful ‘rescue’ is reduction of the Ni(iii)-µ(OH^−^) product (Ni-B) and transport of the resulting water molecule away from the active site. As we found in earlier studies, Hyd-2 is highly sensitive to O_2_ attack:^13^ injection of O_2_ to give a short-lived burst of just 16 µM O_2_ almost fully inactivates the enzyme (Fig. 6A) and activity does not recover until the potential is lowered below 0 V during the reverse scan. In contrast, the cyclic voltammetry for R479K under 100% H_2_ was almost unaffected by two sequential injections of the same amount of O_2_-saturated buffer (Fig. 6B). Indeed, the voltammetry was only significantly disturbed by O_2_ at concentrations around 130 µM (Fig. 6C), and exposure to this level of O_2_ still only lowered the current by approximately half the level seen when delivering 16 µM O_2_ to R479K. However, R479K does not recover from the O_2_ attack until the applied potential is very reducing, and the current level is not fully recovered until the following scan (Fig. S8†). Exposure of R479K to O_2_ (42 µM) at a potential where there is no obvious anaerobic inactivation (−0.215 V) also showed minimal effect on H_2_ oxidation ability (Fig. 6D).

**Fig. 7:**
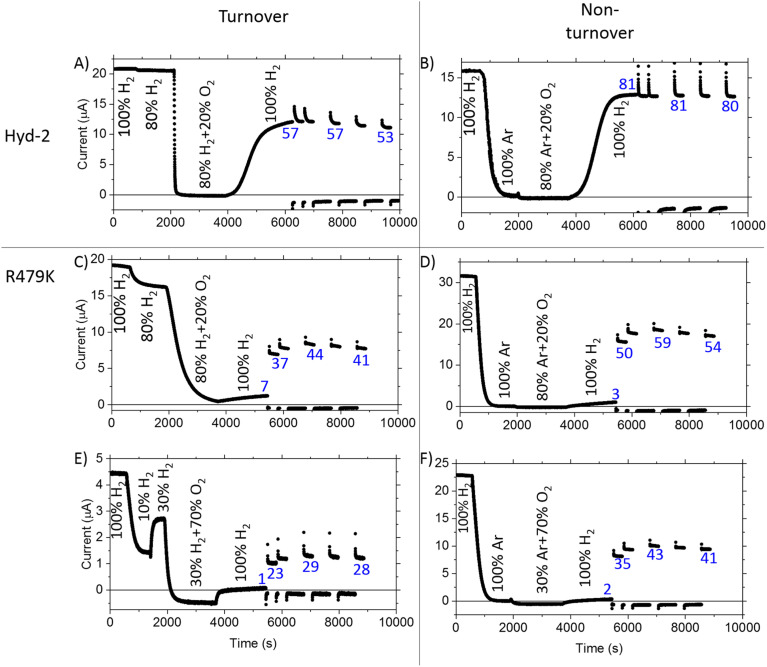
The response of Hyd-2 (A) and (B) and R479K (C)–(F) to prolonged exposure to O_2_ during turnover (left panels) and non-turnover (right panels) conditions: at a constant potential of −0.1 V, the headspace gas is changed from 100% H_2_ to a lower percentage of H_2_ – for O_2_ exposure during H_2_ turnover experiments this was either 80% H_2_ (A) and (C) or 30% H_2_ (E); for non-turnover experiments (right panels) this was 100% Ar (B), (D) and (F). Once this gas change had equilibrated (as judged by a stabilised current) O_2_ was introduced into the system at either 20% (A)–(D) or 70% (E) and (F). After approximately 30 minutes exposure to O_2_, the headspace gas was returned to 100% H_2_ at −0.1 V for at least 24 minutes. A series of potential steps were then employed, lowering the applied potential to −0.659 V for 1 or 10 min to reductively activate aerobically-generated species, and then back to −0.1 V to check for gain in H_2_ oxidation activity. The fractional recovery (%) of H_2_ oxidation activity after removal of O_2_ and following potential steps is denoted in blue (relative to the initial current obtained in 100% H_2_). Note: for Panel B data between approx. 0–1000 s were not auto-saved by the Nova software and have been manually extracted and digitised using Origin software.

To examine more closely the apparently ‘robust’ defence that R479K offers against transient exposure to O_2_, we tested the effects of prolonged exposure to O_2_ during turnover (H_2_ oxidation) and non-turnover conditions (Fig. 7) – the non-turnover experiments (argon/O_2_ in the absence of H_2_) more closely resembling conditions the enzymes would experience during isolation and purification. After measuring cyclic voltammograms to establish activity, experiments were performed in ‘chronoamperometric mode’ at a constant potential of −0.1 V, at which there is minimal anaerobic oxidative inactivation of both Hyd-2 and the R479K variant (Fig. 7), and minimal interference from background (direct) O_2_-reduction at the graphite electrode. The reference experiment against which to compare all others was conducted with Hyd-2 (Fig. 7A). Starting first under 100% H_2_ (approximately 0.8 mM at 20 °C) then 80% H_2_/20% Ar, an essentially instantaneous and complete extinction of activity was observed when 80% H_2_/20% O_2_ was introduced into the cell headspace, equivalent to 0.64 mM H_2_/0.29 mM O_2_. When the H_2_ level was restored to 100%, 57% of the activity was recovered spontaneously, no further increase being observed following periods when a highly reducing potential was applied (by stepping the applied potential to −0.659 V for 1 or 10 minutes and then back to −0.1 V to check for recovery of current). For the R479K variant (Fig. 7C), in the directly comparable experiment to that just described for Hyd-2 (Fig. 7A), the introduction of O_2_ caused the current to decrease slowly to almost zero over the course of 30 minutes, followed by only a slow partial spontaneous recovery when the atmosphere was restored to 100% H_2_, reaching 7% after 30 minutes at −0.1 V. Application of more reducing conditions resulted in 44% recovery. In view of the high 
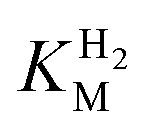
 value for R479K (Fig. 3A and Table 1) the experiment was repeated, starting with 100% H_2_, changing to 10% H_2_/90% Ar (80 µM H_2_), then 30% H_2_/70%Ar, before introducing 70% O_2_ (0.24 mM H_2_/1 mM O_2_) (Fig. 7E). Under this lower H_2_ level, the decrease in activity upon introducing O_2_ was faster than under 80% H_2_ (Fig. 7C) but still very slow compared with Hyd-2. No spontaneous recovery occurred when a 100% H_2_ atmosphere was restored (the slight increase in current is due to removal of O_2_ and thus removal of direct O_2_ reduction at the electrode surface^13^) and more reducing conditions resulted in up to 29% recovery.

In the non-turnover experiments (Fig. 7B, D and F), O_2_ (in Ar) was introduced to the cell headspace after first establishing activity then flushing all H_2_ from the system with 100% Ar. In all experiments the headspace was restored to 100% H_2_ after approximately 30 minutes of exposure to O_2_. At this point all traces showed a small current increase due to removal of background O_2_-reduction as O_2_ is flushed from the system. However, whereas the H_2_ oxidation activity of Hyd-2 increased gradually and spontaneously to 81% of the initial value after exposure to 20% O_2_, the R479K variant showed almost negligible recovery (2% when exposed to 70% O_2_ and 3% when exposed to 20% O_2_). Only after applying a much more reducing potential was R479K seen to recover significant H_2_ oxidation current (up to 59% and 43% after exposure to 20% and 70% O_2_, respectively). In contrast, Hyd-2 undergoes a high degree of recovery after switching to 100% H_2_ (81% in the experiment shown in Fig. 7B) which does not increase further after more reducing conditions are applied.

Immediate conclusions can be drawn from these experiments: reaction of O_2_ with Hyd-2 is fast and, regardless of whether or not H_2_ is also present, yields an inactive state that is readily activated upon reduction; in contrast, the reaction of O_2_ with R479K is slow, and largely escapes attention in transient exposure experiments monitored by cyclic voltammetry (Fig. 6), but yields an inactive state (or states) that requires much more forcing conditions for reactivation.

#### Activation enthalpies and entropies

Using transition state theory, the activation enthalpy Δ*H*^‡^ for a reaction is obtained from the gradient of an Eyring plot (log (rate/*T*) against 1/*T* where *T* is the absolute temperature). In electrochemical experiments, the direct relationship between rate and current (*i*) allows Δ*H*^‡^ to be determined under all different conditions of driving force Δ*G*, a relatively straightforward exercise when using cyclic voltammetry. The temperature dependence of current does not depend on stable enzyme coverage and therefore gives meaningful mechanistic insight, (particularly in the case of H_2_ oxidation) provided the H_2_ level is not so low that the current is controlled by its diffusion to the electrode surface. Cyclic voltammetric experiments were carried out over a 10–35 °C range (Fig. S9†), measuring the current at different potential values between −0.65 (H^+^ reducing) and +0.24 V (H_2_ oxidising) at a scan rate of 5 mV s^−1^ at pH 6 in 100% H_2_. In each case, plots of log (*i*/*T*) *vs.* 1/*T* were analysed.

**Fig. 8:**
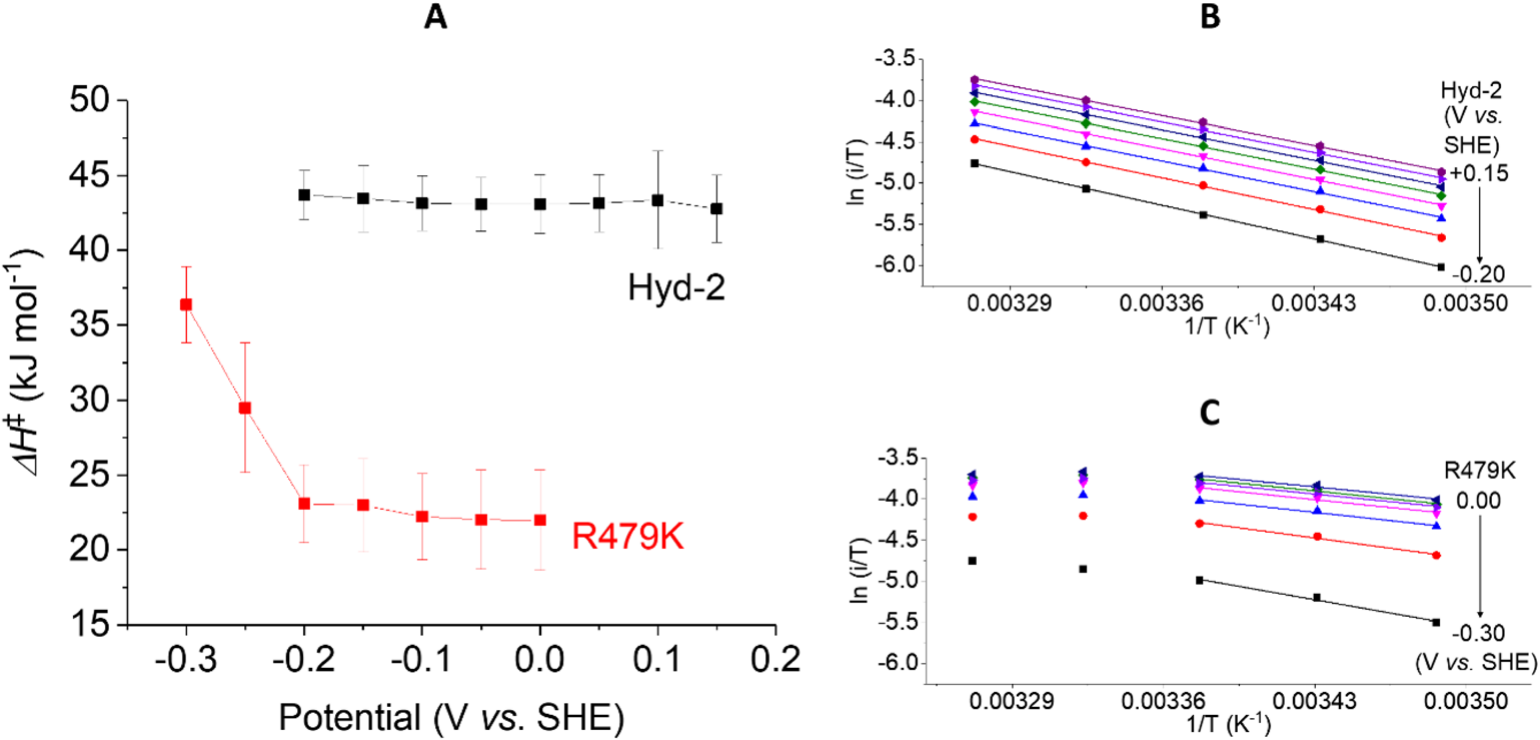
The potential dependence of activation enthalpy (Δ*H*^‡^) for H_2_ oxidation for (A) Hyd-2 and R479K, and examples of corresponding Eyring plots for Hyd-2 (B) and R479K (C). The value of Δ*H*^‡^ is calculated from the gradient of Eyring plots populated with data from scans at varying temperatures, returning to a control temperature every other scan to enable film loss correction. For R479K the Eyring plot is only linear below approximately 25 °C, thus only these data were fitted to a linear function. Error bars represent the standard deviation of three repeats. Other conditions: scan rate 5 mV s^−1^, *ω* = 1000 rpm, pH 6.0, 100% H_2_, total gas flow rate of 1000 sccm.

The Δ*H*^‡^ value obtained for H_2_ oxidation by Hyd-2 (ref. 12) at −0.2 V (Table 1 and Fig. 8B) agrees well with data measured previously.^98^ In contrast, the R479K variant showed a convex deviation of the Eyring plot (Fig. 8C) with the slope becoming zero or slightly negative at the most oxidising potential (0 V *vs.* SHE) and the highest temperatures. Based on data obtained below approx. 25 °C, where the gradient is steepest, a value of Δ*H*^‡^ = 23 kJ mol^−1^ was estimated for H_2_ oxidation by R479K at −0.20 V. Notwithstanding the likelihood that this value is an underestimate, it still represents a very large decrease (up to 21 kJ mol^−1^) compared to native Hyd-2 (Δ*H*^‡^ = 43.7 kJ mol^−1^). This result was consistent across the potential range −0.2 to 0 V (Fig. 8A).

**Fig. 9:**
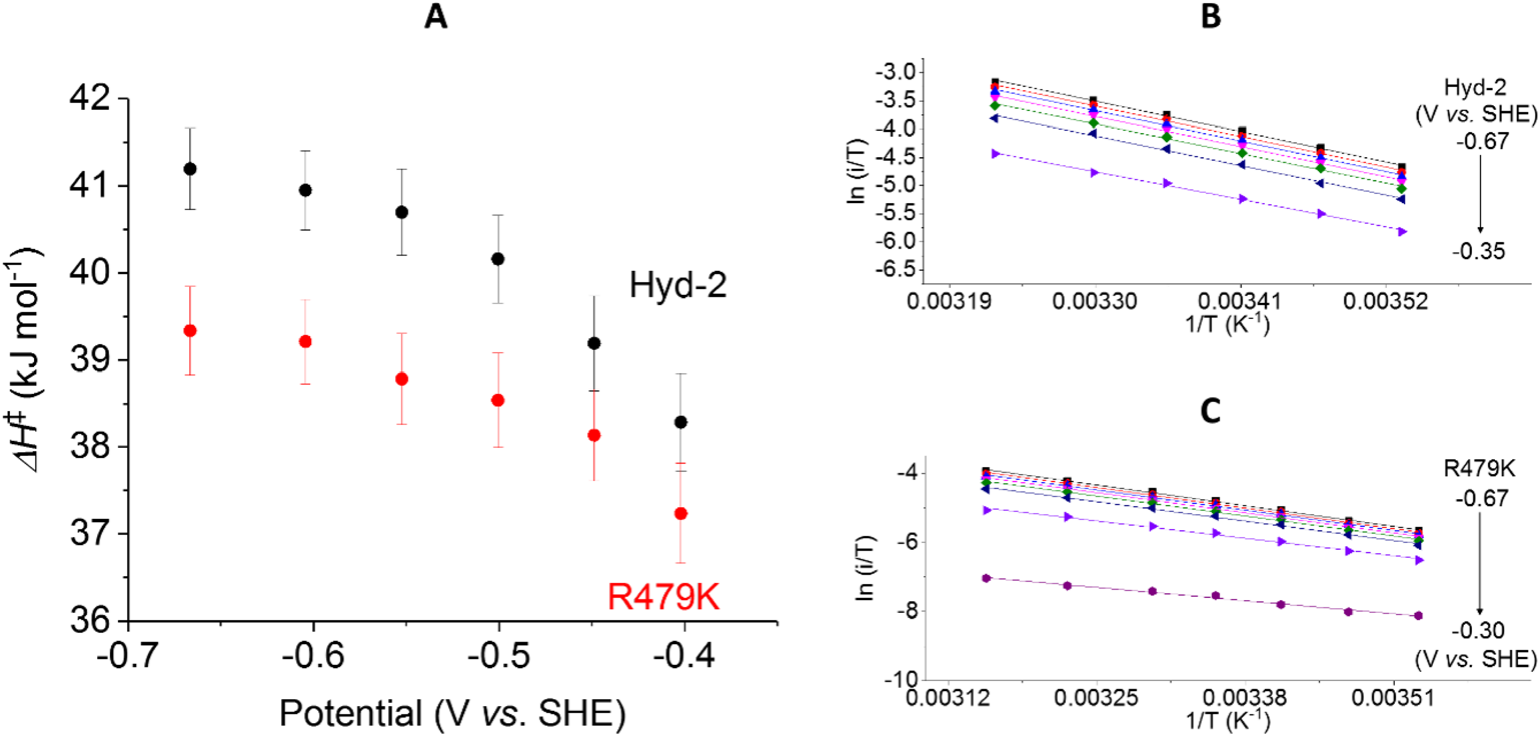
The potential dependence of activation enthalpy (Δ*H*^‡^) for H^+^ reduction for Hyd-2 and R479K (A). The Δ*H*^‡^, for Hyd-2 (B) and R479K (C), is calculated with Eyring plots populated with data from scans at varying temperatures with the film brought back to the control temperature every other scan to correct for film loss. Error bars represent the standard deviation of three repeats. Other conditions: 5 mV s^−1^, *ω* = 1000 rpm, pH 6.0, 100% Ar, total gas flow rate of 1000 sccm.

The activation enthalpy for H^+^ reduction was also measured over a 10–45 °C temperature range (Table 1 and Fig. 9). Due to the effect of product (H_2_) inhibition^12,13^ data were obtained under 100% Ar at pH 6 by cyclic voltammetry between −0.65 and −0.17 V at 5 mV s^−1^. The Eyring plot in the H^+^ reduction potential window was linear across the entire temperature range used for Hyd-2 and R479K (Fig. 9B and C). The Δ*H*^‡^ value for R479K at −0.55 V is lower than that of Hyd-2 by approximately 1.5 kJ mol^−1^ and both enzymes show a slight potential dependence, the activation enthalpy increasing as the potential is lowered (Fig. 9A).

In addition to extracting values for Δ*H*^‡^, Eyring plot data can be used to calculate the activation entropy of the reaction (Δ*S*^‡^). In PFE, because the enzyme coverage of the electrode used in the temperature dependence experiments is unknown, the intercept of the *y* axis cannot be used to calculate Δ*S*^‡^, instead we rely on the value of Δ*H*^‡^ and the difference in steady-state rates measured in solution (Table 1) to estimate the difference in activation entropy for the two enzymes (ΔΔ*S*^‡^) at a given potential (see ESI†).

Using the steady-state H_2_ oxidation rates in addition to the value of Δ*H*^‡^ calculated at −0.2 V, (to achieve a potential comparable with the benzyl viologen electron acceptor used in the assay, Table 1) the difference in activation entropy between Hyd-2 and R479K, ΔΔ*S*^‡^, is estimated to be −101 to −87 J K^−1^ mol^−1^, which is approximately 50 J K^−1^ mol^−1^ more negative than the additional entropic barrier calculated by the same method for Hyd-1 *vs.* R509K.^28^ The activation enthalpy for H^+^ reduction by Hyd-2 is significantly lower in 100% Ar than when measured in 100% H_2_,^98^ which probably reflects a contribution from product inhibition. The difference in activation enthalpies for Hyd-2 and R479K for H^+^ reduction are not as dramatic as for H_2_ oxidation, but the reaction is still entropy-controlled with a ΔΔ*S*^‡^ estimated to be −49 to −36 J K^−1^ mol^−1^.

#### Kinetic isotope effect

The large decrease in Δ*H*^‡^ for H_2_ oxidation observed when arginine-479 is replaced by lysine coincides with the results obtained for Hyd-1 when the analogous arginine-509 is replaced by lysine.^28^ For a large rate retardation to be associated with a large decrease in energy barrier is unusual and this matter will be discussed later. To test the possibility that H(D) tunnelling might have become rate limiting for R479K, experiments were conducted to measure and compare the kinetic isotope effect, KIE, for the R479K variant and Hyd-2 when H_2_ and D_2_ were interchanged at 10 °C, 20 °C and 37 °C (*i.e.*, spanning the convex Eyring plot for R479K, Fig. 8C) and as a function of potential (Fig. S10†). The electrode potential was stepped between −0.3, −0.2 or −0.1 V in an atmosphere of 100% H_2_ and the current was allowed to stabilise in each case. The headspace gas was then swapped for 100% D_2_ (BOC, 100% purity) and the same potential steps applied. The whole process was repeated several times to allow for film loss correction and the average current value was determined for the last approx. 1 minute of data points collected at each potential step. The KIE was determined from the current ratio measured for H_2_*vs.* D_2_. Generally, at each temperature the KIE increased with potential, and at each potential the KIE decreased with increasing temperature (Table S2†). The KIE values ranged between 1–1.25 depending on potential and temperature.

### 3.4 Structures of Hyd-2 R479K

**Fig. 10:**
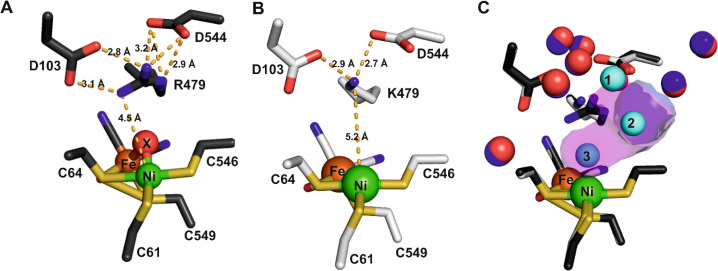
(A) The active site of as-isolated Hyd-2 showing the extensive hydrogen bond network stabilising the position of the guanidinium headgroup of R479 as dashed lines. Interatomic distances (Å) are also indicated. (B) The active site architecture of the R479K Hyd-2 variant indicating the position of the pendant nitrogen atom of K479 relative to the catalytic Ni atom. Hydrogen bonds stabilising the conformation of the side chain of K479 are also indicated. (C) An overlay of the active sites of native Hyd-2 (black) and R479K (white) indicating the almost identical architecture of the active sites, with the exception of residue 479. Crystallographically ordered water molecules are also shown (native = blue and R479K = red) highlighting their positional conservation. The R479K variant contains three additional water molecules (cyan) relative to the native protein. The magenta surface indicates the extension to the gas channel resulting from the unusual conformation of K479. The gas channel in Hyd-2 is also shown as a purple surface for comparison.

**Fig. 11:**
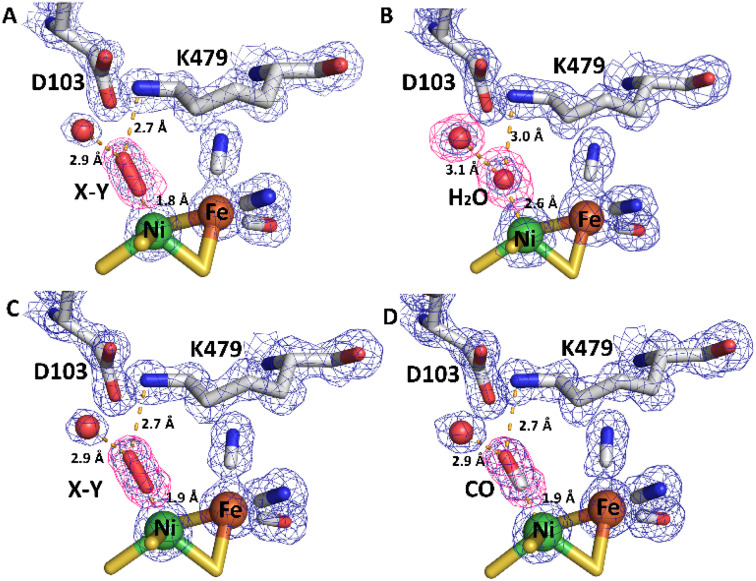
Electron density (2Fo-Fc and omit) maps and resultant structural models of the active site of R479K after different gas treatment regimes. (A) The as-isolated enzyme showing additional density adjacent to the Ni atom represented as diatomic ligand X–Y. (B) The reduced active site after exposure to an atmosphere of 100% H_2_ for 20 hours displays near spherical density (in 2Fo-Fc and omit maps) next to the Ni atom, suggesting a water molecule has replaced the diatomic ligand. For comparison, an omit map has been included for the water molecule next to D103, which also shows near spherical density. (C) After exposure of the reduced enzyme to pure oxygen, showing that electron density for diatomic ligand X–Y reappears in the active site. (D) Exposure of the reduced enzyme to CO indicates binding of CO to the Ni atom of the active site. The 2Fo-Fc electron density maps (blue mesh) are contoured at 1.5 r.m.s.d corresponding to an absolute value of 0.6 electrons per Å^3^. Omit maps calculated in the absence of Ni-coordinating ligand (pink mesh) are contoured at 5 r.m.s.d., an absolute value of 0.66 electrons per Å^3^. All hydrogen-bonds stabilising the bound ligands are shown as dashed lines and their lengths indicated. This figure is reproduced in the ESI (Fig. S15†) without the omit density to highlight the contiguous 2Fo-Fc density linking the Ni atom and bound ligand. Temperature factors for the components of each active site are shown in Table S5.†

The structure of the aerobically purified ‘as isolated’ R479K variant (pdb accession 6SYO, Table S3†) was solved to a resolution of 1.25 Å (Fig. 10B and 11A). The active site is fully formed with minimal change in the position of sidechains relative to the native enzyme, except residue K479 (Fig. 10C), suggesting any changes in kinetic parameters are a consequence of replacing the guanidinium group with a primary amine. The K479 side chain adopts an atypical conformation by rotation about the Cγ–Cδ bond, allowing Nζ to contribute in the hydrogen bond network between the Oδ1 atom of the D103 and Oδ2 atom of D544. The rotated conformation increases both the distance between the head group of residue 479 and the Ni-atom (5.2 Å) and the diameter of the terminus of the hydrophobic channel immediately adjacent to the active site (2.8 Å) relative to the native enzyme (with values of 4.5 Å and 2.2 Å respectively).^12^

#### The origin of the unusual conformation of K479

The position of the sidechain of residue K479 is invariant between gas treatments and results from an increased volume of the active site cavity of Hyd-2 (∼3.0 Å^3^) relative to Hyd-1 (∼2.0 Å^3^).^27^ The active-site cavity volume is defined as the space between the [NiFe] cofactor and the ‘canopy’ – residues 103, 479 and 544. The increased volume arises from rotation of helix 21, spanning residues 534–544, by 2.5° about its C-terminus, which shifts the side chain of D544 away from that of residue 479. Helix 4, spanning residues 94–113, is also rotated by 2.5° about its C-terminus relative to its position in Hyd-2, increasing the distance between the carboxylate group of D103 and residue 479. These two shifts likely result from a loss of hydrogen bonds to the guanidinium head group of R479 in the native enzyme, a residue important for maintaining the rigidity of the protein near the active site,^99^ with a cumulative effect of expanding the active site. This movement necessitates that K479 adopt a rarely observed conformation to form a single hydrogen bond with each of the carboxylate groups of D103 and D544, (see Fig. S11† for comparison of hydrogen bonding in the active sites of Hyd-1 and Hyd-2). The hydrogen bonding network in R479K is more limited than that observed for Hyd-1 R509K, where the amine group forms two hydrogen bonds to equivalent residues D118 and D574. Modelling the side chain of K479 in the conformation adopted in the more sterically constrained active site of Hyd-1 places the Nζ atom at a distance 3.5–4.1 Å from the carboxylate groups of D103 or D574 eliminating any stabilization afforded by hydrogen bonding. A further consequence of the unusual conformation observed for K479 is an extension of the gas access channel towards the [NiFe] cofactor, forming a small “pocket” next to the Ni-atom (Fig. 10C), which contains an additional peak of electron density unseen in Hyd-2.

#### Modelling the additional electron density

From the size and shape of the additional electron density found in as-isolated R479K, but absent in as-isolated Hyd-2, it could be concluded that a diatomic ligand is coordinated ‘end-on’ to the Ni (Fig. 11A). Henceforth we will refer to this ligand as “X–Y”, X being proximal and Y distal with respect to the Ni atom. In contrast to as-isolated Hyd-2,^12^ (and, indeed, other as-isolated, standard [NiFe]-hydrogenases^100,101^), there was no evidence for a single bridging oxygen atom or any indication that oxygenation of a coordinating cysteine-S atom had occurred. The as-isolated structure also showed an unexpectedly short Ni–Fe separation of 2.64 Å, significantly lower than the value of 2.95 Å typically observed in as-isolated (Ni-A and Ni-B) structures of [NiFe]-hydrogenases and more reminiscent of distances seen in structures of H_2_-reduced protein^28^ and more recently an F420-reducing hydrogenase obtained in the Ni^II^-SI state (the most oxidized state in the catalytic cycle).^102^

Exposure of the protein sample to H_2_ flow for 9 hours prior to crystallization resulted in crystals in which the occupancy of the diatomic species had decreased to approximately 40% of that in the as-isolated structure. Increasing the duration of H_2_ exposure to 20 hours appeared to remove the bound diatomic ligand, which was replaced by electron density assigned to a coordinated water molecule (Fig. 11B, pdb accession 6SZD, Table S3†). In a more aggressive procedure, a solution of R479K at 5 mg mL^−1^ was diluted 2 : 1 with a 1 mM solution of electrochemically reduced methyl viologen then purged with 100% H_2_ for 40 hours. Steady-state solution assays showed that R479K activated in this manner displayed approximately 5-fold higher activity, however, inspection of electron density maps suggested this method was no more effective at removing the bound diatomic ligand than simply exposing to H_2_ for 20 hours. The increase in steady-state rate upon additional electrochemical reduction is likely due to activation of other slow-to-reactivate states that are not crystallographically resolved.

The orientation and geometries of the metal atoms and amino acids in the reduced structures were unchanged from those of the as-isolated structure (RMSD 0.04 Å), with the most significant difference being removal of the diatomic species. Re-exposure of the reduced sample to pure O_2_ for 1 hour resulted in large increases in electron density for the bound diatomic species, the occupancy level returning to approximately 80% (pdb accession 6SYX, Fig. 11C and Table S3†). The X–Y bond length is approximately 1.2 Å. The X-atom is located 1.85 Å from the Ni atom and the Y-atom is positioned so that it can form hydrogen bonds to the side chain Nζ atom of K479 and a crystallographically ordered water molecule. The resulting stable complex has a Ni–X–Y angle of 163°. A structurally identical complex was also formed upon anaerobic oxidation with DCIP, but with the diatomic ligand showing full occupancy, in agreement with IR-spectroscopy analysis (see below).

All R479K variant structures contain two additional water molecules in the active site (labelled 1 and 2 in Fig. 10C) as a result of replacing the bulky guanidinium head group of residue 479 with a smaller amine group. One of the new water molecules is positioned above the Nζ, atom, similar to that observed previously in the as-isolated structure of Hyd-1 R509K (compare H_2_O ‘1’ in Fig. 10C with Fig. S14†).^27^ Ordered water molecule ‘2’ (Fig. 10C) is positioned 4.2 Å above the sulphur of a terminal cysteine ligand (C546) proposed to be involved in the mechanism of H_2_ activation,^16,26,32,33,103^ and is 3.6 Å away from the Nζ atom of K479: this water molecule is also observed in Hyd-1 R509K, albeit with a lower occupancy (Fig. S14†). Additionally, H_2_-reduced R479K contains a third water molecule (H_2_O ‘3’ in Fig. 10C) that appears to be weakly coordinating (∼2.6 Å) the Ni atom alone, where the distance to the Fe atom is too long (4.0 Å) for it to be considered as a bridging ligand.

#### Structure of CO-inhibited form

The diatomic electron density occupies an area similar in position to exogenous CO seen previously in the [NiFe]-hydrogenase from *Desulfovibrio vulgaris* Myazaki F:^104,105^ Therefore to make a direct comparison, a crystal structure of the CO-inhibited form of R479K was obtained after treating H_2_-reduced enzyme with 100% CO for 20 minutes. Differences in the electron density adjacent to the Ni atom suggested that CO occupies the pocket next to the Ni (pdb accession 6SZK, Fig. 11D and Table S3†), although mixed occupancy remains possible. Significantly, the electron density is distributed asymmetrically across the two atoms in the CO molecule with more residing on the O atom, similar to the distribution observed for the permanent CO ligand to the Fe atom (Fig. S12 and Table S4†). The exogenous CO molecule binds to Ni *via* the carbon atom at a distance of 1.85 Å with a Ni–C–O bond angle of 169°. As described above, CO behaves very differently to O_2_ in kinetic experiments, where it functions instead as a reversible inhibitor.

### 3.5 Spectroscopic analysis

#### EPR spectroscopy

**Fig. 12:**
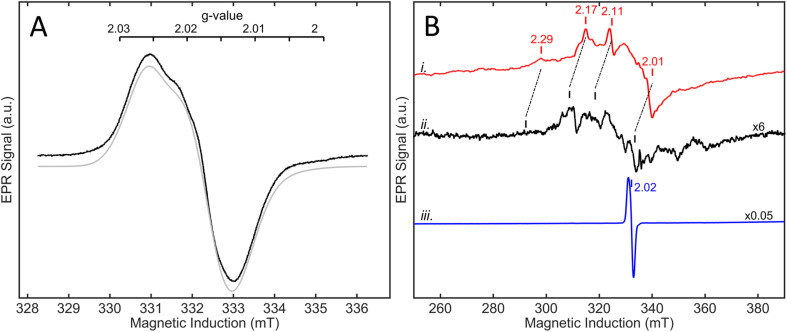
EPR spectra of as-isolated R479K at (A) 10 K at 90 µM protein concentration, and (B) comparing 400 µM, trace (i), and 90 µM for trace (ii) & (iii). The temperature of traces (i) & (ii), was 80 K, while (iii) was measured at 10 K. All traces are multiplied by temperature and scaling factors. Trace (i), measured for 24 hours, was of a 1.2 mm ID tube in a MS2 resonator, while traces (ii) & (iii) were of 2.8 mm ID tubes in the SHQE resonator. Microwave frequencies were 9.573, 9.387, 9.384 GHz respectively; modulation amplitude 0.8, 1, 0.1 mT, respectively; and microwave power 0.05, 10, and 0.05 mW, respectively, the third values being applied also to panel A.

The X-band EPR spectrum of concentrated (90–400 µM) as-isolated R479K measured at 70 K was distinguished by the almost total absence of signals due to Ni (Fig. 12). Instead of the typical spectrum observed for as-isolated Hyd-2 which consists of signals due to Ni-A and Ni-B,^12,13^ only very weak signals were observed, with *g*-values 2.19, 2.11 and 2.01. Although these *g*-values most closely resemble those associated with Ni–C (2.193, 2.137, 2.01)^12^ which is a reduced form, the fact that the spectrum recorded at 10 K was dominated by the signal of the oxidized medial FeS cluster ([3Fe–4S]^+^ (*S* = ½), *g*_sim_ = [2.013, 2.017, 2.027], *σ*_sim_ = 0.003) showed that the enzyme should be in an oxidized state. The results also suggested strongly that as-isolated R479K does not possess an unpaired electron at the active site.

#### Infrared spectroscopy

**Fig. 13:**
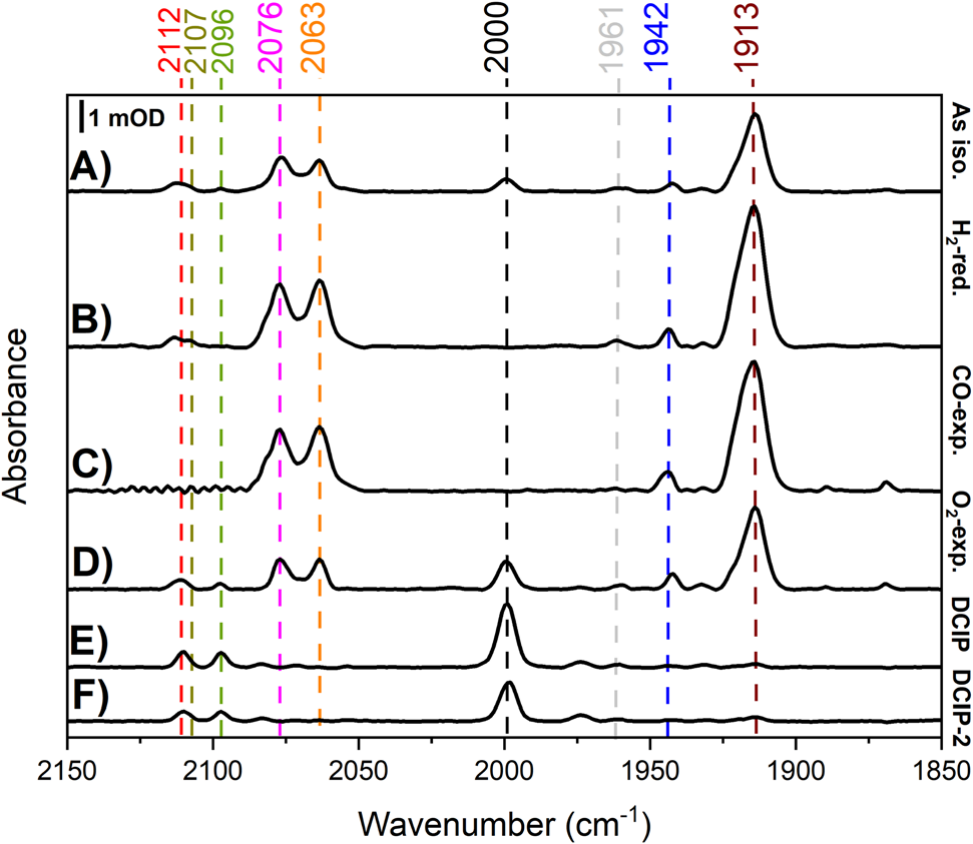
Solution-based transmission IR spectra of R479K following various gas treatments. IR spectra are presented from ∼1 mM samples of R479K prepared as (A) as-isolated, (B) H_2_-reduced, (C) H_2_-reduced, oxidised with mediators then exposed to CO, (D) H_2_-reduced then exposed to O_2_, (E) H_2_-reduced then oxidised with DCIP under strict anaerobic conditions, and (F) H_2_-reduced, left overnight and then oxidised with DCIP prepared the previous day to avoid traces of O_2_. All samples were prepared and the spectra collected at room temperature in an anaerobic glovebox. All spectra are shown on the same scale (bar), and the *v*_CO_ and *v*_CN_ bands are highlighted by dashed lines. Typical IR bands for native Hyd-2 and the oxygen sensitive hydrogenase from *D. vulgaris* Myazaki F are listed in Table S6.†

Reactions of R479K with different gases were probed by transmission-based IR spectroscopy of concentrated solutions of the variant enzyme under different gas treatment regimens (Fig. 13). The IR spectrum of as-isolated R479K (Fig. 13A) is dominated by a broad *v*_CO_ band centred at 1913 cm^−1^ (with a shoulder at 1915/1920 cm^−1^) likely linked to the pair of *ν*_CN_ bands at 2076 and 2063 cm^−1^. Additional species include a state with an unusually high wavenumber *v*_CO_ (2000 cm^−1^) and an accompanying pair of high wavenumber *v*_CN_ at 2096 and 2110 cm^−1^. Since the hydrogen-bonding network of the active site of R479K has been disrupted (Fig. 10), the electronic structure of the active site will have been altered, which will be reflected in the *ν*_CO_ and *ν*_CN_ positions of active site states – none of the infrared spectroscopically assigned states of Hyd-2 are observed in R479K. Infrared spectra measured for as-isolated Hyd-2, however, are dominated by a broad peak centred at 1957 cm^−1^ corresponding to a mixture of Ni_a_-A and Ni_a_-B. To obtain a spectrum of reduced R479K, a dilute enzyme solution (∼100 µM) was reductively activated under humidified 100% H_2_ for 24 hours in an anaerobic glovebox and subsequently concentrated to 1 mM under anaerobic conditions. The resulting spectrum of the H_2_-reduced sample (Fig. 13B) shows complete loss of the *v*_CO_ band at 2000 cm^−1^, with the spectrum still dominated by v_CO_ bands around 1913 cm^−1^. This observation contrasts with that made for H_2_-reduced Hyd-2 which shows a spectrum dominated by Ni_a_-C (*v*_CO_ 1966 cm^−1^) and Ni_a_-SI (*v*_CO_ 1945 cm^−1^).

An experiment was then conducted to examine the reaction of R479K with CO. For Hyd-2, a CO inhibited state, NiS–CO is formed when the active Ni_a_-SI state (*v*_CO_ 1945 cm^−1^) reacts with CO; NiS–CO exhibits a *v*_CO_ for the endogenous carbonyl ligand at 1944 cm^−1^, and a new band for the exogenous, inhibitory CO on Ni(ii) at 2054 cm^−1^. The solution potential of the reduced R479K sample was raised by addition of a cocktail of redox mediators (final concentration 0.5 mM in each) of anthraquinone 2 sulfonate (−151 mV), indigo carmine (−29 mV), and phenazine methosulfonate (+139 mV).^74^ For Hyd-2 this condition favours conversion of reduced states to Ni_a_-SI; however for R479K no significant change in the spectrum was observed. The sample was then flushed with 100% humidified CO gas for 1 hour and left under a slight overpressure of 100% CO overnight (*ca.* 18 hours). Again, no significant changes were observed in the spectra for R479K (Fig. 13C). The fact that NiS–CO is not obviously formed with this variant is unsurprising given that it also does not exhibit a recognisable Ni_a_-SI state.

To explore the reactivity of R479K with O_2_, an aliquot of the reduced variant was treated with a flow of humidified 100% O_2_ for 2 hours, and then left under a slight overpressure of O_2_ for an additional hour. The IR spectrum of the O_2_-exposed protein looked largely similar to the as-isolated sample, again showing a species with high wavenumber *v*_CO_ at 2000 cm^−1^ together with unusually high wavenumber *v*_CN_ bands (compare Fig. 13A and D). The fact that the ‘high wavenumber species’ appearing at 2000 cm^−1^ is lost upon H_2_ reduction, and reformed upon exposure to O_2_ demonstrate that this species undergoes reversible redox chemistry at the active site.

To ascertain whether the high-wavenumber species forms only in the presence of O_2_, the variant was exposed to oxidant under anaerobic conditions: an aliquot of H_2_-activated R479K (∼1 mM) was treated with an excess (2 mM final concentration) of 2,6-dichlorophenolindophenol (DCIP, 2e^−^ donor/acceptor, *E*_m_ = +317 mV *vs.* SHE^74^) prepared in an anaerobic buffer (20 mM Tris, pH 7.2, 150 mM NaCl). The resulting IR spectrum shows full conversion into the high-wavenumber species having bands at *v*_CO_ 2000 cm^−1^ and *v*_CN_ at 2096 and 2112 cm^−1^ (Fig. 13E). To exclude the possibility of trace O_2_ contamination, the procedure was repeated using a DCIP solution that was left inside an anaerobic glovebox overnight. As shown in Fig. 13F, the spectrum is reproduced, confirming that formation of the high wavenumber, oxidised species appears independently of any intentional exposure to O_2_. The fact that no corresponding set of high wavenumber IR bands is observed for the CO-exposed sample, and that there is no additional band which could be attributed to exogenous CO, indicates that it is not a CO-inhibited state. The cyanide region was also explored for additional CN bands in the anaerobically oxidized state and the raw data are shown in Fig. S16A.† Above 2112 cm^−1^ no bands are detected even in the second derivative spectrum (Fig. S16B†). Furthermore, the spectrum of the DCIP-oxidised sample shows the high wavenumber species as a single, majority form of the active site, eliminating the possibility that this new species arises from binding of CO released from enzyme molecules with degraded active site, as is observed for FeFe hydrogenases. To confirm this point, we recorded IR spectra on single crystals of R479K at two reducing potentials, −700 and −150 mV with and without CO, and saw no evidence for the species with *ν*_CO_ at 2000 cm^−1^ in these spectra. Additionally, we confirmed that anaerobic oxidation to form the high wavenumber species could be reproduced in crystals to a majority species with *ν*_CO_ at 2000 cm^−1^ which was insensitive to treatment with CO (Fig. S17 and ESI discussion†). Oxidative and reductive redox titrations performed on single crystals of R479K at pH 6.0 in the potential range −600 to +400 mV showed that the high wavenumber species is reversibly formed at potentials above +150 mV and is the majority (pure) state at +400 mV (Fig. S18 and ESI discussion†).

### 3.6 Computational analysis

#### Calculated models

Multiple structural models were considered for the NiFe cofactor in different redox states of the Ni–Fe cofactor and with different X–Y ligands bound to the Ni atom (see ESI for details†). QM/MM calculations were performed to account for the protein environment surrounding the cofactor.

#### Comparison to IR data

Vibrational frequencies were calculated for all structural models (Fig. S19†). Vibrational frequency calculations for an optimized Ni(ii)–Fe(ii) model with a Ni-bound H_2_O ligand give Fe–CN^−^ vibrational frequencies of 2094 and 2073 cm^−1^ and a Fe–CO frequency of 1914 cm^−1^. This model is consistent with the IR data for “H_2_-reduced” showing dominant IR peaks at 2076, 2063 and 1913 cm^−1^. The slight overestimation of the CN^−^ modes seen may be due to the charged CN groups not being well described by the non-polarizable QM/MM model or alternatively due to the DFT description of the Fe–CN chemical bonds. The Ni(ii)–Fe(ii) with Ni-bound H_2_O seems a plausible model for the “reduced” state, with the X-ray structure showing a single light (O) atom bound to Ni.

The experimental shift (33–36 cm^−1^) of the 2076/2063 cm^−1^ CN “H_2_-reduced” bands to 2112/2096 cm^−1^ upon DCIP addition is consistent with oxidation of the cofactor. The simplest computational model considered which explains the experimental data, contains an oxidized Ni(iii)–Fe(ii) species without any exogenous ligand bound. This model gives 2122/2111 cm^−1^ for the CN bands, *i.e.* a predicted shift of 28–38 cm^−1^ compared to the H_2_O-bound Ni(ii)–Fe(ii) model. Analysis of the spin density clearly reveals Ni-based oxidation (with only a small spin population on the Fe ion) which nonetheless results in this shift of the Fe–CN modes. The experimental 2000 cm^−1^ peak in the experimental DCIP data could be explained by a strong shift (87 cm^−1^) of the Fe-bound CO band from 1913 cm^−1^ (“H_2_-red”) to 2000 cm^−1^ (“DCIP”) due to some charge-transfer away from the Fe site. The calculated Ni(iii)–Fe(ii) model without a ligand on Ni, gives a calculated CO mode of 1967 cm^−1^ compared to the 1914 cm^−1^ mode in the H_2_O-bound Ni(ii)–Fe(ii) model, amounting to a shift of 53 cm^−1^ compared to the experimental shift of 87 cm^−1^. While this comparison suggests that the experimental 2000 cm^−1^ peak is plausibly assigned to a strongly shifted Fe–CO mode upon Ni oxidation, the calculations of the Ni(iii)–Fe(ii) model without a ligand do not fully capture this effect.

We next considered Ni(iii)–Fe(ii) models with a terminal X–Y ligand bound to Ni. These models gave overall similar Fe–CN mode energies and Fe–CO modes as models without the ligand, suggesting the Fe–CN/CO modes to be primarily sensitive to the overall redox state and not Ni coordination. Overall, a Ni(iii)–Fe(ii) model with a relatively innocent ligand emerges as a plausible model for the DCIP-oxidized state.

We also considered the possibility of additional oxidation to give either a Ni(iv)–Fe(ii) or a Ni(iii)–Fe(iii) species which could conceivably be responsible for a shift of an Fe-bound CO peak to 2000 cm^−1^. A recent structure of the oxygen-tolerant [NiFe] hydrogenase from *H. thermoluteolus*, reveals a glutamate coordinating to the Ni.^106^ In this form of the enzyme, the active site which displays a band at 1993 cm^−1^ was assigned as a Ni(iv)–Fe(ii) species,^107^ although it has since been proposed that an antiferromagnetically coupled Ni(iii)–Fe(iii) system is more plausible.^108^ However, calculations starting from the X-ray structures in this work did not give electron structures consistent with either oxidation to Ni(iv) or Fe(iii). Instead, all attempts to force the calculation (see ESI†) to give a local Fe(iii) oxidation state resulted instead in S-centred oxidation occurring over one or more thiolates, which is highly unlikely to be a stable species.

#### Comparison of calculated models with X-ray crystal structures

Multiple possibilities were explored computationally for the unknown diatomic ligand seen in the as-isolated X-ray structure. The ligands considered were: O_2_, CN^−^, CO, N_2_ and NO and were primarily calculated with the cofactor in either Ni(ii)–Fe(ii) or Ni(iii)–Fe(ii) redox states (the Fe ion remained Fe(ii) throughout according to spin population analysis). Further oxidized species were also explored but resulted in one-electron thiolate oxidation and such models were dismissed as unlikely. Considerable effort was devoted to exploring O_2_ as a possible ligand in the calculations (Fig. S21–S24†). A model with the cofactor in the Ni(ii)–Fe(ii) redox state resulted in spontaneous Ni(iii)-superoxide formation upon O_2_ addition. All models considered (varying charge, spin state, DFT method, QM-region *etc.*) gave rise to calculated Ni–O–O angles of ∼125° in disagreement with the ∼160° angle seen in X-ray crystal structure of the as-isolated state. Other ligands were compared in Fig. S25† for the Ni–X–X angle. Models featuring CN^−^, CO and N_2_ ligands give near-linear Ni–X–X angles (168°–177°), in much better agreement with the X-ray structure. Relevant data for oxidised models are displayed in Fig. S26.†

## 4. Discussion

The dimensions of the substrate-access channel^28^ are unaffected by the mutation apart from a widening where it meets the active site, and CO inhibition (hence diffusion) is rapid, suggesting substrate availability at the active site is not affected significantly. However, crystal structures are static representations and do not account for transient flexing of the molecule, which could be altered in R479K. Comparison with the structure of Hyd-1 R509K shows the sidechain of K509 to be highly flexible relative to other canopy residues,^27^ and in the absence of the X–Y ligand, residue K479 displays similar flexibility, evidenced by the increased temperature factor of the side chain upon reductive activation of the enzyme (Fig. S15B and Table S5†). Ordering of such a mobile residue upon substrate binding would be entropically unfavourable, but ultimately locks the lysine side chain in the observed conformation: the temperature factor is reduced to that of the rest of the canopy. The observed structure could thus represent a kinetic trap.

### 4.1 Consequences of replacing the active site arginine by lysine on catalytic kinetics

Once the inhibitory diatomic ligand has been removed, R479K displays catalytic activity that differs from Hyd-2 in two distinctive ways. First, the activity is much decreased, not only for H_2_ oxidation, as was originally observed when the equivalent arginine was replaced by lysine in Hyd-1,^27^ but also for H^+^ reduction. Although the R479K variant retains bidirectionality and behaves as a reversible electrocatalyst, there are large attenuations of catalytic activity as measured by steady-state solution kinetics, 2–10% for H_2_ oxidation (1 bar) and 0.4–2% for H^+^ reduction: values that may be compared alongside the 1% activity measured for H_2_ oxidation by Hyd-1 R509K. As with Hyd-1 R509K, the most striking observations lie in the temperature dependences of the rates. Unlike absolute turnover frequency, which in PFE always requires a knowledge of the electroactive coverage, the temperature dependence of rate, from which Δ*H*^‡^ is derived, is independent of coverage: Δ*H*^‡^ is therefore a diagnostic tool. A surprising feature of Hyd-1 R509K was that the low activity of that variant coincided with a significantly lowered activation enthalpy (by over 5 kJ mol^−1^), uniform over the temperature range 10–45°, which strongly suggested that the mechanism switches to one that is under entropic control (there being a greatly decreased probability of reaching the transition state). This behaviour is repeated for Hyd-2 R479K but with an additional interesting feature. Whereas the ≥98% drop in H^+^ reduction activity occurs with a uniform 1 kJ mol^−1^ decrease in Δ*H*^‡^ across the range 14–32 °C, which can again be interpreted in terms of a switch to entropic control, H_2_ oxidation shows a convex temperature dependence, Δ*H*^‡^ apparently varying from 23 kJ mol^−1^ at low temperature (a decrease of 24 kJ mol^−1^ compared to Hyd-2) to <0 kJ mol^−1^ above 23 °C. The low and variable value cannot be attributed to a favourable pre-equilibrium, which would have to involve a very stable intermediate, such as the Michaelis complex, the constant for which is actually higher than for Hyd-2.^12,13^ The absence of a significant kinetic isotope effect means that H-tunnelling, which could also account for a greatly lowered activation enthalpy, is not important in the rate-determining step. Despite the large decrease in rates, electrocatalysis remains reversible, as seen clearly in the voltammograms that show no evidence for additional overpotential requirement (any inflection) in the region where the current cuts the zero axis: this can be interpreted in terms of catalytic proton and electron transfers remaining tightly coupled rather than occurring in separate steps.^1,109^

Convex Eyring plots have long been considered to signify a change in rate-determining step as the temperature is varied; however, attention has turned recently to a richer interpretation. Work by Arcus, Mulholland and co-workers has highlighted and explained the role of heat capacity changes over the course of a reaction, with Δ*C*_p_‡ emerging as an important parameter in enzyme kinetics within the framework of macromolecular rate theory (MMRT).^110–113^ In MMRT, convex Eyring plots will tend to appear as the specific heat of the transition state becomes sufficiently small compared to that for the precursor enzyme–substrate complex. Low Δ*C*^‡^_p_ values are associated with a marked decrease in low-frequency vibrational modes, which can even originate in zones remote from the site of catalysis itself, and it is the large size of enzymes (and the consequent high number of such vibrational modes) that amplifies the contribution of Δ*C*^‡^_p_ compared to the reactions of small molecules.

### 4.2 Options available for the tightly bound ligand in the as-isolated or oxidised states

The as-isolated state of R479K has an extremely tightly bound ligand (X–Y) coordinated to the Ni atom (Fig. S27†) that blocks catalytic activity. The electron density maps are of high enough resolution (Table S3†) to allow confident assignment of ligand identity as a terminally-oriented diatomic species composed of light (2p) elements. The IR spectrum of the adduct is dominated by a blue-shifted *v*_CO_ at 2000 cm^−1^, evidence for back donation being greatly decreased and implying that the active site is in an oxidised state. The options for X–Y are CN^−^, CO, N_2_, NO and O_2_, which we now deal with in turn.

Cyanide is consistent with the requirement for the inactive complex to form under oxidising conditions (for first row transition metals, CN^−^ behaves mainly as a strong σ donor, and should stabilise Ni(iii) relative to Ni(ii)) and in terms of the bond angle (163°) that is slightly smaller than that observed for CO. Against this option: first, CN^−^ is a strong IR absorber and the new stretching band that would be expected to be observed was not detected (Fig. S16†); second, since the CN^−^ would have to originate from the breakdown of other active sites, a large fraction of the enzyme would be depleted, yet there is no evidence of this in the crystal structures and extensive reduction restored most of the activity. Moreover, redox titrations show that the formation of the high wavenumber species at 2000 cm^−1^ is reversible.

Carbon monoxide is consistent with the bond angle. However, inhibition by CO follows a completely different kinetic pattern (it being rapid and reversible), no new Ni–CO band was detected, and the inactive enzyme is formed under oxidising conditions that should not be favourable for a Ni–CO adduct. Furthermore, although a diatomic ligand was observed bound to the active site upon treatment with CO, no IR-band at 2000 cm^−1^ was observed.

Dinitrogen is an inert molecule under all but highly specialised catalytic conditions, and like CO, its coordination to a metal requires reducing conditions favouring an electron-rich site.

Nitric oxide coordination would be consistent with the bond angle, but it is particularly unclear where NO would originate from. In addition, NO is known to attack Fe–S clusters, and any such damage is not observed.

Dioxygen could bind in different forms, O_2_, O_2_^−^ (superoxide) or O_2_^2−^ (peroxide) depending on the oxidation state of the Ni, but under oxidising conditions superoxide bound to Ni(iii) would be more likely, and consistent with the lack of an EPR signal. However, the Ni–X–Y bond angle is much larger than expected for metal–O–O bonds; moreover, O_2_ is not required for appearance of the high wavenumber species in IR experiments.

A puzzling predicament thus arises as to the identity of ligand X–Y, as each of the above-listed options has serious drawbacks, yet the answer must lie in one of them. Taking into consideration the formation of the adduct under oxidising conditions, its inhibitory properties and the availability of a source of X–Y, the ligands N_2_, CO and NO look extremely unlikely, leaving CN^−^ (supported by bond angle) or an O_2_-derived ligand as the more likely options. The affinity for either ligand would have to be extremely high, to such a degree that the ligand is sequestered from an extremely dilute source. An alternative but even more unlikely scenario is that the ligand is an O–O species derived from two H_2_O: such an extreme case would require that the O–O species is stabilised to an unprecedented degree (in electrochemical terms, this would amount to an extreme case of underpotential deposition) so that it cannot be released without imposing a different condition (reduction back to H_2_O). Although such a high degree of stabilisation is indeed the case, we must regard this option as very unlikely.

## 5. Conclusions

Questions undoubtedly remain, but the conclusions arising from this work may be summarised as follows:

(1) For Hyd-2, as with its O_2_-tolerant counterpart Hyd-1 (ref. 27) the pendent arginine is essential for rapid H_2_ oxidation. This study now extends this observation to H_2_ production which is severely suppressed when the arginine is replaced by lysine. The mechanism of hydrogen activation remains unresolved but must include a key role for the pendent guanidinium group in each direction. In view of the fact that R479K incorporates other changes in structure in addition to the R-to-K exchange, it is not possible to attribute the large rate retardation to the replacement alone.

(2) The normal inner coordination sphere of the active site is essentially unchanged when lysine replaces arginine, emphasising the role of the outer sphere and wider active site.

(3) Despite greatly attenuated rates of H_2_ oxidation and proton reduction, the R479K variant remains a reversible electrocatalyst. Having a pendent guanidinium group is therefore not a factor for reversibility.

(4) The activation parameters can only be explained if the arginine to lysine substitution results in a great difficulty in sampling a productive conformation to achieve the transition state that is otherwise energetically more favourable than the native enzyme. The value of Δ*H*^‡^ for H_2_ oxidation is particularly low and the curvature of the Eyring plot may reflect a significant change in heat capacitance during the reaction.

(5) Replacement of arginine by lysine in the active site canopy results in an unexpected and unprecedented high affinity for a diatomic ligand that is coordinated to the Ni and hydrogen bonded to the lysine amine group. This ligand is bound very tightly in the as-isolated form and in samples subjected to oxidising conditions, whether aerobic or not.

(6) Extensive investigations to determine the identity of the strongly coordinated diatomic ligand remain inconclusive but in view of the fact that the adduct is formed under oxidising conditions the most likely options are a cyanide, possibly originating from active site breakdown, or a dioxygen species having unprecedented stability.

## Data availability

Coordinates and structure factors for each X-ray structure reported in this work have been deposited in the PDB with the following accession numbers: as-isolated enzyme (6SYO), hydrogen reduced enzyme (6SZD), oxygen exposed enzyme (6SYX) and CO exposed enzyme (6SZK).

## Author contributions

RME, SEB, SBC and FAA conceived and designed study. RME, SEB, SBC purified enzyme. RME, SEB, LK and FAA performed and analysed electrochemical and biochemical data. SBC performed X-ray structure determination. SEB and WKM performed EPR measurements. RME, KLW, PRM, PAA and KAV collected and analysed IR spectroscopic data. RB and YP performed DFT calculations. All authors contributed to writing the manuscript.

## Conflicts of interest

The authors report no conflict of interests.

## Supplementary Material

SC-014-D2SC05641K-s001

## References

[cit1] Armstrong F. A., Hirst J. (2011). Proc. Natl. Acad. Sci. U. S. A..

[cit2] Armstrong F. A., Evans R. M., Hexter S. V., Murphy B. J., Roessler M. M., Wulff P. (2016). Acc. Chem. Res..

[cit3] Boralugodage N. P., Arachchige R. J., Dutta A., Buchko G. W., Shaw W. J. (2017). Catal. Sci. Technol..

[cit4] Ginovska-Pangovska B., Dutta A., Reback M. L., Linehan J. C., Shaw W. J. (2014). Acc. Chem. Res..

[cit5] Schild J., Reuillard B., Morozan A., Chenevier P., Gravel E., Doris E., Artero V. (2021). J. Am. Chem. Soc..

[cit6] Smith S. E., Yang J. Y., DuBois D. L., Bullock R. M. (2012). Angew. Chem., Int. Ed..

[cit7] Greening C., Biswas A., Carere C. R., Jackson C. J., Taylor M. C., Stott M. B., Cook G. M., Morales S. E. (2015). ISME J..

[cit8] Murphy B. J., Sargent F., Armstrong F. A. (2014). Energy Environ. Sci..

[cit9] Cracknell J. A., Wait A. F., Lenz O., Friedrich B., Armstrong F. A. (2009). Proc. Natl. Acad. Sci. U. S. A..

[cit10] Lukey M. J., Roessler M. M., Parkin A., Evans R. M., Davies R. A., Lenz O., Friedrich B., Sargent F., Armstrong F. A. (2011). J. Am. Chem. Soc..

[cit11] Evans R. M., Parkin A., Roessler M. M., Murphy B. J., Adamson H., Lukey M. J., Sargent F., Volbeda A., Fontecilla-Camps J. C., Armstrong F. A. (2013). J. Am. Chem. Soc..

[cit12] Beaton S. E., Evans R. M., Finney A. J., Lamont C. M., Armstrong F. A., Sargent F., Carr S. B. (2018). Biochem. J..

[cit13] Lukey M. J., Parkin A., Roessler M. M., Murphy B. J., Harmer J., Palmer T., Sargent F., Armstrong F. A. (2010). J. Biol. Chem..

[cit14] Dole F., Fournel A., Magro V., Hatchikian E. C., Bertrand P., Guigliarelli B. (1997). Biochemistry.

[cit15] Huyett J. E., Carepo M., Pamplona A., Franco R., Moura I., Moura J. J. G., Hoffman B. M. (1997). J. Am. Chem. Soc..

[cit16] Lubitz W., Ogata H., Rüdiger O., Reijerse E. (2014). Chem. Rev..

[cit17] Rakowski DuBois M., DuBois D. L. (2009). Chem. Soc. Rev..

[cit18] Ginovska-Pangovska B., Dutta A., Reback M. L., Linehan J. C., Shaw W. J. (2014). Acc. Chem. Res..

[cit19] Slater J. W., Marguet S. C., Monaco H. A., Shafaat H. S. (2018). J. Am. Chem. Soc..

[cit20] Slater J. W., Marguet S. C., Gray M. E., Sotomayor M., Shafaat H. S. (2019). ACS Catal..

[cit21] Ogo S., Ichikawa K., Kishima T., Matsumoto T., Nakai H., Kusaka K., Ohhara T. (2013). Science.

[cit22] Wombwell C., Reisner E. (2015). Chem. - Eur. J..

[cit23] Armstrong F. A., Cheng B., Herold R. A., Megarity C. F., Siritanaratkul B. (2023). Chem. Rev..

[cit24] Dementin S., Burlat B., De Lacey A. L., Pardo A., Adryanczyk-Perrier G., Guigliarelli B., Fernandez V. M., Rousset M. (2004). J. Biol. Chem..

[cit25] Evans R. M. and Beaton S. E., Enzymes of Energy Technology, in Methods in Enzymology, ed. F. A. Armstrong, 2018, pp. 91–116

[cit26] Greene B. L., Vansuch G. E., Wu C.-H., Adams M. W. W., Dyer R. B. (2016). J. Am. Chem. Soc..

[cit27] Evans R. M., Brooke E. J., Wehlin S. A. M., Nomerotskaia E., Sargent F., Carr S. B., Phillips S. E. V., Armstrong F. A. (2016). Nat. Chem. Biol..

[cit28] Brooke E. J., Evans R. M., Islam S. T. A., Roberts G. M., Wehlin S. A. M., Carr S. B., V Phillips S. E., Armstrong F. A. (2017). Biochemistry.

[cit29] Foerster S., Van Gastel M., Brecht M., Lubitz W. (2005). J. Biol. Inorg. Chem..

[cit30] Brecht M., van Gastel M., Buhrke T., Friedrich B., Lubitz W. (2003). J. Am. Chem. Soc..

[cit31] Ogata H., Krämer T., Wang H., Schilter D., Pelmenschikov V., van Gastel M., Neese F., Rauchfuss T. B., Gee L. B., Scott A. D., Yoda Y., Tanaka Y., Lubitz W., Cramer S. P. (2015). Nat. Commun..

[cit32] Ogata H., Nishikawa K., Lubitz W. (2015). Nature.

[cit33] Dementin S., Burlat B., De Lacey A. L., Pardo A., Adryanczyk-Perrier G., Guigliarelli B., Fernandez V. M., Rousset M. (2004). J. Biol. Chem..

[cit34] Evans R. M., Ash P. A., Beaton S. E., Brooke E. J., Vincent K. A., Carr S. B., Armstrong F. A. (2018). J. Am. Chem. Soc..

[cit35] Szőri-Dorogházi E., Maróti G., Szőri M., Nyilasi A., Rákhely G., Kovács K. L. (2012). PLoS One.

[cit36] Vansuch G. E., Wu C.-H., Haja D. K., Blair S. A., Chica B., Johnson M. K., Adams M. W. W., Dyer R. B. (2020). Chem. Sci..

[cit37] Escorcia A. M., Stein M. (2018). Front. Chem..

[cit38] Stephan D. W., Erker G. (2010). Angew. Chem., Int. Ed..

[cit39] Birrell J. A., Rodríguez-Maciá P., Reijerse E. J., Martini M. A., Lubitz W. (2021). Coord. Chem. Rev..

[cit40] Berggren G., Adamska A., Lambertz C., Simmons T. R., Esselborn J., Atta M., Gambarelli S., Mouesca J. M., Reijerse E., Lubitz W., Happe T., Artero V., Fontecave M. (2013). Nature.

[cit41] Esselborn J., Muraki N., Klein K., Engelbrecht V., Metzler-Nolte N., Apfel U.-P., Hofmann E., Kurisu G., Happe T. (2016). Chem. Sci..

[cit42] Voet D. and Voet J. G., Biochemistry, John Wiley & Sons, Inc., New York., 4th edn, 2011

[cit43] Pankhurst K. L., Mowat C. G., Rothery E. L., Hudson J. M., Jones A. K., Miles C. S., Walkinshaw M. D., Armstrong F. A., Reid G. A., Chapman S. K. (2006). J. Biol. Chem..

[cit44] Doherty M. K., Pealing S. L., Miles C. S., Moysey R., Taylor P., Walkinshaw M. D., Reid G. A., Chapman S. K. (2000). Biochemistry.

[cit45] Guillén Schlippe Y. V., Hedstrom L. (2005). Arch. Biochem. Biophys..

[cit46] Tedeschi G., Ronchi S., Simonic T., Treu C., Mattevi A., Negri A. (2001). Biochemistry.

[cit47] Hong J. H., Dilbeck P., Debus R. J., Burnap R. L. (2007). Biochemistry.

[cit48] Van Der Kamp M. W., Perruccio F., Mulholland A. J. (2008). Chem. Commun..

[cit49] Kwon H., Basran J., Devos J. M., Suardíaz R., van der Kamp M. W., Mulholland A. J., Schrader T. E., Ostermann A., Blakeley M. P., Moody P. C. E., Raven E. L. (2020). Proc. Natl. Acad. Sci. U. S. A..

[cit50] Fitch C. A., Platzer G., Okon M., Garcia-Moreno B. E., McIntosh L. P. (2015). Protein Sci..

[cit51] Lučić M., Svistunenko D. A., Wilson M. T., Chaplin A. K., Davy B., Ebrahim A., Axford D., Tosha T., Sugimoto H., Owada S., Dworkowski F. S. N., Tews I., Owen R. L., Hough M. A., Worrall J. A. R. (2020). Angew. Chem., Int. Ed..

[cit52] Lučić M., Wilson M. T., Svistunenko D. A., Owen R. L., Hough M. A., Worrall J. A. R. (2021). J. Biol. Inorg. Chem..

[cit53] Morris R. H. (2014). J. Am. Chem. Soc..

[cit54] Kwon H., Basran J., Devos J. M., Suardíaz R., van der Kamp M. W., Mulholland A. J., Schrader T. E., Ostermann A., Blakeley M. P., Moody P. C. E., Raven E. L. (2020). Proc. Natl. Acad. Sci. U. S. A..

[cit55] Fitch C. A., Platzer G., Okon M., Garcia-Moreno B. E., McIntosh L. P. (2015). Protein Sci..

[cit56] Lučić M., Svistunenko D. A., Wilson M. T., Chaplin A. K., Davy B., Ebrahim A., Axford D., Tosha T., Sugimoto H., Owada S., Dworkowski F. S. N., Tews I., Owen R. L., Hough M. A., Worrall J. A. R. (2020). Angew. Chem., Int. Ed..

[cit57] Lučić M., Wilson M. T., Svistunenko D. A., Owen R. L., Hough M. A., Worrall J. A. R. (2021). J. Biol. Inorg. Chem..

[cit58] Hamilton C. M., Aldea M., Washburn B. K., Babitzke P., Kushner S. R. (1989). J. Bacteriol..

[cit59] Lamont C. M., Sargent F. (2016). Arch. Microbiol..

[cit60] Bard A. J. and Faulkner L. R., Electrochemical methods, Wiley, New york, 2001

[cit61] Rüdiger O., Abad J. M., Hatchikian E. C., Fernandez V. M., De Lacey A. L. (2005). J. Am. Chem. Soc..

[cit62] Krishnan S., Armstrong F. A. (2012). Chem. Sci..

[cit63] Cammack R. , Fernandez V. M., Claude Hatchikian E. and Peck J. J. L. H. D., in Methods in Enzymology, Academic Press, 1994, vol. 243, pp. 43–68

[cit64] Bradford M. M. (1976). Anal. Biochem..

[cit65] Waterman D. G. , Winter G., Parkhurst J. M., Fuentes-Montero L., Hattne J., Brewster A., Sauter N. K. and Evans G., CCP4 Newsletter on Protein Crystallography, 2013, vol. 49, pp. 16–19

[cit66] Evans P. R., Murshudov G. N. (2013). Acta Crystallogr., Sect. D: Biol. Crystallogr..

[cit67] McCoy A. J., Grosse-Kunstleve R. W., Adams P. D., Winn M. D., Storoni L. C., Read R. J. (2007). J. Appl. Crystallogr..

[cit68] Emsley P., Lohkamp B., Scott W. G., Cowtan K. (2010). Acta Crystallogr., Sect. D: Biol. Crystallogr..

[cit69] Murshudov G. N., Skubak P., Lebedev A. A., Pannu N. S., Steiner R. A., Nicholls R. A., Winn M. D., Long F., Vagin A. A. (2011). Acta Crystallogr., Sect. D: Biol. Crystallogr..

[cit70] Tian W., Chen C., Lei X., Zhao J., Liang J. (2018). Nucleic Acids Res..

[cit71] Jurcik A., Bednar D., Byska J., Marques S. M., Furmanova K., Daniel L., Kokkonen P., Brezovsky J., Strnad O., Stourac J., Pavelka A., Manak M., Damborsky J., Kozlikova B. (2018). Bioinformatics.

[cit72] L DeLano W. , {CCP4} Newsletter On Protein Crystallography, 2002, 40

[cit73] Stoll S., Schweiger A. (2006). J. Magn. Reson..

[cit74] Ash P. A., Kendall-Price S. E. T., Evans R. M., Carr S. B., Brasnett A., Morra S., Hidalgo R., Healy A. J., Cinque G., Frogley M. D., Armstrong F. A., Vincent K. A. (2021). Chem. Sci..

[cit75] Neese F. (2022). Wiley Interdiscip. Rev. Comput. Mol. Sci..

[cit76] Eastman P., Swails J., Chodera J. D., McGibbon R. T., Zhao Y., Beauchamp K. A., Wang L. P., Simmonett A. C., Harrigan M. P., Stern C. D., Wiewiora R. P., Brooks B. R., Pande V. S. (2017). PLoS Comput. Biol..

[cit77] Best R. B., Zhu X., Shim J., Lopes P. E. M., Mittal J., Feig M., MacKerell A. D. (2012). J. Chem. Theory Comput..

[cit78] Jorgensen W. L., Chandrasekhar J., Madura J. D., Impey R. W., Klein M. L. (1983). J. Chem. Phys..

[cit79] Sun J., Ruzsinszky A., Perdew J. (2015). Phys. Rev. Lett..

[cit80] Furness J. W., Kaplan A. D., Ning J., Perdew J. P., Sun J. (2020). J. Phys. Chem. Lett..

[cit81] Ehlert S., Huniar U., Ning J., Furness J. W., Sun J., Kaplan A. D., Perdew J. P., Brandenburg J. G. (2021). J. Chem. Phys..

[cit82] Caldeweyher E., Bannwarth C., Grimme S. (2017). J. Chem. Phys..

[cit83] Van Wüllen C. (1998). J. Chem. Phys..

[cit84] Van Lenthe E., Van Leeuwen R., Baerends E. J., Snijders J. G. (1996). Int. J. Quantum Chem..

[cit85] Weigend F., Ahlrichs R. (2005). Phys. Chem. Chem. Phys..

[cit86] Pantazis D. A., Chen X. Y., Landis C. R., Neese F. (2008). J. Chem. Theory Comput..

[cit87] Neese F. (2003). J. Comput. Chem..

[cit88] Weigend F. (2006). Phys. Chem. Chem. Phys..

[cit89] Fourmond V., Wiedner E. S., Shaw W. J., Léger C. (2019). J. Am. Chem. Soc..

[cit90] Evans R. M., Siritanaratkul B., Megarity C. F., Pandey K., Esterle T. F., Badiani S., Armstrong F. A. (2019). Chem. Soc. Rev..

[cit91] Vincent K. A., Parkin A., Armstrong F. A. (2007). Chem. Rev..

[cit92] Pershad H. R., Duff J. L. C., Heering H. A., Duin E. C., Albracht S. P. J., Armstrong F. A. (1999). Biochemistry.

[cit93] Evans R. M., Siritanaratkul B., Megarity C. F., Pandey K., Esterle T. F., Badiani S., Armstrong F. A. (2019). Chem. Soc. Rev..

[cit94] Evans R. M. and Armstrong F. A., in Metalloproteins - Methods and Protocols, ed. J. C. Fontecilla-Camps and Y. Nicolet, Humana Press, New York, 2014, pp. 73–94

[cit95] Armstrong F. A., Belsey N. A., Cracknell J. A., Goldet G., Parkin A., Reisner E., Vincent K. A., Wait A. F. (2009). Chem. Soc. Rev..

[cit96] Leger C., Dementin S., Bertrand P., Rousset M., Guigliarelli B., Léger C., Dementin S., Bertrand P., Rousset M., Guigliarelli B. (2004). J. Am. Chem. Soc..

[cit97] Goris T., Wait A. F., Saggu M., Fritsch J., Heidary N., Stein M., Zebger I., Lendzian F., Armstrong F. A., Friedrich B., Lenz O. (2011). Nat. Chem. Biol..

[cit98] Hexter S. V., Grey F., Happe T., Climent V., Armstrong F. A. (2012). Proc. Natl. Acad. Sci. U. S. A..

[cit99] Dragelj J., Karafoulidi-Retsou C., Katz S., Lenz O., Zebger I., Caserta G., Sacquin-Mora S., Mroginski M. A. (2023). Front. Microbiol..

[cit100] Volbeda A., Martin L., Cavazza C., Matho M., Faber B. W., Roseboom W., Albracht S. P. J., Garcin E., Rousset M., Fontecilla-Camps J. C. (2005). J. Biol. Inorg. Chem..

[cit101] Volbeda A., Martin L., Barbier E., Gutiérrez-Sanz O., De Lacey A. L., Liebgott P.-P., Dementin S., Rousset M., Fontecilla-Camps J. C. (2015). JBIC, J. Biol. Inorg. Chem..

[cit102] Ilina Y., Lorent C., Katz S., Jeoung J.-H., Shima S., Horch M., Zebger I., Dobbek H. (2019). Angew. Chem..

[cit103] Tai H., Nishikawa K., Higuchi Y., Mao Z.-W., Hirota S. (2019). Angew. Chem..

[cit104] Pandelia M. E., Ogata H., Currell L. J., Flores M., Lubitz W. (2010). Biochim. Biophys. Acta, Bioenerg..

[cit105] Ogata H., Mizoguchi Y., Mizuno N., Miki K., Adachi S., Yasuoka N., Yagi T., Yamauchi O., Hirota S., Higuchi Y. (2002). J. Am. Chem. Soc..

[cit106] Shomura Y., Taketa M., Nakashima H., Tai H., Nakagawa H., Ikeda Y., Ishii M., Igarashi Y., Nishihara H., Yoon K. S., Ogo S., Hirota S., Higuchi Y. (2017). Science.

[cit107] Kulka-Peschke C. J., Schulz A. C., Lorent C., Rippers Y., Wahlefeld S., Preissler J., Schulz C., Wiemann C., Bernitzky C. C. M., Karafoulidi-Retsou C., Wrathall S. L. D., Procacci B., Matsuura H., Greetham G. M., Teutloff C., Lauterbach L., Higuchi Y., Ishii M., Hunt N. T., Lenz O., Zebger I., Horch M. (2022). J. Am. Chem. Soc..

[cit108] Kumar R., Stein M. (2023). J. Am. Chem. Soc..

[cit109] Lampret O., Duan J., Hofmann E., Winkler M., Armstrong F. A., Happe T. (2020). Proc. Natl. Acad. Sci. U. S. A..

[cit110] Arcus V. L., van der Kamp M. W., Pudney C. R., Mulholland A. J. (2020). Curr. Opin. Struct. Biol..

[cit111] Bunzel H. A., Anderson J. L. R., Hilvert D., Arcus V. L., van der Kamp M. W., Mulholland A. J. (2021). Nat. Chem..

[cit112] Arcus V. L., Mulholland A. J. (2020). Annu. Rev. Biophys..

[cit113] Bunzel H. A., Kries H., Marchetti L., Zeymer C., Mittl P. R. E., Mulholland A. J., Hilvert D. (2019). J. Am. Chem. Soc..

