# Supplementary material for: Comprehensive structural, infrared spectroscopic and kinetic investigations of the roles of the active-site arginine in bidirectional hydrogen activation by the [NiFe]-hydrogenase ‘Hyd-2’ from Escherichia coli
Source: Chem Sci. 2023 Jul 25;14(32):8531–51. doi: 10.1039/d2sc05641k (PMC10430524; doi:10.1039/d2sc05641k)
Supplement: SC-014-D2SC05641K-s001 [file SC-014-D2SC05641K-s001.pdf]

Electronic Supplementary Material (ESI) for Chemical Science.
This journal is © The Royal Society of Chemistry 2023

Comprehensive Structural, Infrared Spectroscopic and Kinetic Investigations of the Roles of the active-site Arginine in Bidirectional Hydrogen Activation by the [NiFe]-hydrogenase 'Hyd-2' from *Escherichia coli*

Rhiannon M. Evans,[1] Stephen E. Beaton,[1] Patricia Rodriguez Macia,[1] Yunjie Pang,[3,4] Kin Long Wong, [1,2] Leonie Kertess,[1] William K. Myers,[1] Ragnar Bjornsson,[4,5] Philip A Ash,[6] Kylie A. Vincent,[1] Stephen B. Carr[1,2*] and Fraser, A. Armstrong[1*]

[1]University of Oxford, Department of Chemistry, South Parks Road, Oxford, U.K., [2]Research Complex at Harwell, Rutherford Appleton Laboratory, Harwell Campus, Didcot, U.K. [3]College of Chemistry, Beijing Normal University, 100875, Beijing, China. [4]Department of Inorganic Spectroscopy, Max Planck Institute for Chemical Energy Conversion, Stiftstraße 34-36, 45470 Mülheim an der Ruhr, Germany. [5]Laboratoire Chimie et Biologie des Métaux, CEA, 17 Av. Des Martyrs, 38000 Grenoble, France. [6]School of Chemistry, The University of Leicester, University Road, Leicester, LE1 7RH, U.K.

*corresponding authors

stephen.carr@rc-harwell.ac.uk; fraser.armstrong@chem.ox.ac.uk

Supplementary Figures and Tables

Table of Contents

Strains, primers and plasmids....3
SDS-PAGE of R479K 4
The electrocatalytic fingerprints of Hyd-2 and R479K....5
The potential dependence of $K_M(H_2)$ for R479K at 20 °C....7
Product inhibition and determination of K_i H_2 8
Stability of oxidised inactive state(s) 9
Response of R479K to transient exposure to O_2 10
The temperature dependence of H_2 oxidation and H^+ reduction current for Hyd-2 and R479K....11
Kinetic Isotope Effect....12
Determination of the activation entropy difference ($\Delta\Delta S^‡$) between Hyd-2 and R479K 13
$\Delta\Delta S^‡$ of H_2 oxidation 13
$\Delta\Delta S^‡$ of H^+ reduction....13
X-ray collection and refinement statistics 15
R509K vs R479K active site H-bonding 16
Electron density distribution across diatomic ligand....17
Modelling the diatomic ligand in the active site 19
R509K active site ordered water molecules 20
R479K active site bonding involving gaseous entities 21
Temperature factors of atoms in the Active site....22
Infrared spectra in the vCN region 23
IR-spectra of poised R479K crystals before and after CO exposure 24
IR-spectra of electrochemical redox titrations of R479K single crystals....26
Theoretical Methods used for Analysis of Hyd-2 R479K Structures 28
QM/MM results for different ligands bound to R479K 30
Results of the QM-cluster and QM/MM models with an O_2-bound ligand....32
Diatomic ligand bound to pocket next to active site Ni atom 35
Supplementary References 36

Strains, primers and plasmids

Table S1: Strains, primers, and plasmids used in the construction of Hyd-2 R479K.

Strain	Genotype	Purpose	Source
HJ001-hyp	As HJ001, ΔtatD::hypA1-X	Expression strain for Hyd-2	Reference [1]
HJ001-hyp R479K	As HJ001-hyp, *hybC* R479K	Expression strain for R479K	This Work

Primer Name	Primer Function	Primer Sequence	Source
hybC_R479K_F	Amplify R479K forwards primer with pMAK overlap	5'-GTGTTGGCTTCCTCGAAGCGCCGAAAGG TATGCTCTCTCACTGGATGGTTATTAAAG-3'	This Work
hybC_R479K_R	Amplify R479K reverse primer with pMAK overlap	5'-CTTTAATAACCATCCAGTGAGAGAGCAT ACCTTTCGGCGCTTCGAGGAAGCCAACAC-3'	This Work
pMAK_overlap_F	Amplify linear pMAK construct forwards primer	5'-CGGTCCAATGATCGAAGTTAGGCTGG TAAGAGCCG-3'	Reference [2]
pMAK_overlap_R	Amplify linear pMAK construct reverse primer	5'-CGGCTCTTACCAGCCTAACTTCGATCATT GGACCG-3'	Reference [2]

Plasmid	Identifier	Purpose	Source
pQE-80L hybO ΔTAT ΔTM C-terminal hexa-his tag	pO^{C}	Used for overproduction of soluble hybO with C-terminal hexa-his tag	This work
pMAK native *hybC*	pHybC	Used as parent plasmid for making the point mutations	Unpublished Work
pMAK *hybC* R479K	pR479K	Used to make HJ001-hyp R479K.	This work

SDS-PAGE of R479K

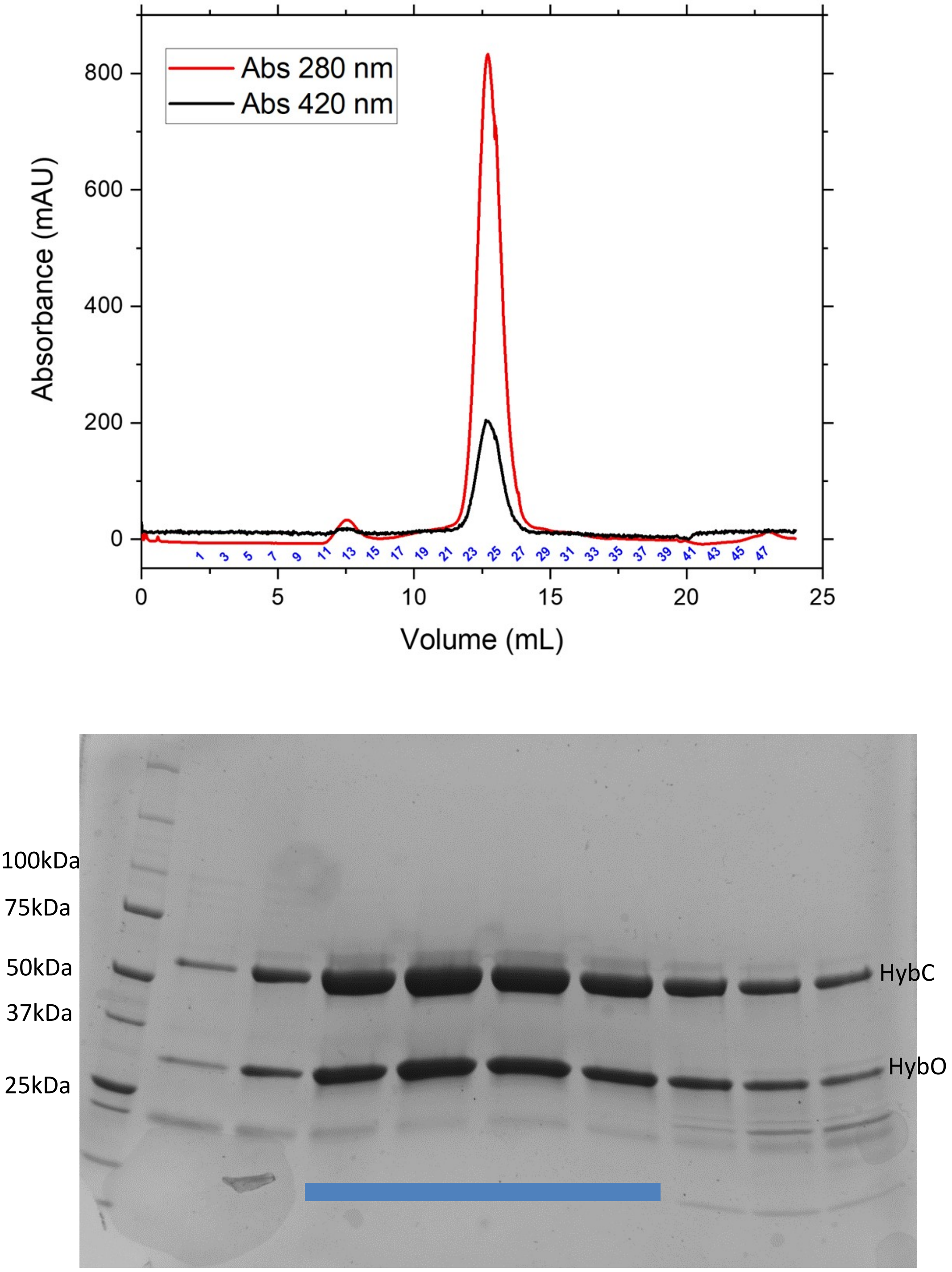

Figure S1: (Top) Size exclusion chromatogram of R479K purification using a Superdex 200 column (GEHealthcare) immediately following Ni-affinity chromatography. (Bottom) SDS-PAGE denaturing gel of fractions of R479K during purification by size exclusion chromatography. A sample (10 µL) was mixed with an equal amount of SDS-PAGE loading dye, boiled, and loaded. SDS-PAGE gel run at 300 V for approx. 20 min. HybC (61 kDa) and HybO (33 kDa) are labelled. Molecular weight measured by comparison with Kalaedoscope™ precision plus pre-stained Molecular-Weight Markers (Biorad, UK). Fractions pooled for further analysis are indicated by the blue bar.

The electrocatalytic fingerprints of Hyd-2 and R479K

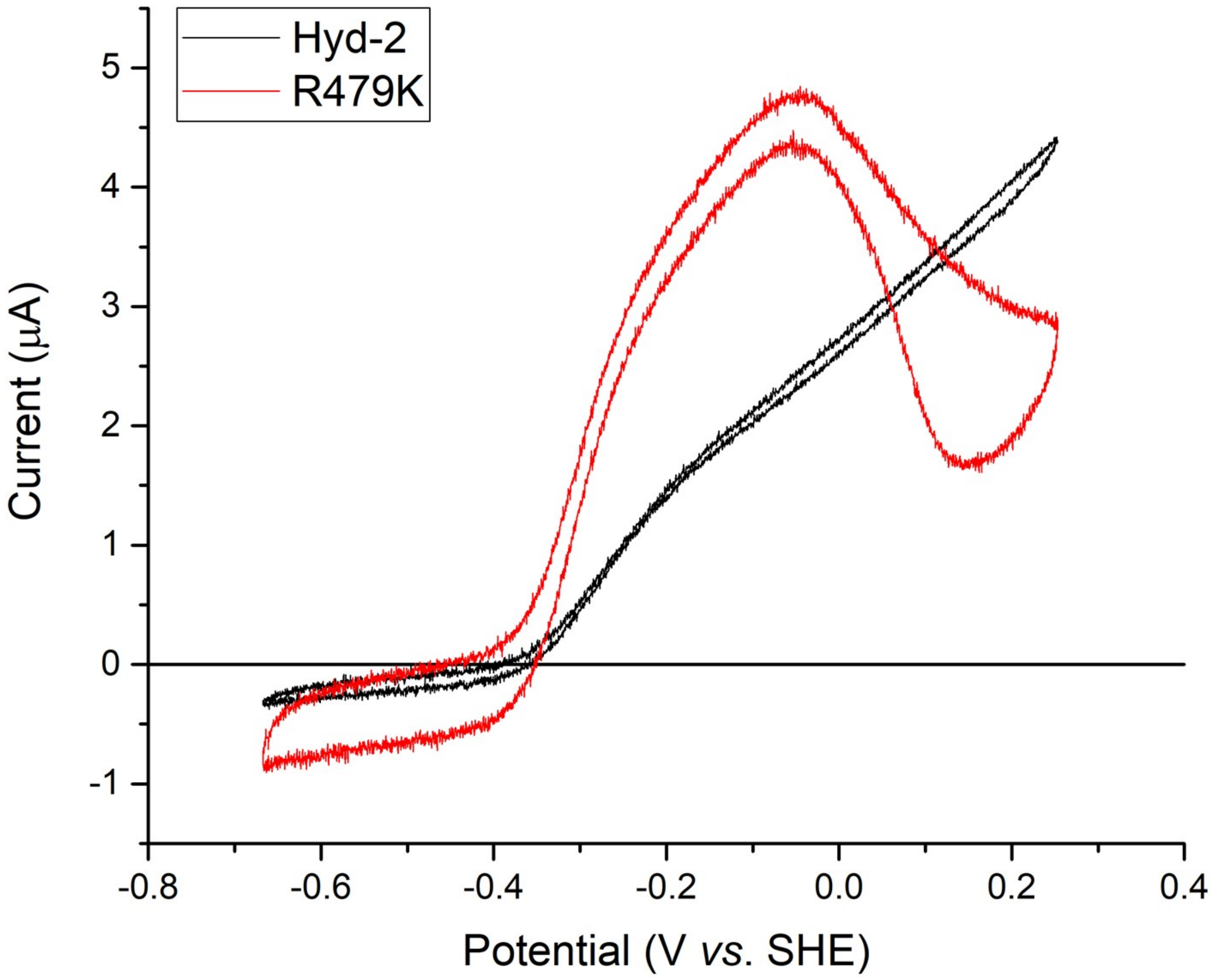

Figure S2: Cyclic voltammograms under 100% H_2 for Hyd-2 enzymes. Hyd-2 is shown in black and R479K is in red. While both current maximums for the two scans have been deliberately made comparable (see methods), the shape of the two voltammograms vary widely. Conditions: pH 6.0, 30 ^{o}C, 5 mV/s scan rate, 100% H_2 at 1000 scc min^{-1}, ω= 3000 rpm.

Demonstrating K_M H_2 and K_i H_2

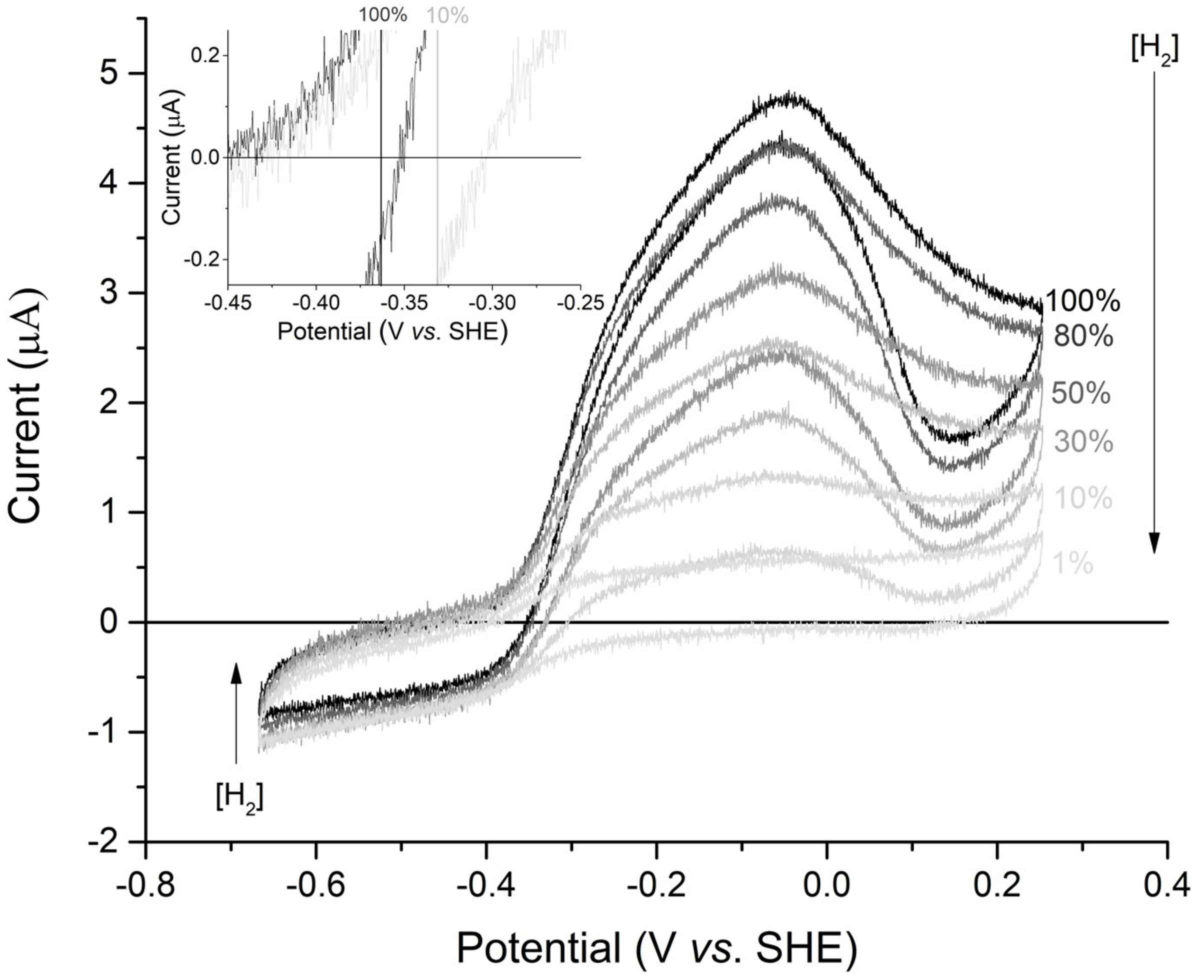

Figure S3: Varying the concentration of H_2 for the determination of $K_M(H_2)$ is exemplified here by the R479K variant. Increasing H_2 oxidation activity corresponds with increasing H_2 concentration and the zero-current potential corresponds well to the values predicted by the Nernst equation (inset). For proton reduction the activity increases with decreasing H_2 concentration, indicative of product inhibition, but to a lesser degree than that seen with native enzyme (see text). Other conditions: scan rate 5 mV/s, 30 oC, pH 6, Ar carrier gas, total gas flow rate 1000 scc min-1, ω = 3000 rpm.

The potential dependence of $K_M(H_2)$ for R479K at 20 °C

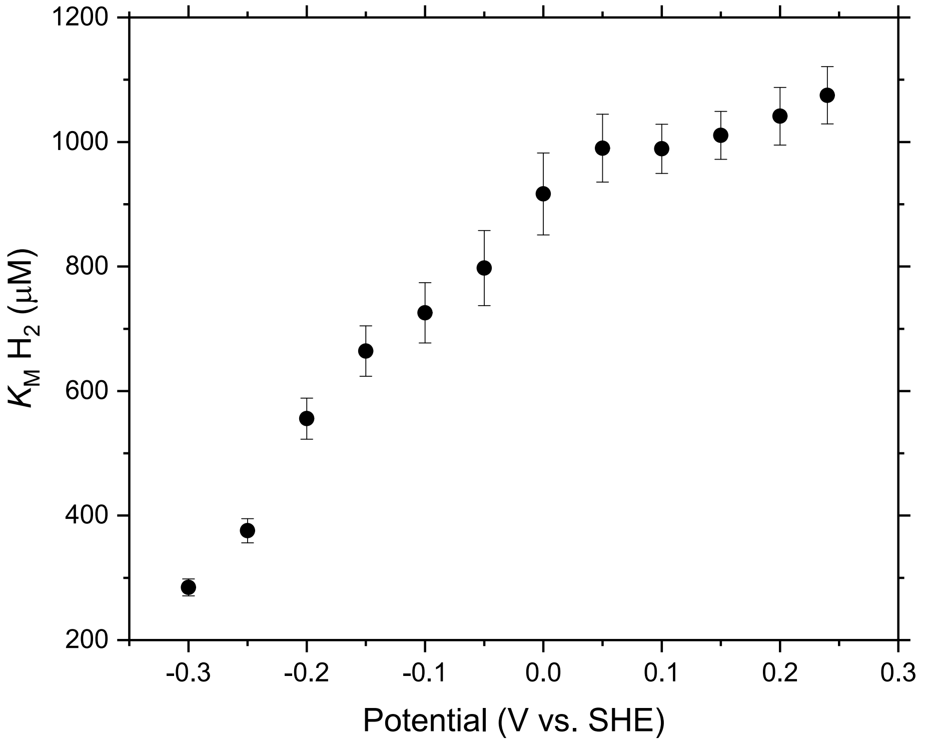

Figure S4: The potential dependence of $K_M(H_2)$ for R479K at 20 °C, pH 6.0. Cyclic voltammograms at a scan rate of 5 mV/s were recorded at various concentrations of H_2. The value for K_M was determined by plotting the average current at each potential for each H_2 concentration and plotting as Michaelis Menten or Hanes-Wolf plots in the Origin graphical software package.

Product inhibition and determination of K_i H_2

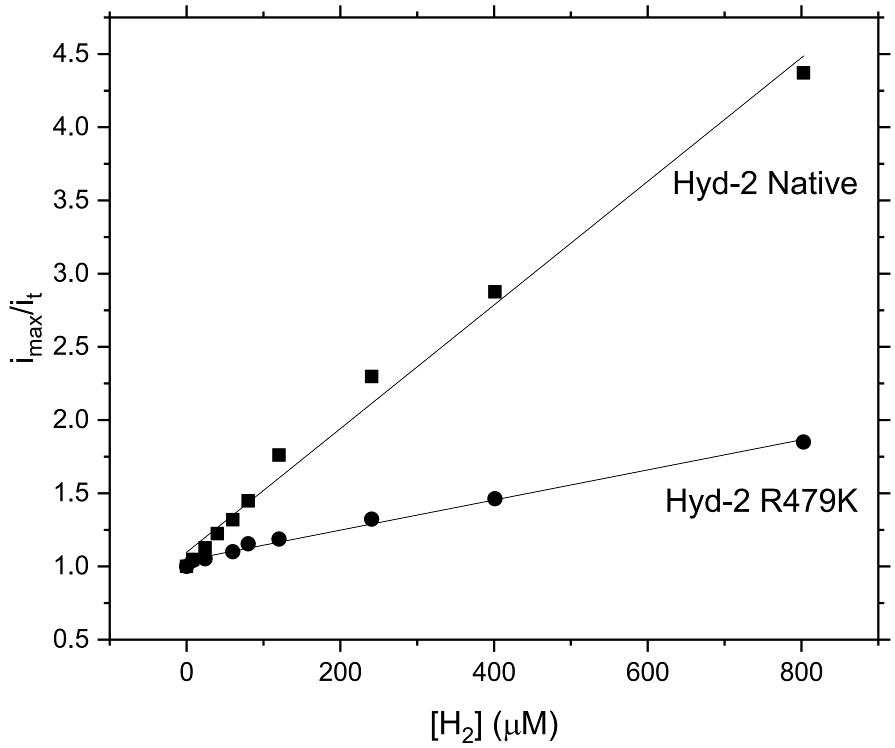

Figure S5: For the determination of the inhibition constant for H_2 during H^+ reduction, the current response to [H_2] was monitored for Hyd-2 and R479K at -0.66 V. The maximum current (i_{max}) at 100% Ar (zero H_2) is divided by the current at all other H_2 concentrations (i_t) and plotted against H_2 concentration. Fitting to a linear equation gives an intercept of 1/Ki. Other conditions: pH 6.0, 30 °C, Ar carrier gas, total gas flow rate 1000 scc min^{-1}, ω = 3000 rpm.

Stability of oxidised inactive state(s)

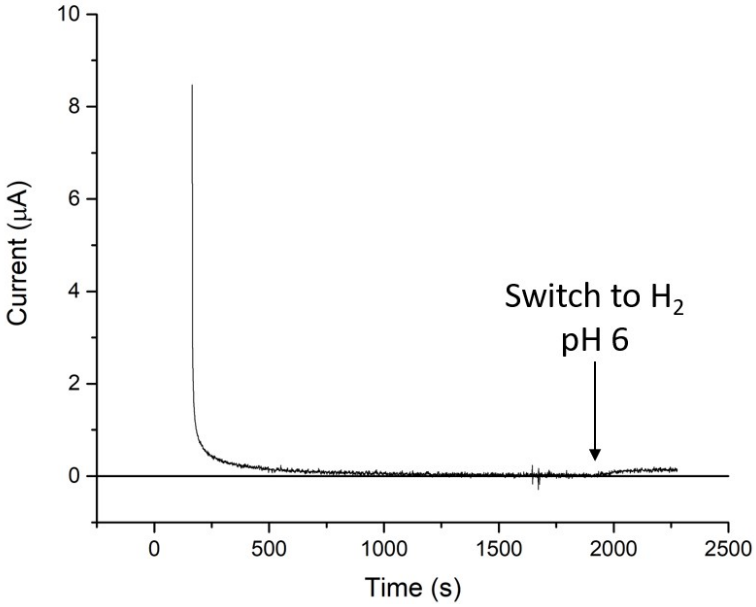

Figure S6: Complete anaerobic inactivation of Hyd-2 R479K for determining the stability of oxidised inactive states. The enzyme is fully inactivated in 100% Ar at pH 8 by holding the electrode at approx. +0.36 V. The gas is then switched to 100% H_2 and simultaneously the buffer is exchanged to pH 6 by at least 10-fold volume exchange. After approx. 300 sec to allow for full gas and temperature equilibration of the pH 6 buffer, the cyclic voltammogram from high to low potential is started at 0.1 mV/s (see Figure 7). Other conditions: 30 °C, total gas flow rate 1000 scc min^{-1}, ω = 3000 rpm.

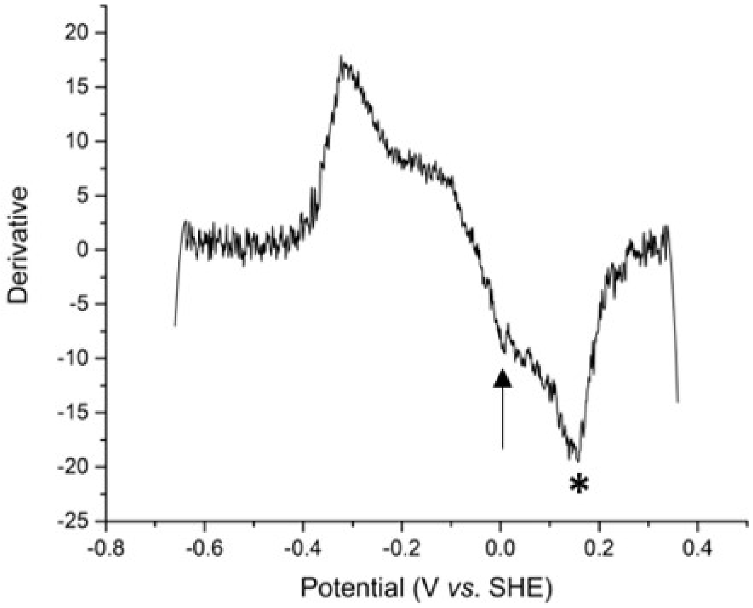

Figure S7: The first derivative of the backwards scan (Figure 7) for R479K. The asterisked minimum represents the strong inflection point used to denote E_{switch}[3,4] and the arrow indicates a second minimum close to the typical potential of E_{switch} for Hyd-2. Data have been smoothed with the Savitzky-Golay method, 41 points per window, using Origin software.

Response of R479K to transient exposure to O_2

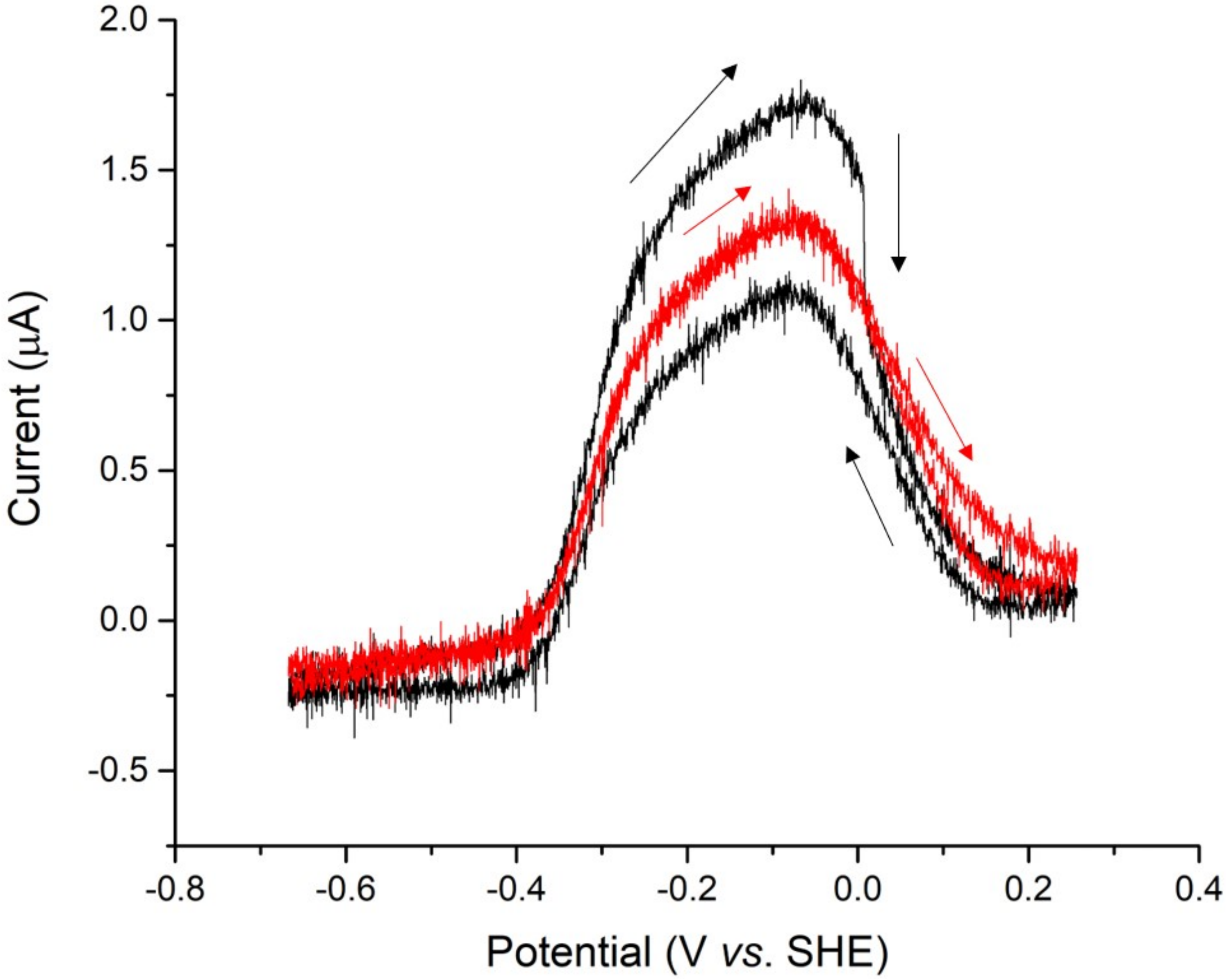

Figure S8: Consecutive cyclic voltammograms of R479K after O_2 exposure. The R479K variant was exposed to O_2 to an initial concentration of 130 µM within a constant H_2 flow. The first scan during which O_2 was injected at 0 V is in black. After the scan was complete a second scan was performed (red). Conditions: 30 °C, pH 6, scan rate 0.5 mV/s, ω=3000 rpm. Scans have not been corrected for film loss.

The temperature dependence of H_2 oxidation and H^+ reduction current for Hyd-2 and R479K

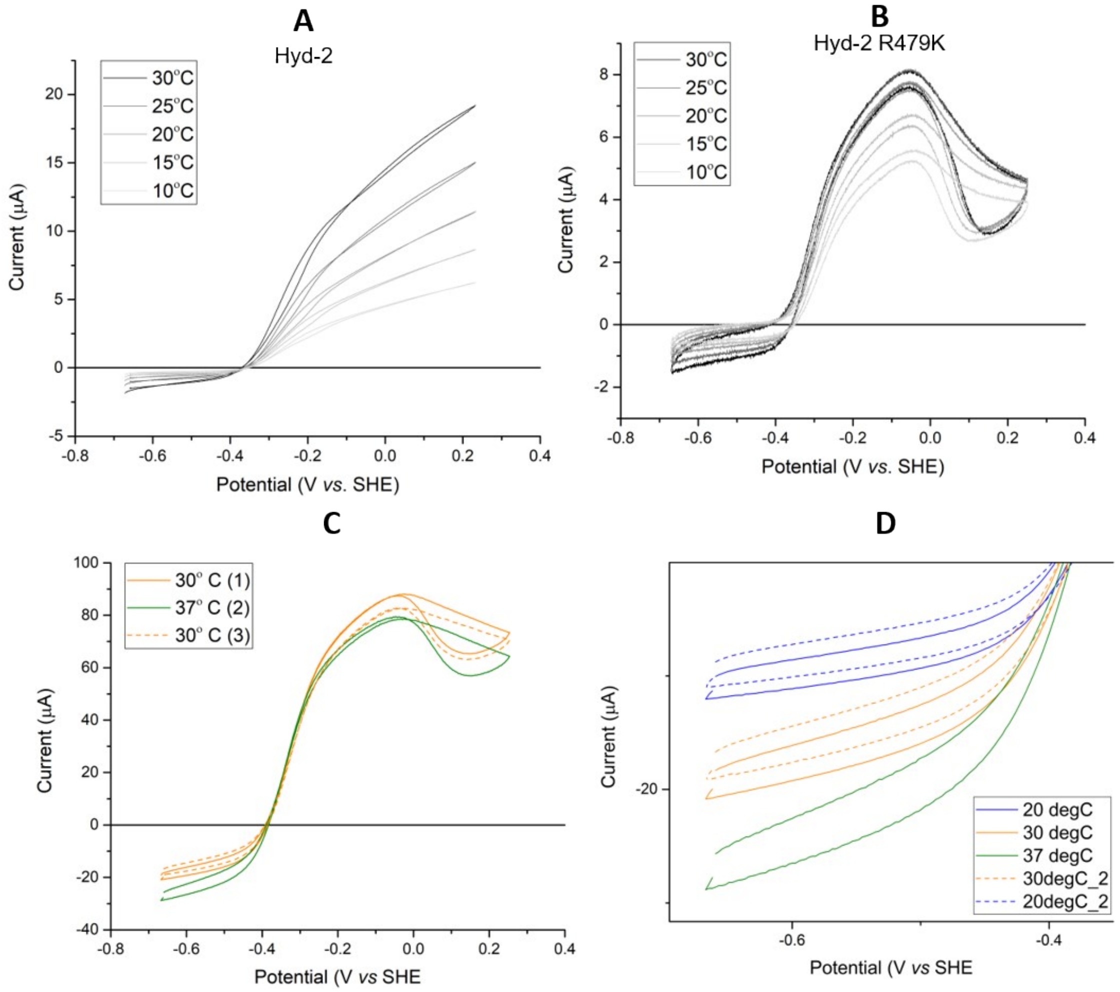

Figure S9: Cyclic voltammograms at varying temperatures for Hyd-2, (A) and R479K (B-D). The relative rate of H_2 oxidation does not track with increasing temperature above 30 °C (C) whereas the relative rate of H^+ reduction increases with temperature across the temperature range (D). For panels C and D, to increase film stability the film of R479K was prepared using a standard PGE electrode coated with multi-walled carbon nanotubes and pyrene-butyric acid for covalent attachment of the enzyme via its surface lysine residues as described previously.[5–7] Conditions: pH 6, scan rate 5 mV/s, 100% H_2, ω = 3000 rpm. These scans are not corrected for film loss.

Kinetic Isotope Effect

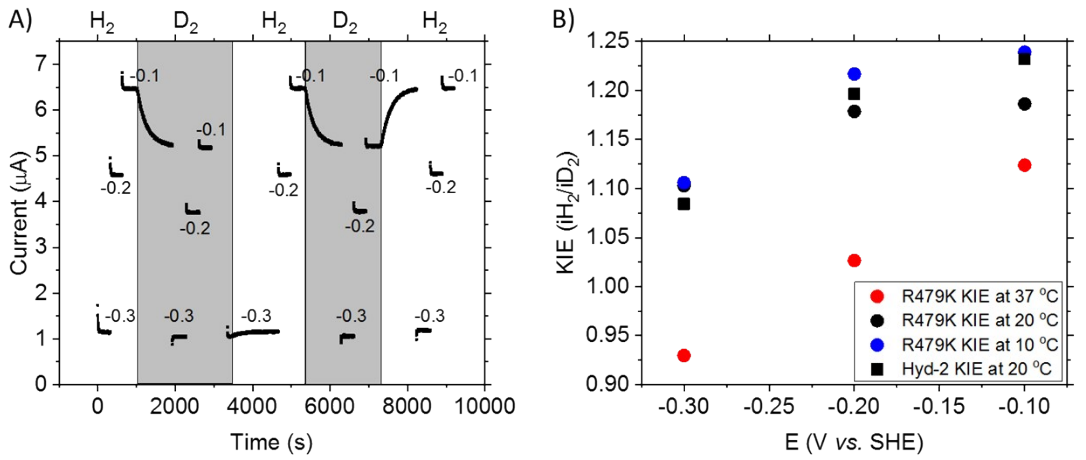

Figure S10: Determining the Kinetic Isotope Effect for H_2 oxidation in Hyd-2 and R479K. An example experiment is shown (A) for R479K at 10 °C and pH 6.0 where the potential is stepped between -0.3, -0.2 and -0.1 V as indicated in either 100% H_2 or 100% D_2. Data have been corrected for film loss. Other conditions: ω = 3000 rpm. (B) The trend for KIE as a function of temperature and potential.

Table S2. The temperature and potential dependences of KIE for H2 oxidation

Enzyme/ Temperature	KIE at -0.3 V	KIE at -0.2 V	KIE at -0.1 V
Hyd-2/20 °C	1.08	1.20	1.23
R479K/10 °C	1.11	1.22	1.24
R479K/20 °C	1.10	1.18	1.19
R479K/37 °C	0.93	1.03	1.12

Determination of the activation entropy difference ($\Delta\Delta S^{\ddagger}$) between Hyd-2 and R479K

The difference in the entropy of activation of Hyd-2 ($\Delta S_1^{\ddagger}$) and R479K ($\Delta S_2^{\ddagger}$) was calculated from the ratio of the turnover rates (k_1 and k_2, respectively) determined by conventional solution assay, and the enthalpies of activation ($\Delta H_1^{\ddagger}$and $\Delta H_2^{\ddagger}$, respectively) determined by PFE (Table 1). The examples given below are for the lower steady-state rate obtained for R479K in both H_2 oxidation and H^+ reduction assays as given in Table 1.

$\Delta\Delta S^{\ddagger}$ of H_2 oxidation

$$ln\left(\frac{k_1}{k_2}\right) = ln(k_1) - ln(k_2) = -\frac{\Delta H_1^{\ddagger}}{RT} + \frac{\Delta S_1^{\ddagger}}{R} + \frac{\Delta H_2^{\ddagger}}{RT} - \frac{\Delta S_2^{\ddagger}}{R} = \frac{\left(\Delta H_2^{\ddagger} - \Delta H_1^{\ddagger}\right)}{RT} + \frac{\left(\Delta S_1^{\ddagger} - \Delta S_2^{\ddagger}\right)}{R} = 3.8$$

multiplying through by R and T gives:

$$\left(\Delta H_2^{\ddagger} - \Delta H_1^{\ddagger}\right) + T\left(\Delta S_1^{\ddagger} - \Delta S_2^{\ddagger}\right) = 3.8\ RT$$

this rearranges to give:

$$\left(\Delta S_1^{\ddagger} - \Delta S_2^{\ddagger}\right) = \frac{(3.8\ RT) - \left(\Delta H_2^{\ddagger} - \Delta H_1^{\ddagger}\right)}{T} = \frac{9358 + 20700}{298} = 101\ J\ K^{-1}mol^{-1}$$

The value for $\Delta\Delta S^{\ddagger}$ can also be found by calculating the entropy of activation for Hyd-2 ($\Delta S_1^{\ddagger}$):

$$\Delta S_1^{\ddagger} = R\left(\ln k_1 - \left(\frac{lnk_BT}{h}\right) + \left(\frac{\Delta H_1^{\ddagger}}{RT}\right)\right)$$

$$\Delta S_1^{\ddagger} = 8.314\ (5.38 - (29.5) + (17.6)) = -53.6\ J\ K^{-1}mol^{-1}$$

and for R479K ($\Delta S_2^{\ddagger}$):

$$\Delta S_2^{\ddagger} = R\left(\ln k_2 - \left(\frac{lnk_BT}{h}\right) + \left(\frac{\Delta H_2^{\ddagger}}{RT}\right)\right)$$

$$\Delta S_2^{\ddagger} = 8.314\ (1.61 - (29.5) + (9.3)) = -154.4\ J\ K^{-1}mol^{-1}$$

The difference in the entropy of activation between Hyd-2 and R479K is then:

$$\Delta S_1^{\ddagger} - \Delta S_2^{\ddagger} = -53.6 + 154.4 = 101\ J\ K^{-1}mol^{-1}$$

$\Delta\Delta S^{\ddagger}$ of H^+ reduction

$$ln\left(\frac{k_1}{k_2}\right) = ln(k_1) - ln(k_2) = -\frac{\Delta H_1^{\ddagger}}{RT} + \frac{\Delta S_1^{\ddagger}}{R} + \frac{\Delta H_2^{\ddagger}}{RT} - \frac{\Delta S_2^{\ddagger}}{R} = \frac{\left(\Delta H_2^{\ddagger} - \Delta H_1^{\ddagger}\right)}{RT} + \frac{\left(\Delta S_1^{\ddagger} - \Delta S_2^{\ddagger}\right)}{R} = 5.5$$

multiplying through by R and T gives:

$$\left(\Delta H_2^{\ddagger} - \Delta H_1^{\ddagger}\right) + T\left(\Delta S_1^{\ddagger} - \Delta S_2^{\ddagger}\right) = 5.5\ RT$$

this rearranges to give:

$$\left(\Delta S_1^{\ddagger} - \Delta S_2^{\ddagger}\right) = \frac{(5.5\ RT) - \left(\Delta H_2^{\ddagger} - \Delta H_1^{\ddagger}\right)}{T} = \frac{13516 + 1053}{303} = 49\ J\,K^{-1} mol^{-1}$$

The value for $\Delta\Delta S^{\ddagger}$ can also be found by calculating the entropy of activation for Hyd-2 ($\Delta S_1^{\ddagger}$):

$$\Delta S_1^{\ddagger} = R\left(\ln k_1 - \left(\frac{lnk_B T}{h}\right) + \left(\frac{\Delta H_1^{\ddagger}}{RT}\right)\right)$$

$$\Delta S_1^{\ddagger} = 8.314\ (1.95 - (29.5) + (15.8)) = -\ 97.3\, J\,K^{-1} mol^{-1}$$

and for R479K ($\Delta S_2^{\ddagger}$):

$$\Delta S_2^{\ddagger} = R\left(\ln k_2 - \left(\frac{lnk_B T}{h}\right) + \left(\frac{\Delta H_2^{\ddagger}}{RT}\right)\right)$$

$$\Delta S_2^{\ddagger} = 8.314\ (-3.51 - (29.5) + (15.4)) = -\ 146.2\, J\,K^{-1} mol^{-1}$$

The difference in the entropy of activation between Hyd-2 and R479K is then:

$$\Delta S_1^{\ddagger} - \Delta S_2^{\ddagger} = \ -97.3 + 146.2 = 49\, J\,K^{-1} mol^{-1}$$

X-ray collection and refinement statistics

Table S3: X-ray data collection and refinement statistics for Hyd-2 R479K after various gas exposures

PDB code	6SYO	6SZD	6SYX	6SZK
Sample treatment	As-Isolated	Hydrogen reduced (flow)	Oxygen exposure after reduction	CO exposure after reduction
Space group	$P2_12_12_1$	$P2_12_12_1$	$P2_12_12_1$	$P2_12_12_1$
Unit cell dimensions (Å)	a=100.79, b=101.24, c=171.10	a=99.42, b=100.56, c=168.66	a=100.73, b=101.33, c=170.68	a=100.72, b=101.15, c=170.86
Resolution (Å)	86.8 – 1.25 (1.27-1.25)	100.6- 1.5 (1.53-1.50)	87.1-1.3 (1.32-1.30)	86.9-1.2 (1.22-1.2)
Total reflections	3.817,967 (155,819)	1,137,751 (54,830)	1,574,700 (76,634)	3,897,907 (181,806)
Unique reflections	425,274 (21,628)	262,942 (12,636)	390,605 (19,730)	539,529 (25,905)
Completeness (%)	99.3 (91.5)	97.8 (95.7)	92.1 (94.5)	99.8 (97.3)
Multiplicity	8.0 (7.2)	4.3 (4.3)	4.0 (3.9)	7.2 (7.0)
$\langle I/\sigma \rangle$	9.0 (1.1)	8.4 (1.1)	6.0 (1.2)	8.3 (1.2)
R_{merge} (%)	10.5 (164)	8.6 (109)	14.4 (89.8)	10.2 (140)
R_{pim} (%)	4.2 (70.0)	5.3 (67.0)	8.9 (58.3)	4.3 (58.9)
$CC_{1/2}$	0.998 (0.553)	0.997 (0.515)	0.986 (0.49)	0.982 (0.657)
Refinement				
R_{work}/R_{free} (%)	11.2/13.6	15.2/17.3	14.7/16.3	11.8/14.2
No. of atoms	14,623	13,881	14,539	13,881
Macromolecule	12,834	12,794	12,812	12,836
Solvent	1789	1087	1,727	1,454
Average B-factors				
Macromolecule	15.5	19.2	13.0	15.6
Solvent	28.4	26.7	26.9	26.8
RMSD bond lengths (Å)	0.018	0.012	0.015	0.018
RMSD angles (°)	2.0	1.70	1.92	2.0
Ramachandran plot favoured / outliers	96.3 % / 2 %	96.0 % / 1 %	96.0 % / 2 %	96.1 % / 2 %
Clashscore	0.75	1.3	0.75	0.63

Values in parenthesis refer to highest resolution shell

R509K vs R479K active site H-bonding

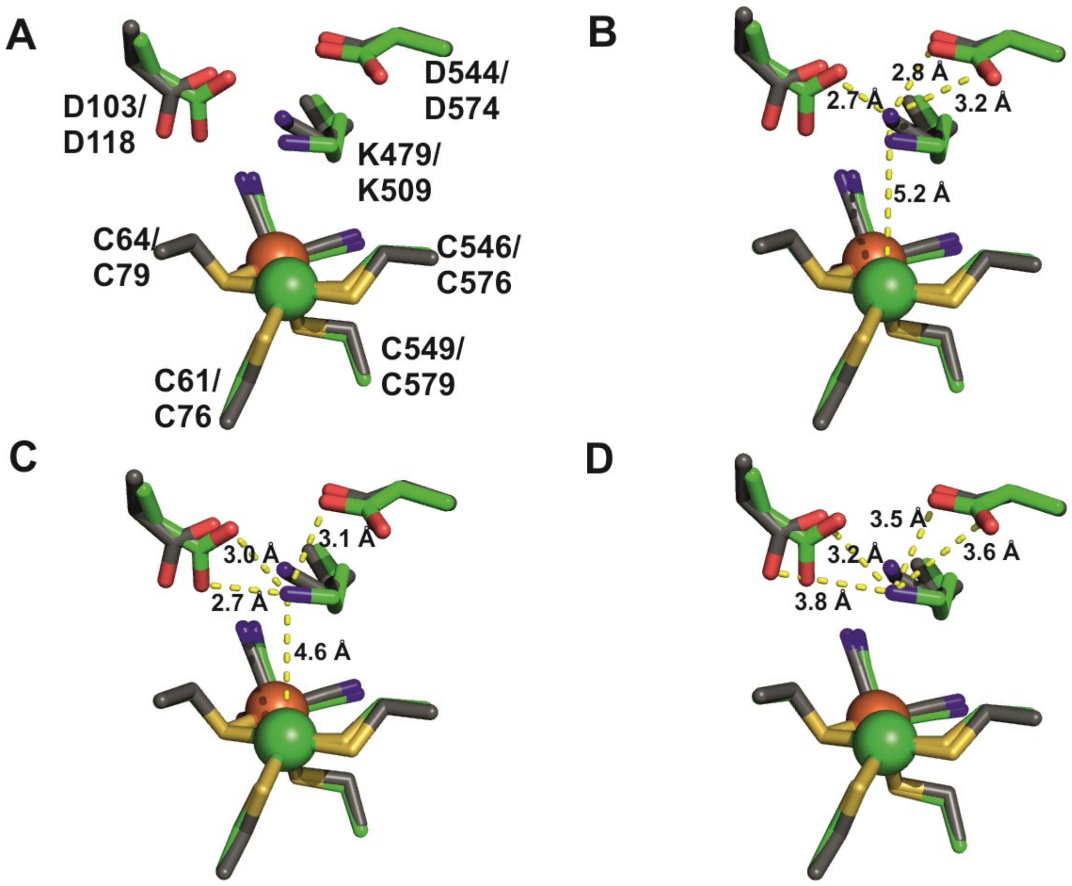

Figure S11: Comparison of the active sites of Hyd1 R509K and Hyd-2 R479K variants showing the hydrogen bonding interactions stabilising the position of the lysine side chain in each variant and demonstrating why K479 cannot adopt a similar orientation to K509. (A) Overlay of the active sites of Hyd1 R509K (green) and R479K (grey) highlights the different conformations adopted by the lysine side chain in the homologous proteins. (B) Hydrogen bond network stabilising K479. Comparison of the relative positions of the carboxylate groups of D103/D118 and D546/D547 show the expansion of the active site cavity in Hyd-2 relative to Hyd-1, resulting in a modified hydrogen bond network that stabilises the alternative conformation of K479 (C) Hydrogen bond network stabilising K509 (D) Distances between the amine group of K509 and the carboxylate groups of D103 and 546 show the active site of Hyd-2 would offer little to no stabilisation of the side chain in this conformation, resulting in a highly mobile residue.

Electron density distribution across diatomic ligand

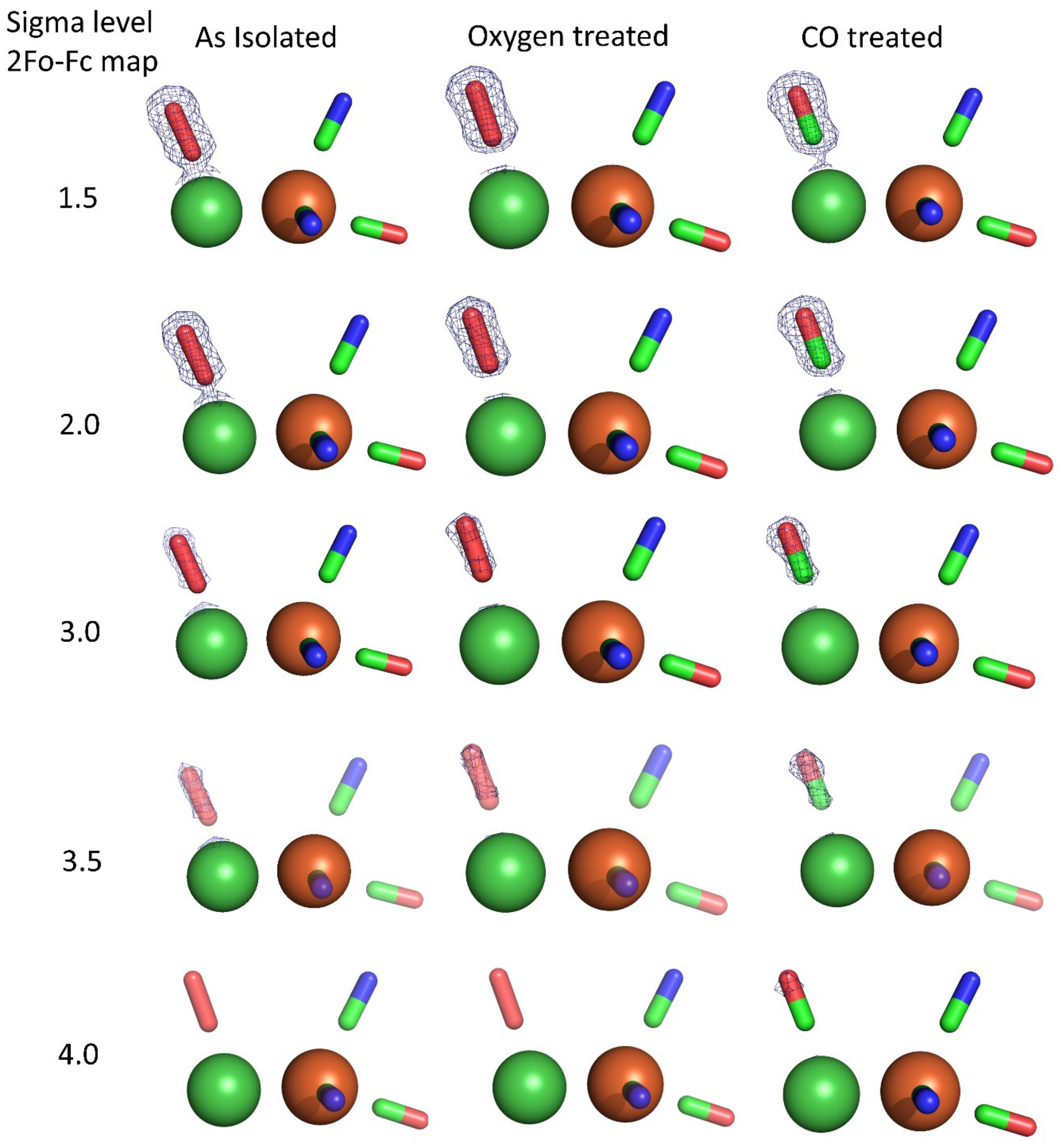

Figure S12: Electron density distribution across the diatomic ligand with 2Fo-Fc density maps contoured at increasing sigma levels (rmsd values for the electron density map) to highlight the symmetric distribution of electron density in the As-isolated and oxygen treated enzyme compared to the asymmetric distribution in CO treated enzyme. Numerical values for the electron density are summarised in Table S4.

Table S4: Absolute peak electron density values (and corresponding map rmsd) for each of the atoms in diatomic ligands bound at the active site indicate symmetric and asymmetric distribution of electron density across the X-Y and CO ligands respectively. Values for the endogenous CO ligand of the Fe atom are added for comparison. The higher density values for the endogenous CO are reflective of the higher occupancy of these atoms.

Enzyme treatment	Atom	Peak electron density (e/Å^3)
As-isolated	X	1.76 (3.6 σ)
	Y	1.76 (3.6 σ)
O2 exposure	X	1.85 (3.7 σ)
	Y	1.85 (3.7 σ)
CO exposure	C	1.86 (3.7 σ)
	O	2.22 (4.5 σ)
Endogenous CO	C	2.58 (5.4 σ)
	O	3.67 (7.6 σ)

Modelling the diatomic ligand in the active site

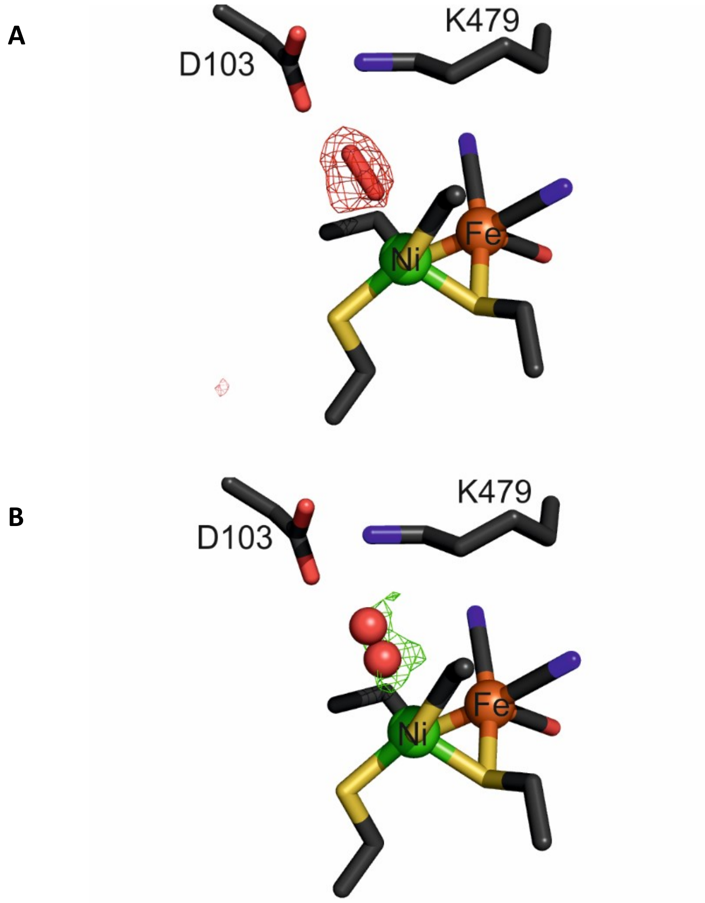

Figure S13: (A) Modelling the additional electron density observed adjacent to the Ni atom as a fully occupied oxygen molecule resulted in negative difference density in the Fo-Fc map (red mesh), indicating such a model overestimated the number of electrons in the ligand. (B) An alternative model for the nickel ligand was two partially occupied water molecules, (each with 50% occupancy). This produced Fo-Fc maps with positive difference density (green mesh) in the Fo-Fc maps showing this model underestimated the number of electrons. As a trial, modelling the density as an oxygen molecule with a occupancy of 80% removed all residual density from the Fo-Fc maps. Positive and negative maps are contoured at levels of +4 and -4 rmsd respectively.

R509K active site ordered water molecules

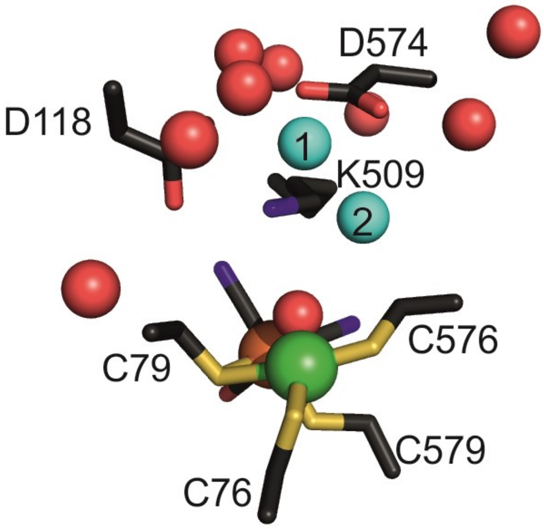

Figure S14: The active site of R509K variant of Hyd-1 displaying structurally ordered water molecules. Waters (red spheres) are positionally conserved between the active sites of Hyd-1 R509K and Hyd-2 R479K, including the additional, compared to the active site of the native enzyme, water molecules (cyan and labelled "1" and "2", see also Figure 2B) found in both variant proteins. These additional waters are not seen in either of the native enzymes.

R479K active site bonding involving gaseous entities

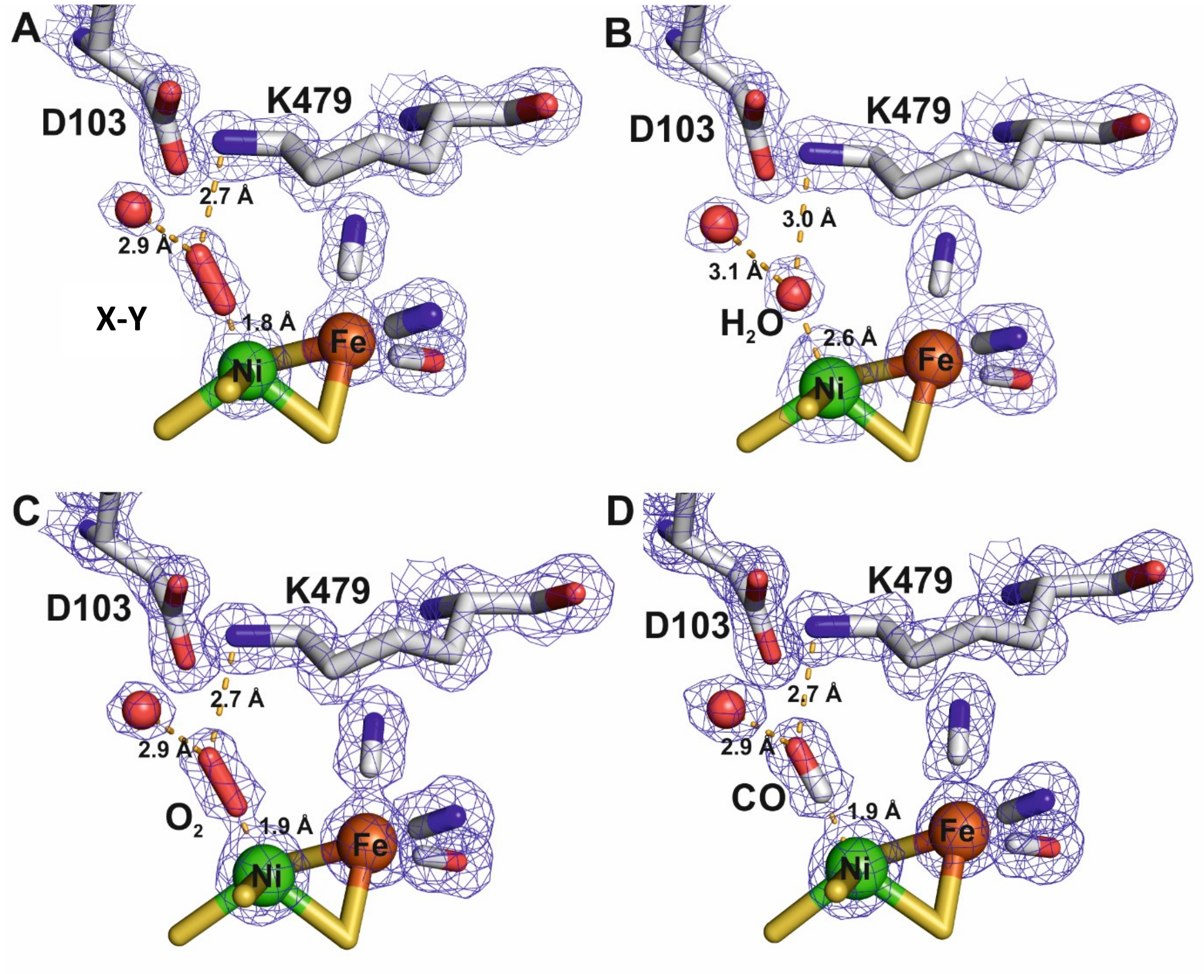

Figure S15: Hydrogen bonding interactions stabilising bound gas molecules in the active site of hyd-2 R479K with 2Fo-Fc electron density but with Fo-Fc omit density, included in figure 2, removed. (A) The as-Isolated enzyme shows the diatomic ligand coordinating the catalytic nickel atom. It is stabilised by hydrogen bonds to the side chain of K479 and an ordered water molecule (water '2' in Figure 1C). (B) The reduced enzyme replaces the oxygen species with a loosely coordinated water molecule (water '3' in Figure 1C), which forms two further hydrogen bonds to K479 and the same ordered water as the As-Isolated structure. After treatment of the enzyme with O_2 (C) or CO (D) the bound gas molecules coordinate the Ni atom and are further stabilised by interactions similar to those observed in the As-Isolated enzyme

Temperature factors of atoms in the Active site

Table S5: B-factors (Å^2) of components in the active site of Hyd-2 R479K after various treatments

Data set / residue	As-Isolated (1.3 Å)	Reduced (1.5 Å)	+ Oxygen (1.2 Å)	+ CO (1.2Å)
C61	10.1	13.5	7.5	9.9
C64	10.1	13.6	7.5	10.0
C546	11.2	15.1	8.7	11.1
C549	10.1	13.7	7.5	10.4
D103	12.4	15.7	9.8	11.7
K479	12.7	17.0	10.2	12.1
D544	12.1	16.2	9.9	11.6
$Fe(CN)_2(CO)$	10.2	13.3	7.8	10.4
Ni	11.8	14.3	8.2	10.6
Ni Ligand	15.8	27.3	13.5	12.3
H_2O "2"	23.8	27.5	20.7	21.5

Ni Ligand refers to the species coordinating to the Ni atom, H_2O "2" refers to the water molecule directly hydrogen bonded to the Ni-ligand.

Note on B-factors: The average B-factor for each component of the active site is shown, where $B=8\pi^2U^2$, and U corresponds to the positional r.m.s.d of the atoms about their equilibrium (i.e. modelled) position. The low B-factors show the positions of all modelled atoms are well defined, for example the atoms of the oxygen ligand in the As-isolated enzyme have a B-factor of 15.8 Å^2 corresponding to a U-value of 0.45 Å, much less than the Van-de-Waals radius of an oxygen atom (1.55 Å).

Transmission IR spectra of R479K in the νCN region

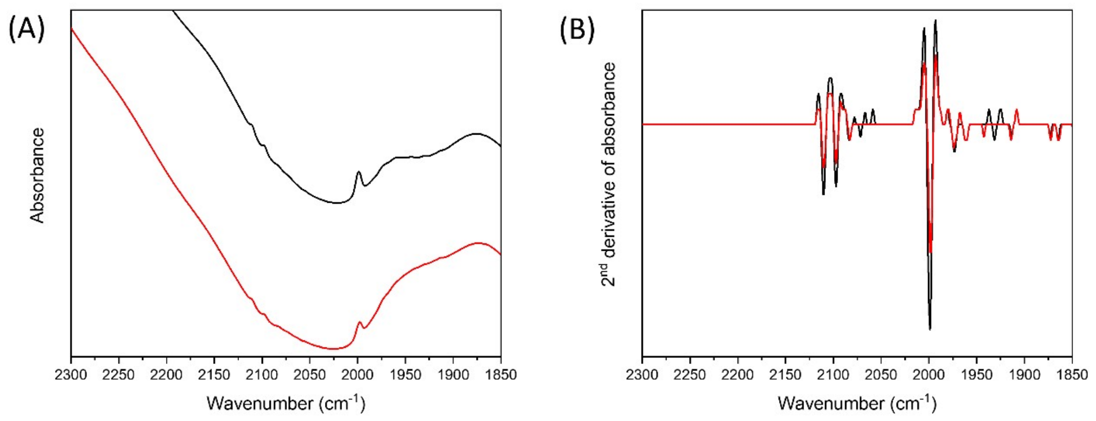

Figure S16: Cyanide region of the solution-based transmission IR spectra of anaerobically oxidised R479K (A) Raw data of R479K that has been H_2-reduced then anaerobically oxidised with DCIP corresponding to the data from Figure 13 (raw data corresponding to Figure 13E, oxidised with DCIP under strict anaerobic conditions after being H_2-reduced) is shown in black, raw data corresponding to Figure 13F (H_2-reduced, left overnight and then oxidised with DCIP prepared the previous day) is shown in red, and (B) the 2nd derivatives of the same data. It can be clearly seen that there are no distinguishable IR bands in the high wavenumber region beyond 2112 cm^{-1}.

IR spectra of poised R479K crystals before and after CO exposure

A

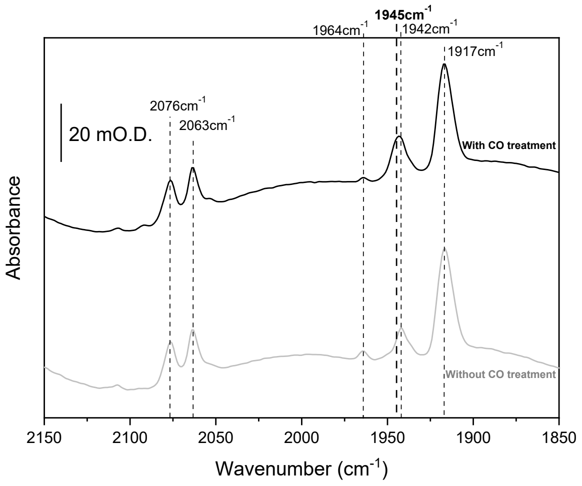

B

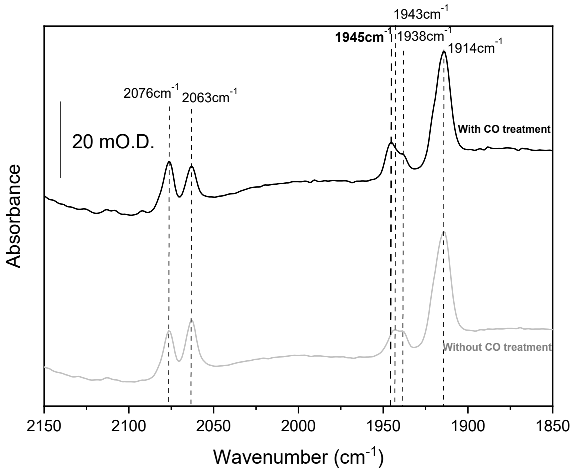

C

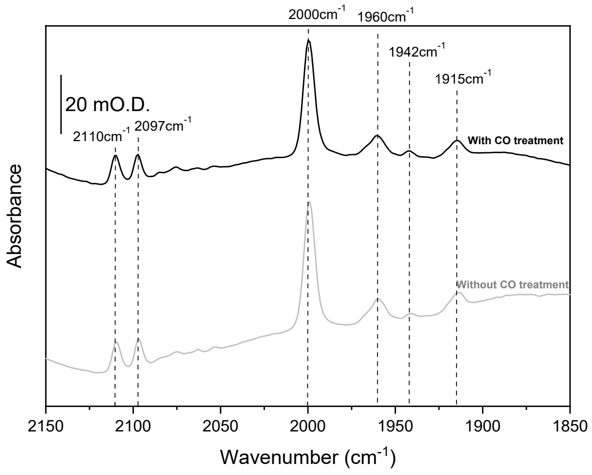

Figure S17: IR spectra collected from single crystals of R479K in crystallisation buffer at pH 5.9. Crystals were electrochemically poised, *in situ*, and then treated with CO (black line) or left unexposed (grey

line). Applied potentials were (a) −700 mV; (b) −150 mV; (c) +300 mV. Prior to these measurements, all crystals were reduced with H_2 for 24 hours.

Crystals of R479K Hyd-2 were electrochemically poised in crystallisation buffer containing redox mediators.[8] IR spectra were recorded in an anaerobic glove box using an IR microscope in a transmission cell between CaF_2 windows.

Although the IR spectra for crystals of R479K are difficult to assign on the basis of known, IR-characterised states of native Hyd-2,[9,10] or other native [NiFe]-hydrogenases,[11] it is clear that CO exposure to crystals of R479K at reducing potentials (-700 mV, Figure S17A) does not give rise to the 'high wavenumber species' with ν_{CO} at 2000 cm^{-1}.

A potential of -150 mV was then applied (Figure S17B). For the native enzyme, this would favour the Ni_a-SI state (ν_{CO} 1945 cm^{-1}),[9,10] which is readily poisoned by CO to give the NiS-CO inhibited state with ν_{CO} for the endogenous carbonyl ligand shifted only very slightly to 1944 cm^{-1}, and a new band for the exogenous, inhibitory CO on Ni(II) at 2054 cm^{-1}.[9] The R479K variant shows only a minority species with ν_{CO} close to 1945 cm^{-1} when crystals are poised at -150 mV, indicating that Ni_a-SI does not accumulate significantly for this variant. Exposing crystals poised at -150 mV to CO results in a change in intensities around 1945 cm^{-1} (to 1943cm^{-1}): it is not possible to assign a band for an exogenous CO, although this could be hidden under the ν_{CN} bands. Again, it is clear that there is no absorption band at 2000 cm^{-1}, and therefore CO exposure at this potential does not give rise to the high wavenumber species.

Poising crystals at +300 mV (under anaerobic conditions, Figure S17C) with no CO exposure gives rise to a spectrum in which the dominant state has a ν_{CO} band at 2000 cm^{-1} and corresponding ν_{CN} bands appear at 2110 and 2097 cm^{-1}. This is consistent with the solution spectrum for anaerobically-oxidised R479K (Figure 13), although the improved signal/noise arising from a high effective protein concentration in the crystals also reveals minor contributions from other states at lower wavenumber. No change is observed in the spectrum for crystals poised at +300 mV when CO is introduced, confirming that the 'high wavenumber species' is insensitive to CO inhibition.

Table S6. Infrared band positions (cm^{-1}) of the active site states of selected [NiFe]-hydrogenases in their various active redox states (subscript 'a') and the oxidised inactive state Ni-B. The Ni-SI_r state represents an inactive state during the activation of Ni-B to Ni_a-SI and is not thought to be involved in the active catalytic cycle.[12] Note that the sub-states of Ni_a-L and Ni_a-R are presented in descending order, i.e. sub-state I at highest energy, III at lowest as per reference [13].

**Enzyme**	**Ni-B**	**Ni_a-SI**	**Ni-SI_r**	**Ni_a-C**	**Ni_a-L_I**	**Ni_a-L_{II}**	**Ni_a-L_{III}**	**Ni_a-R_I**	**Ni_a-R_{II}**	**Ni_a-R_{III}**
D. vulgaris MF	1955	1943	1922	1961		1911	1890	1948	1932	1919
Hyd-2	*1957*	*1945*	*1937*	*1964*						

D. vulgaris MF data from references [14–17]; previously reported Hyd-2 IR bands[10]; R479K data is presented in Figure 13.

IR spectra of electrochemical redox titrations of R479K single crystals

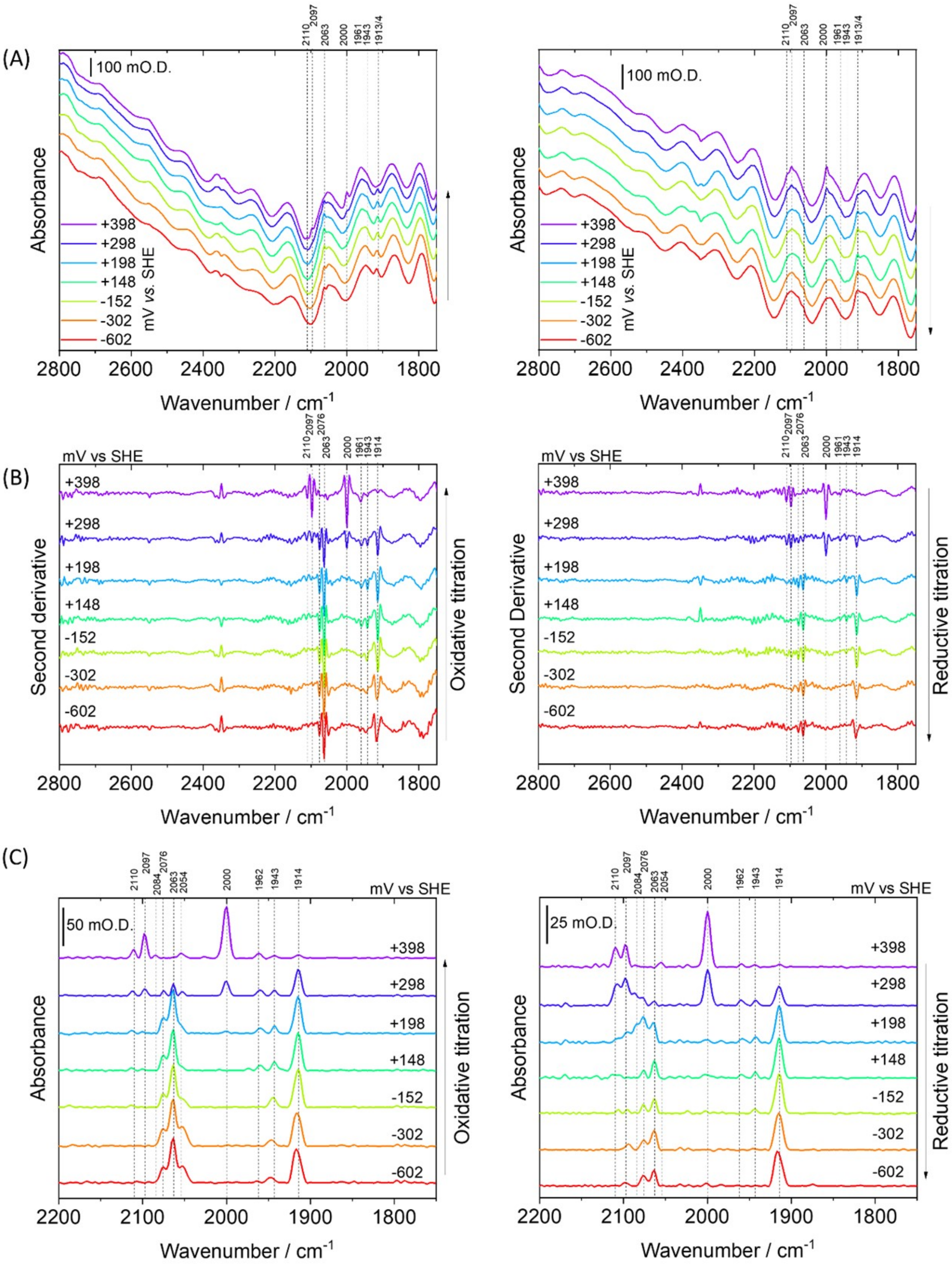

Figure S18: Single crystal redox titrations of R479K (oxidative left-hand side, reductive right-hand side) at pH 6.0 between -600 to +400 mV showing the reversible formation of the high wavenumber species with ν_{CO} at 2000 cm^{-1} at potentials above +148 mV. Data is presented to across an extended wavenumber range (2800-1750 cm^{-1}) as raw absorbance data (A) and its second derivative (B) and at a narrower wavenumber range (2200-1750 cm^{-1}) as baseline-subtracted (C).

The microspectroscopic-electrochemical cell[8] was set up anaerobically in a glovebox using H_2-reduced crystals of R479K in presence of crystal stabilisation buffer (100 mM Bis-tris pH 5.9, 200 mM $MgCl_2$, 23% w/v PEG 3350) containing 0.5 mM each of mediators Redox mediator 2,6- dichloroindophenol sodium salt, phenazine methosulfate, indigo carmine, anthraquinone-2-sulfonate, and methyl viologen, and was sealed in the anaerobic environment of the glovebox (<20 ppm O_2) before being transferred to the beamline.

Reductive activation of single crystals of R479K in the microspectroscopic-electrochemical cell was performed at -600 mV for until no further changes were observed in the ν_{CO} and ν_{CN} bands in the IR spectra over a period of approximately 10 min. The oxidative titration was then performed from -600 mV to +400 mV in 25-100 mV steps, only stepping the potential once no further changes were observed in the ν_{CO} and ν_{CN} bands in the IR spectra over a period of approximately 10 min. The potential was held constant at +400 mV whilst the microscope stage was moved to be able to sample a different area of the crystal for the reductive titration. The potential was then lowered in the reductive direction from +400 to -600 mV at the same potential steps as previously used for the oxidative titration.

Theoretical Methods used for Analysis of Hyd-2 R479K Structures

Setting up the QM/MM model

The model was set up using the crystal structure of the 'as-isolated' state (PDB-ID: 6SYO). The protein contains 11 peptide chains labelled: A, B, C, D, E, F, L, M, S, T, W. Residues with multiple occupancies for atoms were inspected with respect to their hydrogen bond network. Occupancy A was chosen for the following residues: ILE-S-12 (S being the chain name), ASN-S-61, LYS-S-173, GLU-L-22, SER-L-112, SER-L-292, GLN-L-395, SER-L-444, GLU-T-206, GLU-M-22, VAL-M-86, SER-M-330, ASP-M-437, SER-M-444. Occupancy B was selected for GLU-S-206, LYS-L-46, GLU-L-196, ARG-M-51, SER-M-112, GLN-M-395. Glycerol molecules present in the crystal structure surrounding the protein were deleted. Protonation states of titratable amino acid sidechains were determined by manual inspection based on hydrogen bond networks. Histidine residues determined to be N_{ε}-protonated were: HIS-S/T-47 (S/T means chain S and T), HIS-S/T-56, HIS-T-63, HIS-S/T-100, HIS-S/T-189, HIS-S/T191, HIS-S/T-197, HIS-S/T-212, HIS-L/M-16, HIS-L/M-68, HIS-L/M-99, HIS-L/M-107, HIS-L/M-174, HIS-L/M-214, HIS-L/M-318, HIS-L/M-419, HIS-L/M-484. Histidine residues with a proton on N_{δ} were: HIS-S-63, HIS-S/T-156, HIS-S/T-164, HIS-S/T-220, HIS-S/T-250, HIS-S/T-263, HIS-S/T-267, HIS-L/M-102, HIS-L/M-104, HIS-L/M-141, HIS-L-190, HIS-L/M-431, HIS-L/M-455, HIS-L/M-552. Doubly protonated histidine residues are: HIS-S/T-31, HIS-M-190, HIS-L/M-333, HIS-L/M-541. All aspartic acid residues and glutamic acid residues were modelled as deprotonated except ASP-S-199 which was protonated on the OD2 atom. All lysine and arginine residues were modelled as protonated. Cysteines coordinated with metals, Ni, Fe, and Mg, were modelled as deprotonated. The inorganic residues: [3Fe-4S] cluster, [3Fe-4S] cluster, and the NiFe-cluster were modelled using a nonbonded model. Hirshfeld population analysis of a DFT-CPCM calculation was used to derive atomic charges while CHARMM Lennard-Jones parameters were used for the sulfides.

The GROMACS program version 2018.3[6] was used to add missing hydrogen atoms to the model. A cubic box was created with 10 Å between the protein and the edge of the box. Box dimension were 72.09, 78.39, 116.10 Å (length, width, height). The box was filled with 78940 solvent molecules (TIP3 solvent model). The system was neutralized via the addition of 48 Na^+ ions. The full system size was 267049 atoms. Following system setup, a classical minimization was performed to relax H-atom positions with metal-coordinated waters and waters close to the active site frozen. Classical NVT simulations were performed for 2.5 ns with all metal clusters constrained ([3Fe-4S] cluster, [3Fe-4S] cluster, [NiFe-CO(CN^-)$_2$X$_2$] and Mg-complex) as well as LYS-479 and the water molecule close to the unknown X_2 ligand bound to Ni. Following the MD simulations, QM/MM as well as QM-cluster models were prepared. For QM/MM geometry optimizations, an active region of 1120 atoms (an approximately 11 Å radius sphere around one of the Ni ion) was defined. Multiple QM-cluster models and QM-regions in the QM/MM calculations were defined and tested. The smallest QM-cluster calculation contained only the NiFe-cluster and the sidechains (SMe groups) of CYS-61, CYS-64, CYS-546, and CYS-549. The smallest QM-region QM/MM model contained in addition to the NiFe-cluster and the 4 cysteine residues, LYS-479 and a water molecule in hydrogen-bonding distance with the diatomic ligand. A large QM-region QM/MM model (150 atoms) was also defined, containing VAL-63, ASP-103, ASP-544, GLU-14, SER-502, HIS-99, HIS-104, and three water molecules, shown in Figure S19.

Vibrational frequency calculations

Harmonic vibrational frequencies were calculated. Both unscaled frequencies as well as frequencies scaled by a factor of 0.9781 are reported. This scaling factor was derived from a calculation of free CO at the same level of theory compared to the experimental frequency.

Calculations of O_2 ligand models.

An O_2 ligand bound to Ni(II) was initially considered. Binding an O_2 ligand to Ni(II) as a terminal ligand lead to spontaneous superoxide (O_2^-) formation in both QM/MM and QM-cluster model calculations, the electronic structure best described as an open-shell Ni(III) ion spin-coupled to an O_2^- ligand (note:

this state is still labelled as Ni(II)-O_2). The open-shell singlet state calculated was revealed to be a spin-coupled according to analysis of the electronic structure (spin density, Hirshfeld spin populations and Pipek-Mezey localized orbital analysis). This electronic structure was accompanied by bending of the O_2 ligand relative to the Ni ion (a Ni-O-O angle of ~125°), consistent with superoxide formation but clearly inconsistent with the more linear Ni-X-X angle (163°) found in the X-ray structure. Figure S20 and Figure S23 show results for other redox states of the Ni ion (Ni(II), Ni(III), Ni(I)), and multiple spin states of the NiFe cofactor however, all calculated possibilities resulted consistently in a calculated Ni-O-O angle of ~120-126°.

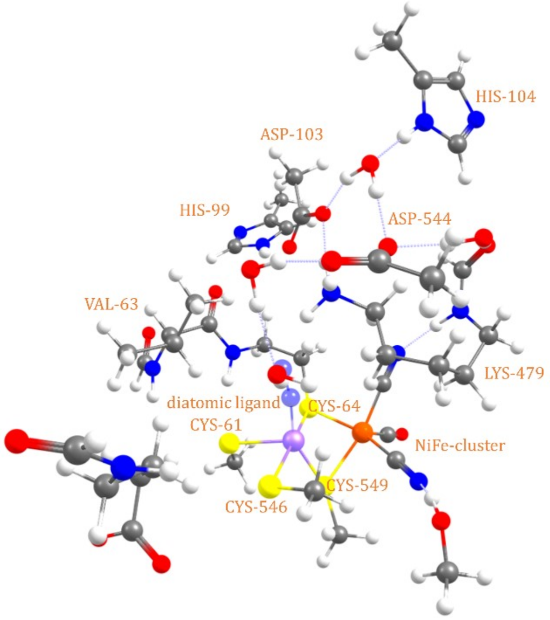

Figure S19: The largest QM-region (150 atoms) considered in the QM/MM calculations with accompanying link atoms. Other atoms described at the MM level.

QM/MM results for different ligands bound to R479K

Results of QM/MM calculations with different diatomic ligand possibilities can be found in Figure S20 (calculated Ni-X-X angles), Figure S21 and Table S6 (vibrational frequencies) and Figure S22 (active site heavy-atom distances surrounding the NiFe cofactor).

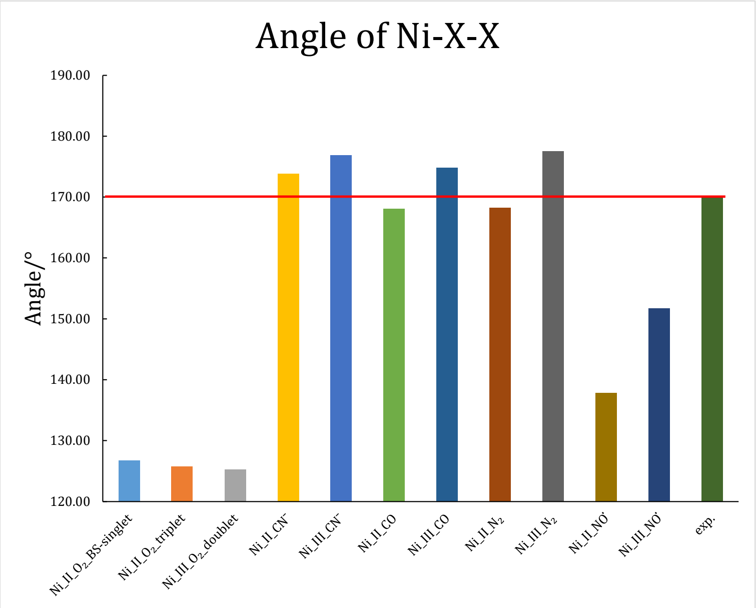

Figure S20: The Ni-X-X angle for different models of the unknown ligand according to QM/MM calculations with a small QM-region. The red line refers to the X-ray structure ("as isolated" state).

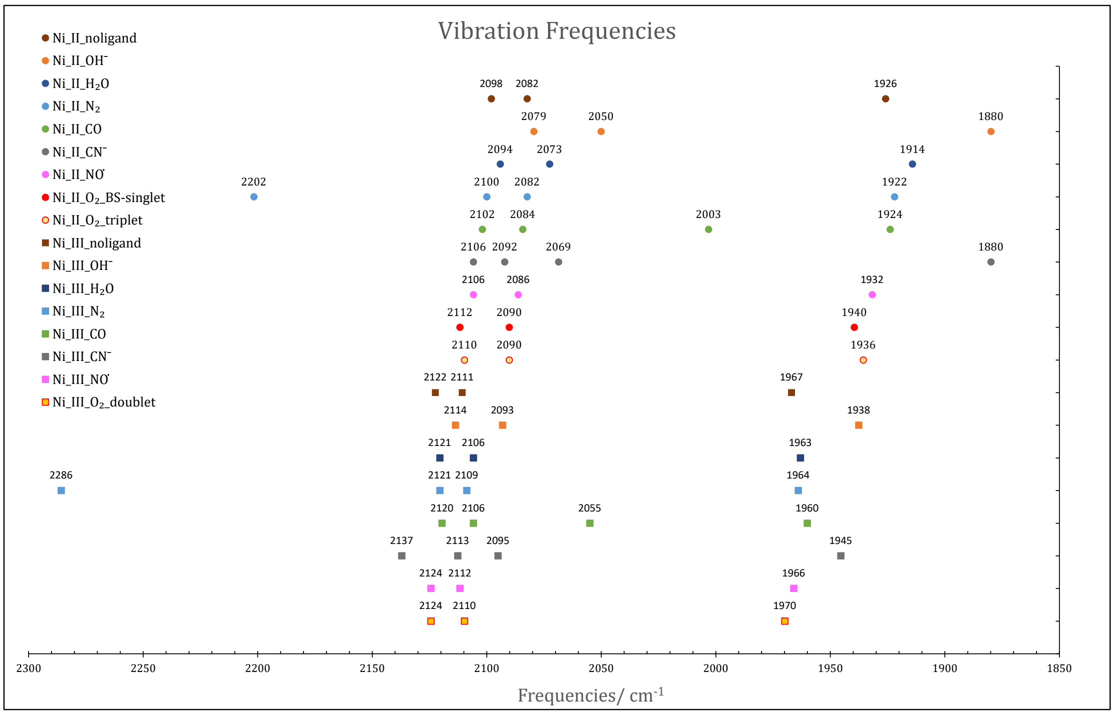

Figure S21: The calculated vibrational frequencies of QM/MM models (small QM-region) with different redox states and different possibilities for the ligand coordinating to Ni. All calculated harmonic vibrational frequencies were scaled by the factor 0.9781.

Table S6. The calculated vibrational frequencies from Figure S21, both unscaled and scaled (using a factor of 0.9781) and additional models from Figure S26. Models are labelled according to formal redox state (assuming metal-based oxidation)

Formal redox state	Ligand	Total spin (S)	Calc. v (cm^{-1}) unscaled	Calc. v (cm^{-1}) scaled by 0.9781
Ni(II)-Fe(II)	none	0	2145,2129,1969,	2098,2082,1926
Ni(II)-Fe(II)	OH^-	0	2126,2096,1922,	2079,2050,1880
Ni(II)-Fe(II)	H_2O	0	2141,2119,1957,	2094,2073,1914
Ni(II)-Fe(II)	N_2	0	2251,2147,2129,1965	2202,2100,2082,1922
Ni(II)-Fe(II)	CO	0	2149,2131,2048,1967	2102,2084,2003,1924
Ni(II)-Fe(II)	CN^-	0	2153,2139,2115,1922	2106,2092,2069,1880
Ni(II)-Fe(II)	NO	0.5	2153,2133,1975,	2106,2086,1932
Ni(II)-Fe(II)	O_2	1	2159,2137,1983,	2112,2090,1940
Ni(II)-Fe(II)	O_2	0	2157,2137,1979,	2110,2090,1936
Ni(III)-Fe(II)	none	0.5	2170,2158,2011,	2122,2111,1967
Ni(III)-Fe(II)	OH^-	0.5	2161,2140,1981,	2114,2093,1938
Ni(III)-Fe(II)	H_2O	0.5	2168,2153,2007,	2121,2106,1963
Ni(III)-Fe(II)	N_2	0.5	2337,2168,2156,2008	2286,2121,2109,1964
Ni(III)-Fe(II)	CO	0.5	2167,2153,2101,2004	2120,2106,2055,1960
Ni(III)-Fe(II)	CN^-	0.5	2185,2160,2142,1989	2137,2113,2095,1945
Ni(III)-Fe(II)	NO	0	2172,2159,2010,	2124,2112,1966
Ni(III)-Fe(II)	O_2	0.5	2172,2157,2014,	2124,2110,1970

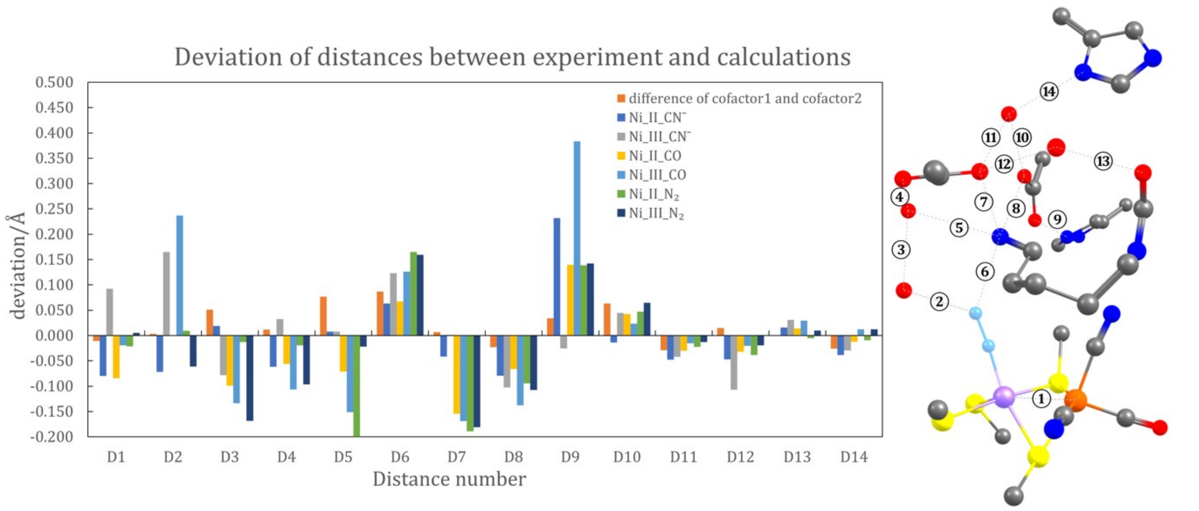

Figure S22: Deviations of some heavy-atom distances for the large QM-region. QM/MM calculations with CN^-, CO, or N_2 as the diatomic ligand in two redox states (Ni^{III}-Fe^{II}, and Ni^{II}_Fe^{II}). The inset shows the defined distances.

Results of the QM-cluster and QM/MM models with a putative O_2-bound ligand

Many calculations (using r^2SCAN functional) were performed with a putative O_2 ligand, varying the redox state and spin state of the system as shown in Figure S23. The Ni-O-O angle was consistently found to be between 120-130°.

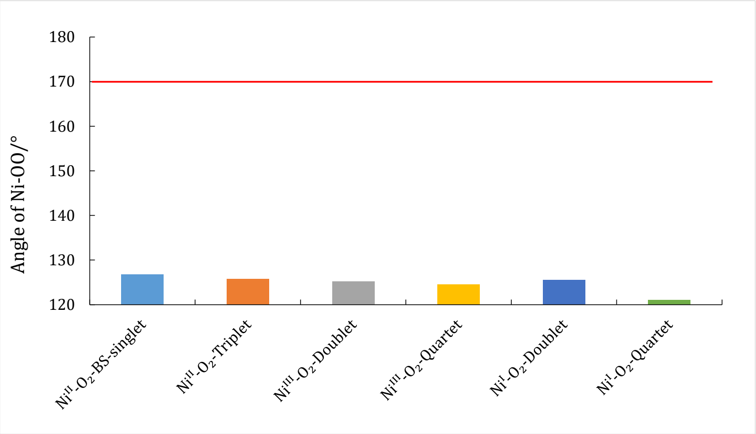

Figure S23: The calculated angle of Ni-O-O of the Ni(II)-O_2 model in multiple redox/spin states.

Multiple density functional theory methods (r^2SCAN, TPSS, TPSSh, B3LYP, PBE0 and BHLYP) were also tested for describing the electronic structure of the putative Ni(II)-O_2 model in a broken-symmetry singlet, restricted singlet and triplet states. The functional dependency on the molecular structure is shown in Figure S24; the results reveal that the Ni-Fe distance is rather sensitive to the details of the electronic structure exhibited by each functional (specifically the amount of HF exchange) while the Ni-O-O angle was again found to be consistently between 120-130°.

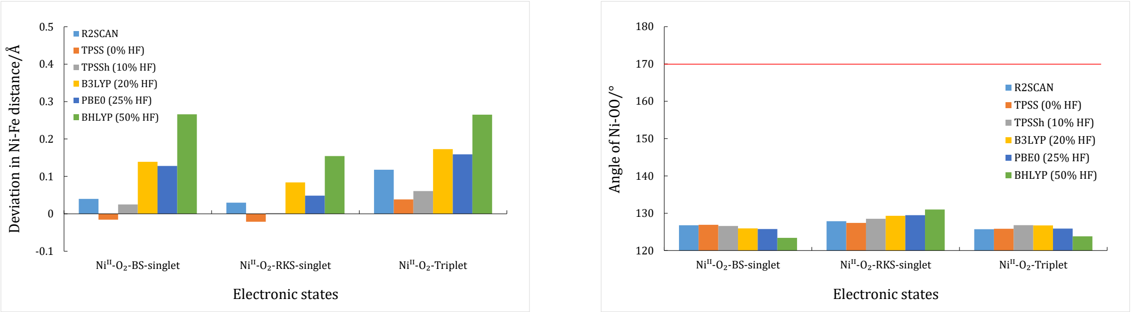

Figure S24: left: The deviation (Å) of the Ni-Fe distance of a Ni(II)-O_2 model (w.r.t. X-ray structure) with different functionals. right: The Ni-O-O angle in the Ni(II)-O_2 model calculated with different density functionals. The red line refers to the experimental value.

To investigate the effect of the protein environment, QM-cluster models with different surrounding residues included in the model and QM/MM models with different QM-regions were explored for the Ni(II)-O_2 model (see Figure S25). While the Ni-Fe distance showed some small sensitivity with respect

to QM-cluster definition or QM-region composition, the Ni-O-O angle was barely affected by the protein environment around the Ni-Fe cluster.

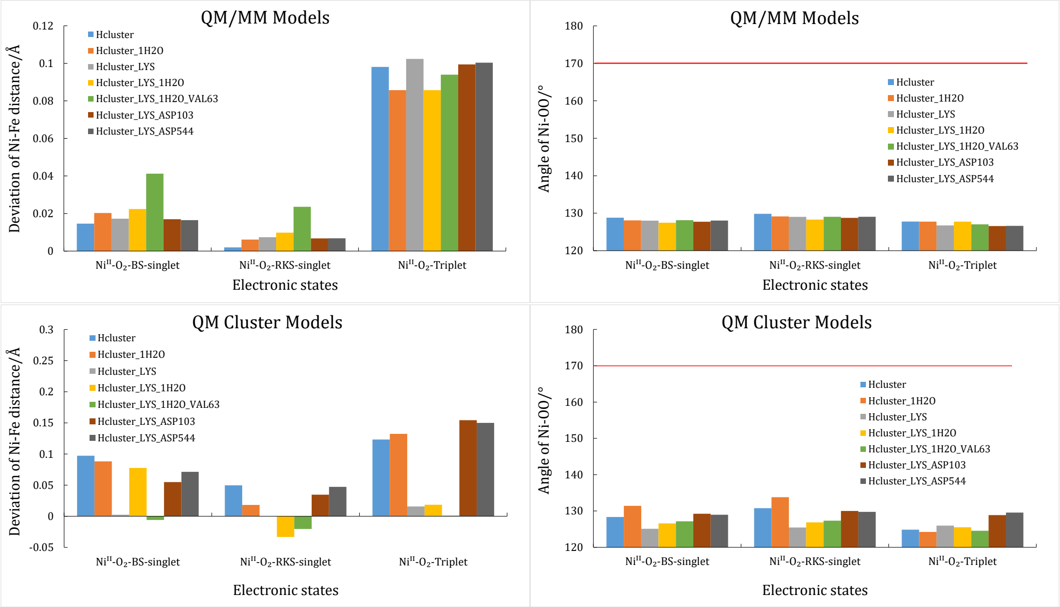

Figure S25: Comparing the deviation of Ni-Fe and angles of Ni-O-O among different sizes of the QM region in QM/MM models and QM cluster models. The red line refers to the experimental value. LYS refers to the LYS-479 residue. 1H_2O refers to the water molecule making a hydrogen-bonding interaction with X_2.

Results of models with even more oxidized forms

Calculations were also performed on even more oxidized models of the NiFe active site for further exploration of possible near-linear O-O forms or Fe oxidation to Fe(III) which could explain the presence of a strongly shifted CO vibrational mode to ~2000 cm^{-1}.

i) Further oxidation of the Ni ion beyond the Ni(III)-Fe(II) state would lead to a Ni(IV) ion which could conceivably stabilize a Ni(IV)-peroxo form. We explored this by removing either 1 or 2 electrons from the Ni(II)-Fe(II) redox state with O_2 added as a ligand in an initially linear geometry. As shown in Figure S26, the O_2 ligand remains bent (Ni-O-O angle of ~124°), consistent with superoxide character, as also seen by spin density on the oxygen atoms. The spin density around Ni is consistent with the presence of a S=1/2 Ni(III) ion. Additional spin density could be seen associated with the S-atoms across one or more thiolate ligands suggesting that oxidation has occurred on the S-atoms.

ii) Oxidation of the Fe ion from Fe(II) to Fe(III) could explain a strongly shifted CO frequency. An oxidized NiFe hydrogenase from *H. thermoluteolus* featuring a Ni-bound glutamate residue and a bridging cysteine residue (previously Ni-bound) had initially been assigned as a Ni(IV)-Fe(II) state but a recent computational study (Kumar et al. JACS, 2023, 145, 10954-10959) has suggested an antiferromagnetically coupled Ni(III)-Fe(III) state as an alternative possibility. That state also features a strongly shifted CO band at 1993 cm^{-1} (similar to the experimental 2000 cm^{-1} band in this work). A Ni(III)-Fe(III) redox state in the O_2 calculations shown in Figure S26 was

not revealed as only marginal spin density is associated with the Fe. We further explored the possibility of a Ni(III)-Fe(III) state with a CN^- ligand present (and 2 electrons removed). As the Ni(III)-Fe(III) scenario is likely to arise via antiferromagnetic spin coupling we explored calculations of both a triplet high-spin solution and an antiferromagnetic broken-symmetry singlet solution (found by flipping the spin on either Ni or Fe). Calculations were tested with both the r^2SCAN functional and the hybrid PBE0 functional (as the HF exchange could help stabilizing spin-coupled solutions). However, as the spin density plots reveal, there is no sign of Fe oxidation in these models either, with only marginal spin density is present around Fe and instead S-based oxidation occurs instead.

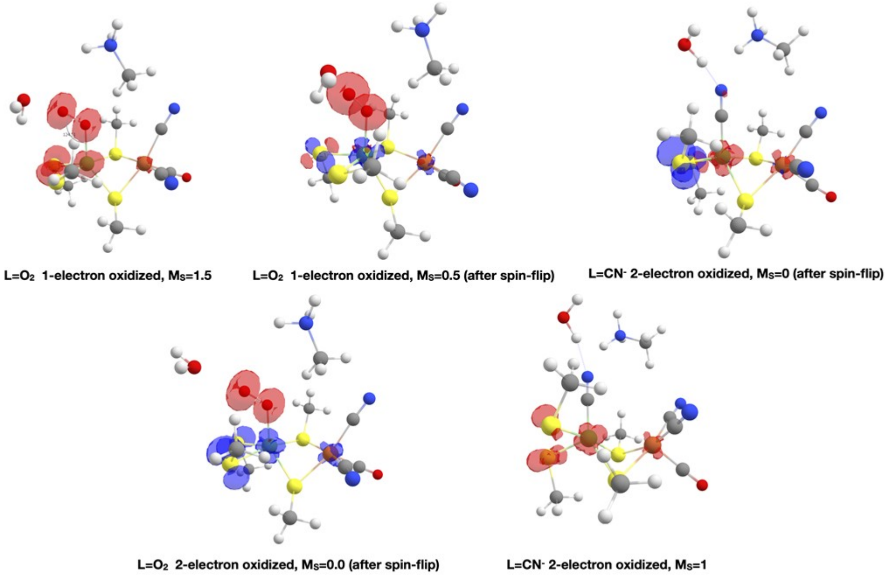

Figure S26: Optimized geometries (QM-region shown) and spin density plots (isovalue=0.01) of 1 and 2-electron oxidized models with either O_2 or CN^- as ligand. Red color indicates alpha-spin while blue indicated beta-spin.

Diatomic ligand bound to pocket next to active site Ni atom

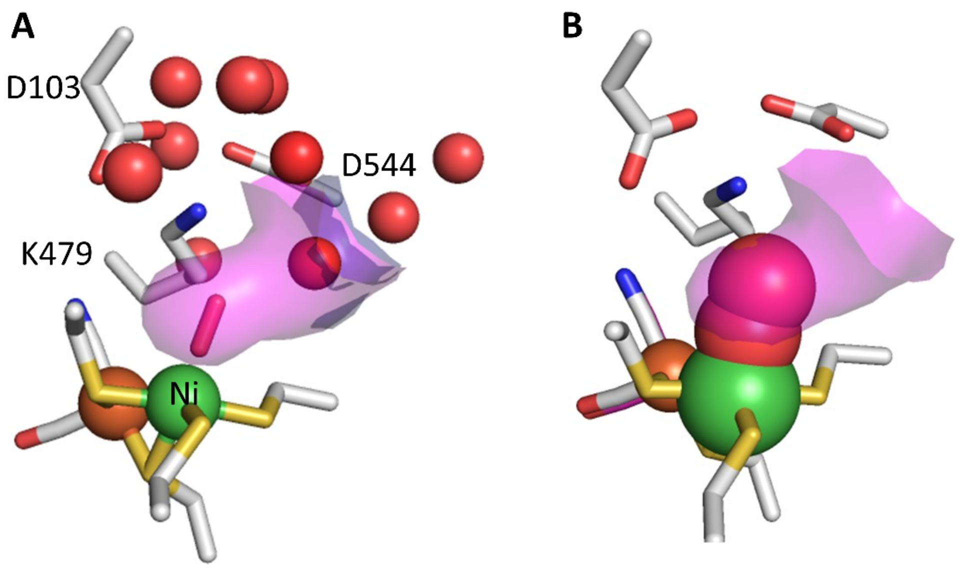

Figure S27: Extension of the active site cavity containing the diatomic ligand (A) Diatomic shown in stick view shows the location of the ligand at the terminus of the cavity. (B) Displaying ligand and Ni atoms with appropriate Van der Waals radii (O=152 pm, Ni=163pm) demonstrates the snug fit of the ligand within the cavity.

Supplementary References

1 S. E. Beaton, R. M. Evans, A. J. Finney, C. M. Lamont, F. A. Armstrong, F. Sargent and S. B. Carr, *Biochemical Journal*, 2018, **475**, 1353–1370.

2 R. M. Evans, E. J. Brooke, S. A. M. Wehlin, E. Nomerotskaia, F. Sargent, S. B. Carr, S. E. V. Phillips and F. A. Armstrong, *Nature Chemical Biology*, 2016, **12**, 46–50.

3 K. A. Vincent, A. Parkin and F. A. Armstrong, *Chem Rev*, 2007, **107**, 4366–4413.

4 F. A. Armstrong, R. M. Evans, S. V. Hexter, B. J. Murphy, M. M. Roessler and P. Wulff, *Acc Chem Res*, 2016, **49**, 884–892.

5 S. Krishnan and F. A. Armstrong, *Chem Sci*, 2012, **3**, 1015–1023.

6 O. Rüdiger, J. M. Abad, E. C. Hatchikian, V. M. Fernandez and A. L. De Lacey, *J Am Chem Soc*, 2005, **127**, 16008–16009.

7 E. J. Brooke, R. M. Evans, S. T. A. Islam, G. M. Roberts, S. A. M. Wehlin, S. B. Carr, S. E. V Phillips and F. A. Armstrong, *Biochemistry*, 2017, **56**, 132–142.

8 P. A. Ash, S. E. T. Kendall-Price, R. M. Evans, S. B. Carr, A. R. Brasnett, S. Morra, J. S. Rowbotham, R. Hidalgo, A. J. Healy, G. Cinque, M. D. Frogley, F. A. Armstrong and K. A. Vincent, *Chem Sci*, 2021, **12**, 12959–12970.

9 M. Senger, K. Laun, B. Soboh and S. T. Stripp, *Catalysts*, 2018, **8**, 1–14.

10 S. V. Hexter, M. W. Chung, K. A. Vincent and F. A. Armstrong, *Journal of the American Chemical Society*, 2014, **136**, 10470–10477.

11 P. A. Ash, R. Hidalgo and K. A. Vincent, *ACS Catalysis*, 2017, **7**, 2471–2485.

12 W. Lubitz, H. Ogata, O. Rüdiger and E. Reijerse, *Chem Rev*, 2014, **114**, 4081–4148.

13 P. A. Ash, S. E. T. Kendall-Price, R. M. Evans, S. B. Carr, A. Brasnett, S. Morra, R. Hidalgo, A. J. Healy, G. Cinque, M. D. Frogley, F. A. Armstrong and K. A. Vincent, *Chem. Sci.*, , DOI:10.1039/D1SC01734A.

14 C. Fichtner, C. Laurich, E. Bothe and W. Lubitz, *Biochemistry*, 2006, **45**, 9706–9716.

15 P. Kellers, M. E. Pandelia, L. J. Currell, H. Görner and W. Lubitz, *Physical Chemistry Chemical Physics*, 2009, **11**, 8680–8683.

16 M. E. Pandelia, H. Ogata, L. J. Currell, M. Flores and W. Lubitz, *Journal of Biological Inorganic Chemistry*, 2009, **14**, 1227–1241.

17 M. E. Pandelia, H. Ogata, L. J. Currell, M. Flores and W. Lubitz, *Biochim Biophys Acta Bioenerg*, 2010, **1797**, 304–313.
